# Time Scale-Dependent Structure–Property Relationships in Dynamic Imine-Benzoxazine Networks

**DOI:** 10.1021/acs.macromol.5c02664

**Published:** 2025-12-19

**Authors:** John J. Peyrefitte, Levi J. Hamernik, Elaina M. Booker, J. Drake Arrington, Jeffrey S. Wiggins

**Affiliations:** School of Polymer Science and Engineering, 5104 The University of Southern Mississippi, 118 College Dr. #5050, Hattiesburg, Mississippi 39406, United States

## Abstract

Dynamic polymer networks, or vitrimers, incorporate labile cross-links that enable topological rearrangement of the network via associative bond exchange, a process accelerated by the application of strain and heat. The linear viscoelasticity of vitrimers is governed by the behavior of the network at both short and long time scales, influenced by both local segmental motions and the kinetics of the dynamic exchange mechanism itself. Herein, we investigate the linear viscoelasticity of imine-containing benzoxazine (iBOX) networks, the majority of which possess glass transition temperatures (*T*_g_) exceeding 100 °C and resemble nondynamic thermosets used in structural applications. Nine iBOX monomers were synthesized by the condensation reaction of an aldehyde-functionalized benzoxazine precursor with a series of difunctional amines. Subsequent cationic ring-opening polymerization produced iBOX vitrimer networks with varying poly­(propylene oxide) (PPO), poly­(ethylene oxide) (PEO), and poly­(ethylene) (PE) backbone structures, each represented by three different molecular weights between cross-links. Time–temperature superposition (TTS) was applied to small-amplitude oscillatory shear (SAOS) and stress relaxation data to elucidate the influence of polymer architecture on viscoelastic response. The occurrence of multiple relaxation processes with distinct temperature dependences necessitated separate shift factors for short- and long-time dynamics, each exhibiting an Arrhenius temperature dependence and yielding different estimates of apparent activation energy (*E*_a_). Notably, the structural variations lead to nonuniform changes in *E*_a_ across time scales, which exemplifies the complexity of tuning exchange kinetics in dynamic polymer networks.

## Graphical abstract

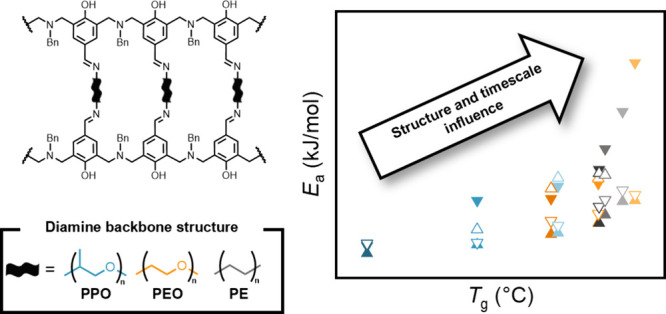

## Introduction

Dynamic covalent networks contain labile bonds that enable topological rearrangement of the network upon dynamic bond exchange. When dynamic exchange occurs through a dissociative mechanism, bond dissociation and reformation are distinct processes, resulting in a transient network with temporary reductions in cross-link density during topological rearrangement.^1^ In contrast, dynamic exchange through an associative mechanism, typically proceeding through addition–elimination pathways, maintains constant cross-link density, as bond dissociation and formation occur simultaneously.^2^ Vitrimers are defined by their use of associative exchange chemistries, which imparts them with mechanical robustness and solvent resistance resembling that of traditional cross-linked thermosets, while enabling thermoplastic-like processability, including reshaping, welding, and mechanical recycling through bond exchange.^3,4^

For traditional thermosets and thermoplastics, structure–property relationships are paramount to their performance in application. In vitrimers, the influence of structure on dynamic exchange adds an additional dimension of complexity to understanding structure–property relationships. Factors such as chain flexibility,^5,6^ backbone polarity,^7,8^ and steric hindrance^9,10^ have been found to influence exchange dynamics. This complexity is further amplified by the diversity of existing associative exchange chemistries, such as those based on imines,^7,11–20^ esters,^21–24^ boronic esters,^25–27^ siloxanes,^28–30^ and vinylogous urethanes,^8,31–33^ as well as the influences of both external small molecule catalysts^32,34^ and internal catalytic motifs that promote the covalent exchange.^21,24,35–38^

Imine-based vitrimers offer several advantages from a synthetic perspective, including the diversity of commercially available precursors, which enables rapid screening of diverse network architectures. Moreover, the dynamic exchange of imines does not require the presence of small-molecule catalysts, unlike some transesterification-based vitrimers.^39,40^ As a result, imine vitrimers are well-reported^7,11–20^ with many studies highlighting the tunability of network thermomechanical properties, as well as directing their application toward recyclable carbon-fiber-reinforced polymer structures.^40–46^ The formation of imine bonds, through the condensation of an aldehyde and a primary amine, results in the evolution of water.^47^ When network assembly proceeds by imine formation, the water byproduct makes it challenging to achieve high-quality, defect-free specimens. Thus, the rapid climb in viscosity in the bulk necessitates polymerization through solvent-casting from harsh organic solvents, which often requires lengthy drying processes and limits commercial applicability.^6,48,49^ To overcome these limitations, efforts have been made to realize imine networks through orthogonal polymerization methods, where imine formation occurs prior to cross-linking, such as in acrylate-^50^ and epoxy-based^46,51,52^ networks. We reported a platform for preparing imine-containing benzoxazine iBOX networks in which imine formation occurs during monomer synthesis, and cross-linking is facilitated by the condensate-free, cationic ring-opening polymerization of oxazine rings.^16^

The previously established iBOX platform allows for systematic structural changes, useful for investigations of structure–property relationships. Existing studies of highly cross-linked vitrimer networks have largely focused on reprocessing, healing, and identifying the onset of dynamic bond exchange,^48,53–56^ while more comprehensive rheological investigations have typically centered around loosely cross-linked systems that exhibit glass transition temperatures (*T*_g_s) near or below room temperature.^25,27,57,58^ In cases where *T*_g_ exceeds 100 °C, this is commonly achieved by the inherent rigidity of the parent homopolymer structures, such as dynamically cross-linked poly­(methyl methacrylate)^14^ or polystyrene,^15^ rather than high cross-linking density. However, thermosets traditionally used for composites, electronics, or other high-performance applications rely on glassy, amorphous polymer networks to achieve their thermal and mechanical properties. It is therefore paramount to understand the underlying exchange dynamics and viscoelasticity in highly cross-linked, high *T*_g_ vitrimers, for the rational design of next-generation thermoset replacements.

The linear viscoelastic behavior of vitrimers is often investigated using shear rheology through stress relaxation or small-amplitude oscillatory shear (SAOS) experiments. The former, whether in shear or not, has been used frequently to obtain an apparent activation energy, *E*_a_, which describes the temperature dependence or energy barrier for stress dissipation enabled by both topological rearrangement and segmental motion.^5,15,21,57,59–61^ Historically, the Maxwell model has often been applied indirectly to the stress relaxation of vitrimers for the estimation of *E*_a_^MM^.^5,21,31,32,61–67^ The value of *E*_a_^MM^ is estimated by determining the characteristic relaxation time τ^MM^), which corresponds to when stress reaches ∼37% (1/e) of the initial value. However, the applicability of this method has been criticized in more recent literature due to its oversimplification of the processes governing stress relaxation behavior.^15,59,60^ The validity of the method is predicated on the assumption that the Maxwell model accurately describes a material’s stress relaxation behavior.^60^ Since τ^MM^ can be determined without directly fitting the Maxwell model to all relaxation modulus curves, *E*_a_^MM^ is often estimated without the simultaneous assessment of this crucial assumption. As an alternative, the time–temperature superposition (TTS) principle has been applied to both stress relaxation and SAOS of vitrimers,^15,27,57,58,68–70^ where the temperature dependence of the horizontal shift factors can be used to determine *E*_a_.

Herein, we expand upon the previously established iBOX platform to investigate structure–property relationships through stress relaxation and SAOS experiments. In the present work, we probe the linear viscoelasticity of iBOX vitrimers, most of which resemble the glassy, amorphous nature of traditional, nondynamic thermosetting networks. We systematically vary polymer architecture by incorporating three backbone chemistries: poly­(propylene oxide) (PPO), poly­(ethylene oxide) (PEO), and poly­(ethylene) (PE), with three different molecular weights for each structure, allowing for the investigation of iBOX vitrimers having different *T*_g_, molecular weight between cross-links, and backbone polarity (Scheme 1). We leverage TTS and observe that two sets of shift factors are often required to superpose the data, which is consistent with existing studies due to the presence of multiple processes with different temperature dependences contributing to the linear viscoelasticity.^15,57^ In general, *E*_a_, estimated by fitting the Arrhenius equation to the horizontal shift factors, is observed to increase with *T*_g_, regardless of the shift factors used for the estimation.

**Scheme 1:**
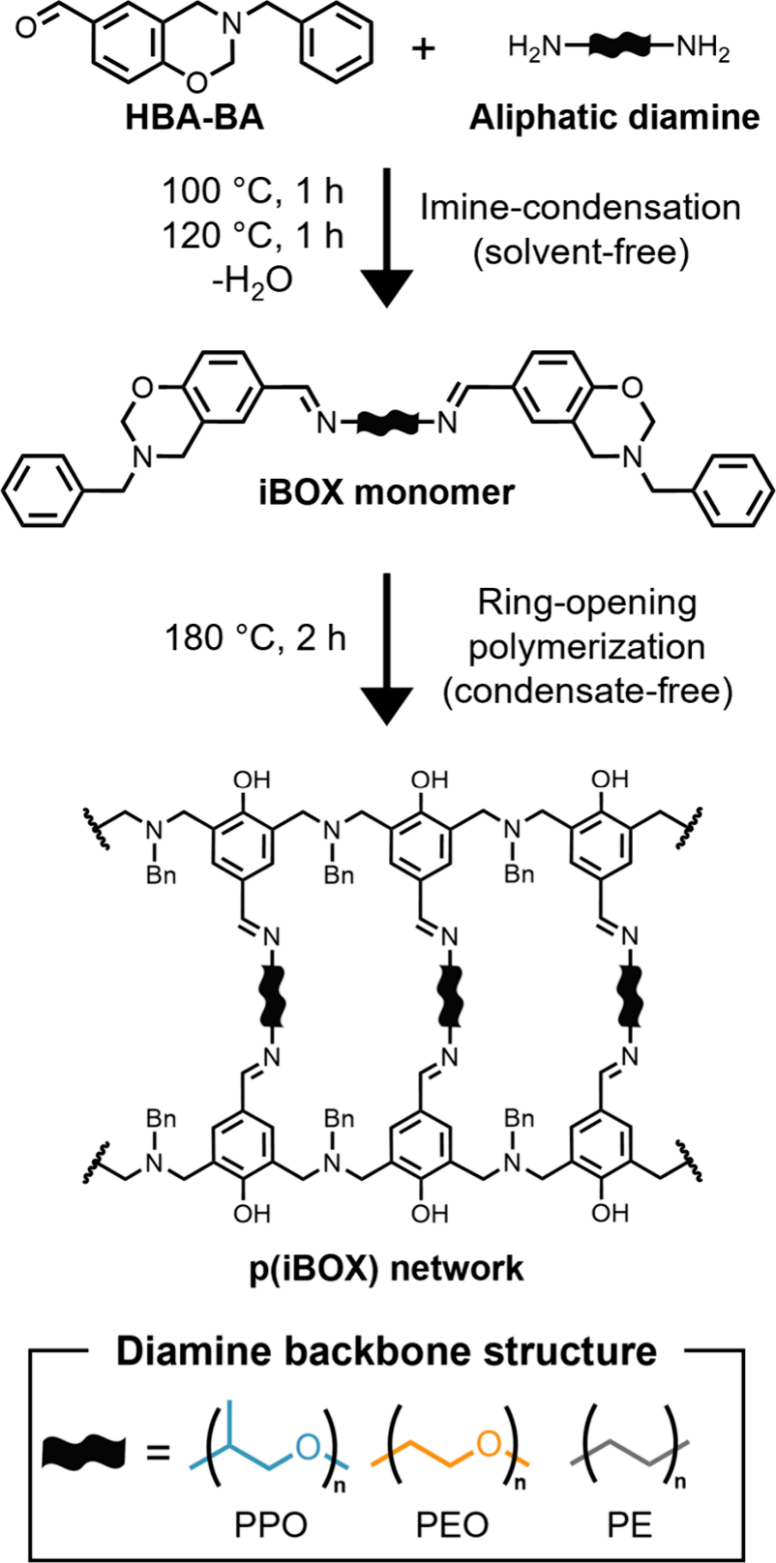
Formation of Imine-Containing Benzoxazine (iBOX) Monomers

## Materials and Methods

### Materials

Deuterated dimethylsulfoxide (DMSO-*d*_6_, 99.96%), deuterated chloroform (CDCl_3_, 99.99%), and aqueous formaldehyde (formalin, 37 wt %) were purchased from Millipore Sigma. 4-Hydroxybenzaldehyde (HBA, >99%) and 1,2-bis­(2-aminoethoxy)­ethane (PEO-148, >98%) were purchased from TCI America. Benzylamine (BA) (>99%) and 1,6-hexanediamine (PE-116, >99%) were purchased from Thermo Scientific Chemicals. Toluene (99.85%) and sodium hydroxide (NaOH, >97%) were purchased from Fisher Chemical. 2,2′-Oxydiethanamine (PEO-104, >98%), 3,3′-((oxybis­(ethane-2,1-diyl))­bis­(oxy))­bis­(propan-1-amine) (PEO-220, >95%), 1,8-diaminooctane (PE-144, 98%), and 1,10-diaminodecane (PE-172, 98%) were purchased from Ambeed Inc. Jeffamine polyetheramines D230, D400, and D2000 were acquired from Huntsman. All chemicals were used as received without further purification.

### Representative Synthetic Procedure for HBA-BA

The aldehyde-functionalized monofunctional benzoxazine monomer was synthesized according to previously established procedures.^16^ First, a one-neck 1 L round-bottom flask was charged with 181 g (2.2 mol) of aqueous formalin, and 1 M NaOH solution was added to achieve a pH of 9 before adding 400 mL of toluene. While stirring thoroughly with a magnetic stir bar, BA (108 g, 1.0 mol) was added dropwise. The mixture was allowed to stir for 1 h at 35 °C following complete addition of BA. Then, HBA (122 g, 1.0 mol) was added to the reaction mixture, which was subsequently heated to reflux and allowed to stir for 24 h. After cooling to room temperature, the organic phase was washed three times with 500 mL of deionized water before drying over anhydrous magnesium sulfate. The crude mixture was concentrated in a rotary evaporator to approximately 400 mL of total volume before refrigerating to accelerate crystallization. Following crystallization, the solid monomer was filtered and washed with cold toluene before drying in a vacuum oven at 40 °C overnight to afford a solid, white powder (237 g, 93% isolated yield, mp = 57 °C). ^1^H NMR (600 MHz, DMSO-*d*_6_) δ 9.80 (s, 1H), 7.69 (dd, *J* = 8.4, 2.1 Hz, 1H), 7.60 (d, *J* = 2.1 Hz, 1H), 7.42–7.33 (m, 2H), 7.33–7.31 (m, 2H), 7.30–7.22 (m, 1H), 7.0 (d, *J* = 8.4 Hz, 1H), 5.0 (s, 2H), 4.0 (s, 2H), 3.85 (s, 2H). ^13^C NMR (151 MHz, DMSO-*d*_6_) δ 191.2, 159.4, 137.9, 130.2, 129.5, 129.3, 128.5, 128.3, 127.3, 120.6, 116.7, 82.9, 54.6, 48.3. The ^1^H and ^13^C NMR spectra of HBA-BA are provided in Figures S1 and S2.

### Synthesis of iBOX Monomers

Each of the nine iBOX monomers was prepared in solvent-free conditions through the reaction of HBA-BA with the corresponding difunctional amine, as previously reported.^16^ In a representative case, HBA-BA (6.103 g, 24.09 mmol) was added as a solid to a 20 mL scintillation vial and melted at 100 °C. Then, PEO-148 (1.973 g, 13.31 mmol) was added to the scintillation vial in a dropwise fashion, and the mixture was stirred at 100 °C for 1 h. The temperature was increased to 120 °C, and the mixture was allowed to stir for 1 h under vacuum, which resulted in a pale-yellow, tacky amorphous solid upon cooling to room temperature (quantitative yield, *T*_g_ = 12.72 °C). ^1^H NMR (600 MHz, DMSO-*d*_6_) δ 8.2–8.0 (m, 2H), 7.6–7.2 (m, 17H), 6.9–6.7 (m, 2H), 5.0–4.8 (m, 4H), 4.1–3.2 (m, 30H). ^13^C NMR (151 MHz, DMSO-*d*_6_) δ 161.2, 160.9, 155.9, 138.1, 128.7, 128.5, 128.30, 128.26, 128.22, 128.1, 127.8, 127.6, 127.4, 127.2, 126.7, 120.1, 116.2, 116.1, 115.4, 82.3, 70.3, 70.2, 69.64, 69.55, 69.32, 63.9, 60.11, 60.08, 54.6, 51.8, 50.3, 49.3, 48.6. Synthetic procedures for all other iBOX monomers are discussed in Section S1 of the Supporting Information.

### Polymerization of iBOX Monomers

Cross-linked, iBOX polymer (p­(iBOX)) specimens were obtained by heating the monomers to 120 °C and pouring into high-temperature silicone molds. Monomers were held at 120 °C for 1 h in a vacuum oven at −28 inHg to remove trapped air. Then, the molds were transferred to a convection oven preheated to 120 °C, and the temperature was increased at a rate of 2 °C/min before holding the monomers at 180 °C for 2 h, except for iB-PPO-2000, which was held at 180 °C for 3 h.

### Spectroscopic and Thermal Characterization

Nuclear magnetic resonance (NMR) spectroscopy was performed using a Bruker Avance 600 MHz NMR spectrometer. Attenuated total reflectance Fourier-transform infrared (ATR-FTIR) spectroscopy was conducted using a PerkinElmer Frontier spectrometer. Thermogravimetric analysis (TGA) was conducted using a TA Instruments Q-50 TGA to obtain the degradation profiles in nitrogen, while a TA Instruments Discovery 5500 was used for experiments in air. In both cases, samples of ∼10 mg/mL were placed into platinum pans, and mass loss was monitored while heating to 900 °C at 10 °C/min. Step TGA experiments were performed to mimic the conditions of the rheological experiments (Figures S44–S46). Differential scanning calorimetry (DSC) measurements were obtained using a TA Instruments Discovery Series 2500 DSC using aluminum hermetic pans. Approximately 3 and 8 mg of the iBOX monomers and networks, respectively, were placed into pans and heated from −50 to 250 °C at a rate of 5 °C/min. Dynamic mechanical analysis (DMA) measurements in tension were obtained with a TA Instruments Q800 DMA using cross-linked rectangular specimens with dimensions of approximately 30 mm × 5 × 1 mm. Samples were heated from −100 to 220 °C at a rate of 3 °C/min while simultaneously applying an oscillatory strain at 1 Hz with an amplitude of 0.05%. The *T*_g_ was taken as the temperature corresponding to the maximum of tanδ.

### Rheological Measurements

Shear rheology was conducted using a TA Instruments ARES G2 strain-controlled rheometer equipped with a forced convection oven under air atmosphere. Samples were prepared by polymerizing iBOX monomers directly onto 8 mm stainless-steel parallel plates. Monomers were loaded on the plates at 100 °C, ramped at 2 °C/min to 180 °C, and held isothermally for 2 h to cure the network in situ, except for iB-PPO-2000, which was held at 180 °C for 3 h. Strain amplitude sweeps were performed by heating the polymerized sample to the lowest temperature employed for stress relaxation and small-amplitude oscillatory shear experiments. At a frequency of 10 rad/s, strain was logarithmically increased beginning at 0.01% to identify the linear viscoelastic regime. In the rubbery regime for each p­(iBOX) network, stress relaxation and small-amplitude oscillatory shear experiments were conducted at 5 °C intervals, and samples were allowed to equilibrate at each temperature for 600 s before data collection. Additional details are provided in Section S4 of the Supporting Information and Table S2. For stress relaxation experiments, a fixed shear strain of 1% was applied, and the relaxation modulus was monitored over 1000 s. Small-amplitude oscillatory shear experiments (SAOS) were conducted over a frequency range of 0.1 to 100 rad/s by applying an oscillatory shear strain of 0.1% in all cases except p­(iB-PPO-2000), which required a strain of 1.0% to produce adequate and repeatable torque due to the low modulus of the lightly cross-linked sample. For the SAOS results, TTS was applied by manually shifting the tanδ curves horizontally, and the same horizontal shifts were used for the storage (*G′*) and loss (*G″*) modulus curves in addition to conservatively applied vertical shifts, which were obtained by manually shifting the *G′* and *G″* curves. Separate sets of shift factors (*a*_SAOS_^short^, *b*_SAOS_^short^ and *a*_SAOS_^long^, *b*_SAOS_^long^) were obtained for the high- and low-frequency data, or short- and long-time scale behavior, respectively. Similarly, TTS was applied to the relaxation modulus (*G*) curves by manually shifting the curves horizontally and vertically, and separate sets of shift factors were obtained for the superposition of the low and high times (*a*_stress_^short^, *b*_stress_^short^ and *a*_stress_^long^, *b*_stress_^long^, respectively). Strain amplitude sweep, stress relaxation, and SAOS experiments were conducted on discrete, individually prepared samples, to minimize effects from accumulated thermal history.

## Results

### Synthesis and Spectroscopic Characterization of iBOX Monomers

The aldehyde-functionalized BOX monomer, HBA-BA, was synthesized and isolated according to previously established synthetic methods, achieving high purity and yield.^16^ Subsequently, each of the nine iBOX monomers were prepared through the melt-state reaction of HBA-BA and the corresponding difunctional amine. The conversion of aldehyde functional groups was monitored by the reduction of the −CHO band at 1690 cm^–1^ and the formation of the characteristic −CH=N– absorbance at approximately 1640 cm^–1^ (Figure 1 and Figures S33–S35). Monomer structure was confirmed through a series of ^1^H, ^13^C, APT, COSY, HSQC, and HMBC experiments (see Section S2 of the Supporting Information). For each employed diamine, the corresponding iBOX monomers were able to be efficiently synthesized through this solvent-free reaction, achieving quantitative yields, high aldehyde conversion, and preserving the oxazine functionality required for network formation.

**Figure 1:**
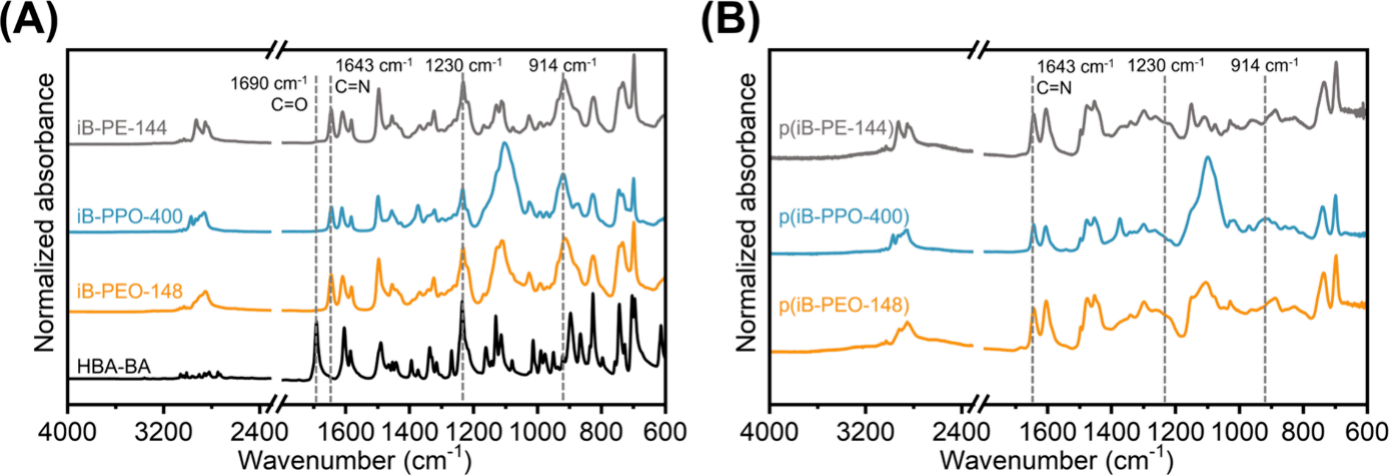
ATR-FTIR spectra of (A) iB-PE-144, iB-PPO-400, iB-PEO-148, and HBA-BA, and (B) the corresponding p­(iBOX) networks after thermally curing at 180 °C for 2 h.

### Spectroscopic and Thermal Characterization of p­(iBOX) Vitrimers

A total of nine p­(iBOX) networks were prepared, which will be referred to using the naming convention p­(iB-RP-X), where RP corresponds to the repeat unit structure (PPO, PEO, or PE) and X corresponds to the molecular weight (MW) of the corresponding diamine. Each of the nine iBOX monomers were thermally cured for 2 h at 180 °C to yield cross-linked networks, except in the case of p­(iB-PPO-2000), which required 3 h at 180 °C due to the low-concentration oxazine functional groups. For each network, high degrees of oxazine consumption are supported by (a) the consumption of the first exothermic peak in the DSC thermogram (Figures S37 and S43) and (b) the reduction of the characteristic absorbances at 1230 and 914 cm^–1^ in ATR-FTIR (Figure 1 and Figures S33–S35 and S38–S40). The cross-linked networks exhibited a lack of drastic mass loss until temperatures well above 200 °C by TGA (Figures S41 and S42).

Temperature ramp experiments by DMA indicated increasing *T*_g_ with decreasing molecular weight of diamine segments. The p­(iBOX) networks containing PPO segments, p­(iB-PPO-X), exhibit the broadest range of *T*_g_ values (−39.4–88.6 °C), while the PE-based p­(iBOX) networks, p­(iB-PE-X), exhibit the narrowest range of *T*_g_ values (116.1–131.8 °C) (Figure 2 and Table S1). The measured values of *T*_g_ for the p­(iB-PEO-X) networks ranged from 83.9 to 140.1 °C, and for all networks, the values are provided in Table S1. Overall, the p­(iB-PPO-X) networks possess lower *T*_g_s and rubbery plateau *E′* than the PEO- and PE-based networks. The theory of rubber elasticity was used to estimate cross-link density (Section S3 of the Supporting Information, Table S1), which resembles those reported for other imine networks with similar *T*_g_ values.^52,71^

**Figure 2:**
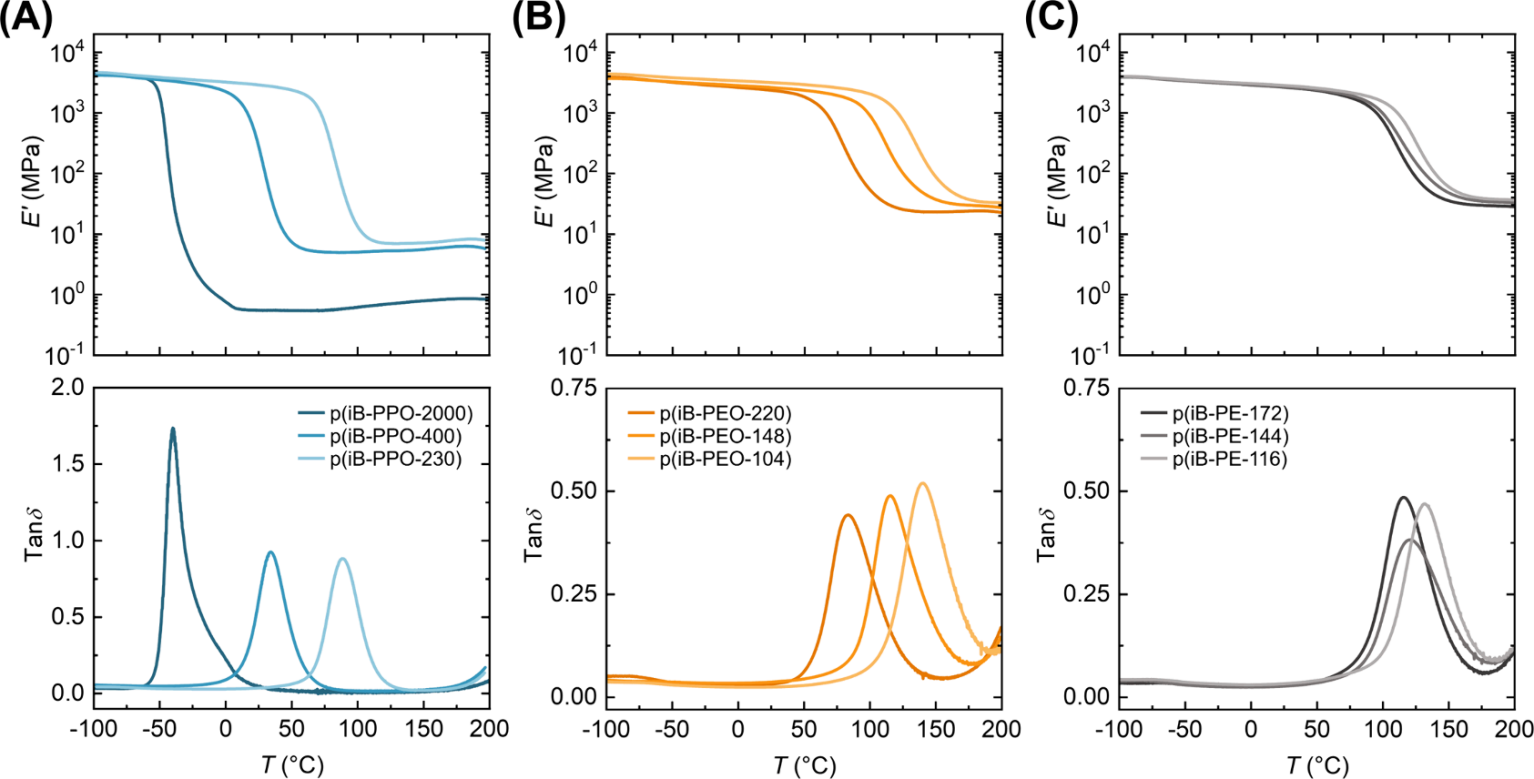
Dynamic mechanical analysis in tension for each (A) p­(iB-PPO-X), (B) p­(iB-PEO-X), and (C) p­(iB-PE-X) network, with storage modulus (*E′*) (top) and tanδ (bottom) plotted against temperature.

### Linear Viscoelasticity of p­(iBOX) Vitrimers by Shear Rheology

Strain amplitude sweeps at the lowest temperatures used for stress relaxation and SAOS indicated that an applied shear strain of 1% or below is well within the linear viscoelastic regime for all p­(iBOX) networks (Figure S47). Stress relaxation and SAOS were conducted in the rubbery regime for each p­(iBOX) network (Figures S48–S58 and S63–S71). In general, the stress relaxation curves obtained within the rubbery plateau for each p­(iBOX) network exhibit similar features (Figure 3A–C and Figures S51–S58). At short times, the relaxation modulus exhibits a gradual decrease before a more rapid decrease is observed at longer times. However, for p­(iB-PPO-2000), a plateau is observed in the relaxation modulus prior to the observed rapid decrease (Figure S55). At long times and high temperatures, the relaxation modulus exhibits a decrease in signal-to-noise for most of the networks due to torque limitations.

**Figure 3:**
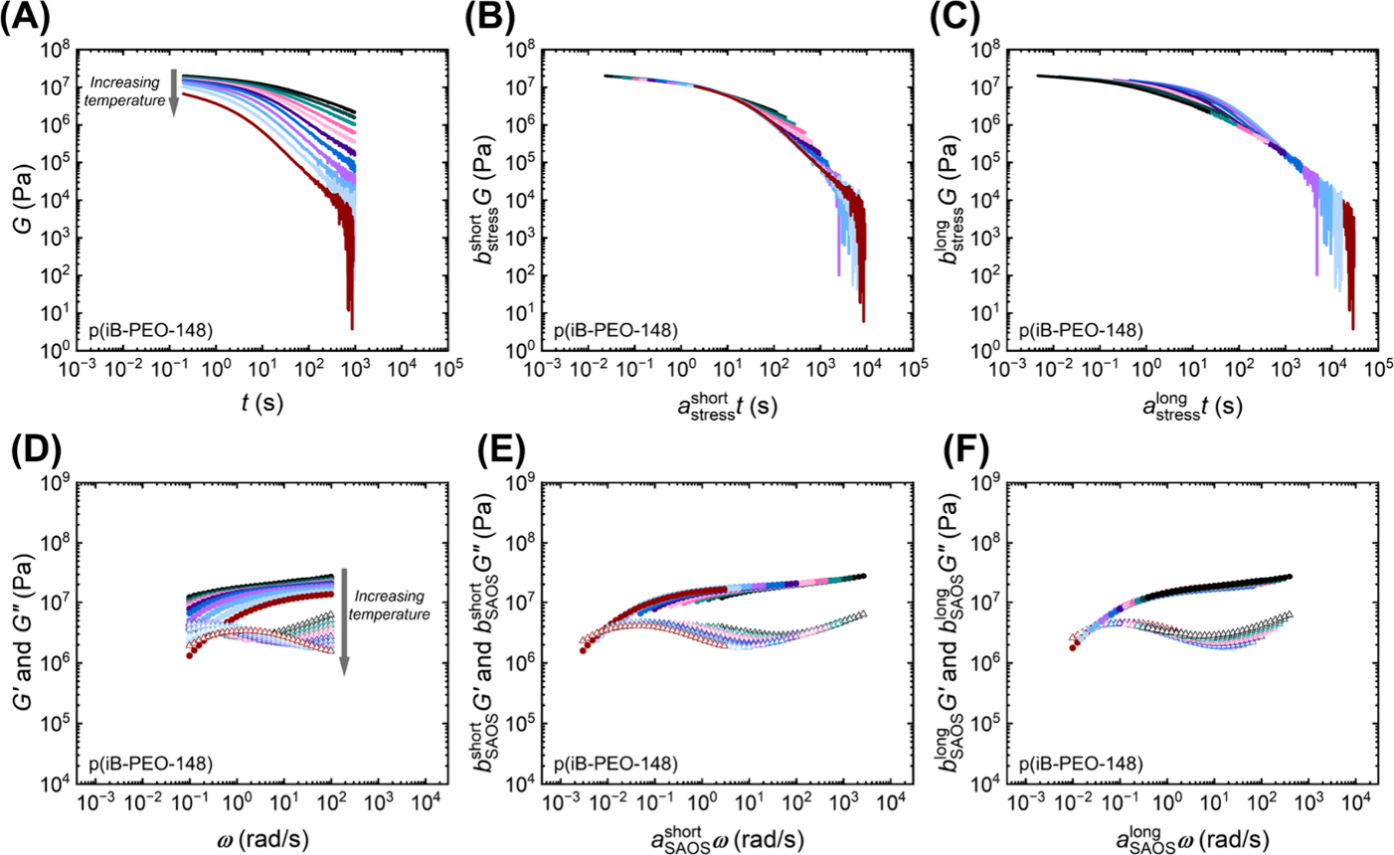
For p­(iB-PEO-148), relaxation modulus (*G*) plotted against time as (A) directly measured, (B) with *a*_stress_^short^ and *b*_stress_^short^ applied, and (C) with *a*_stress_^long^ and *b*_stress_^long^ applied. Shear storage (*G′*) and loss (*G″*) moduli curves plotted against angular frequency (ω) (D) as obtained, (E) with *a*_SAOS_^short^ and *b*_SAOS_^short^ applied, and (F) with *a*_SAOS_^long^ and *b*_SAOS_^long^ shifts applied. *T*_ref_ = 175 °C.

Small-amplitude oscillatory shear (SAOS) experiments were performed at the same temperatures used for stress relaxation. As angular frequency (ω) increases, tanδ initially exhibits a decrease before reaching a local minimum and increasing with frequency for all networks (Figure 3D–F and Figures S63–S71). Generally, the storage modulus (*G*′) curves exhibit an increase in magnitude before plateauing or increasing gradually with ω. However, the prominence of the plateau in *G*′ is observed to decrease with decreasing molecular weight (MW) of diamine segments. With increasing ω, for most networks, the loss modulus (*G*″) displays a slight peak before exhibiting a minimum and subsequently increasing. Although some of the networks exhibit crossover of *G*′ and *G*″, power-law fits were not applied to assess Rouse-like behavior due to the small number of data points at frequencies below crossover.

### Time–Temperature Superposition of p­(iBOX) Networks

In most cases for stress relaxation and all cases for SAOS (Figure 3 and Figures S51–S58 and S63–S71), the curves were observed to superpose at short and long time scales, respectively, requiring separate shift factors for both the high- and low-frequency regimes in SAOS and for the low- and high-time regimes in stress relaxation. The tanδ curves were manually superposed at the short and long time scales to obtain the same horizontal shifts used to superpose the *G*′ and *G*″ curves (Figure S72). Subsequently, vertical shifts were conservatively applied to the *G*′ and *G*″ curves (Figure S73). The stress relaxation curves were manually superposed to obtain both horizontal and vertical shift factors (Figures S59 and S60). While most of the networks had two separately superposable regimes in their respective *G* curves, p­(iB-PEO-104), p­(iB-PE-116), and p­(iB-PPO-2000) demonstrated superposition across all time scales, leading to a single *G* master curve for each network (Figures S51, S56, and S55). The latter network corresponds to the highest diamine MW and lowest *T*_g_, while the former two networks correspond to the lowest diamine MWs and highest *T*_g_s.

For networks not demonstrating superposition across all time scales, the long-time portions (>500 s) of the *G* curves were superposed manually with shift factors separate to those for superposing the short-time regimes. The manual superposition of the *G* curves at long times, through the application of *a*_stress_^long^ and *b*_stress_^long^, led to pseudomaster *G* curves (Figure 3C) resembling those reported for a dioxaborolane system,^57^ where superposition of *G* at long times was achieved with horizontal shifts obtained by superposing creep data. For the six p­(iBOX) networks in which this practice was used, an intermediate regime in the pseudomaster curves was observed, where the shorter time scale regimes of the *G* curves deviated from the pseudomaster curve (Figure 4D,E). This was also apparent when superposing the curves with *a*_stress_^short^ and *b*_stress_^short^, except with the high-time portions of the curves deviating from the pseudomaster curves (Figure 4A–C). When comparing the pseudomaster curves of all networks in order of diamine MW, or *T*_g_, it is observed that the deviation at intermediate time scales is less prominent for networks nearing the upper and lower extremes of diamine MW across the nine networks, where the stress relaxation master curves began to resemble those which superimpose over the full range of time scales (Figures S54 and S57).

**Figure 4:**
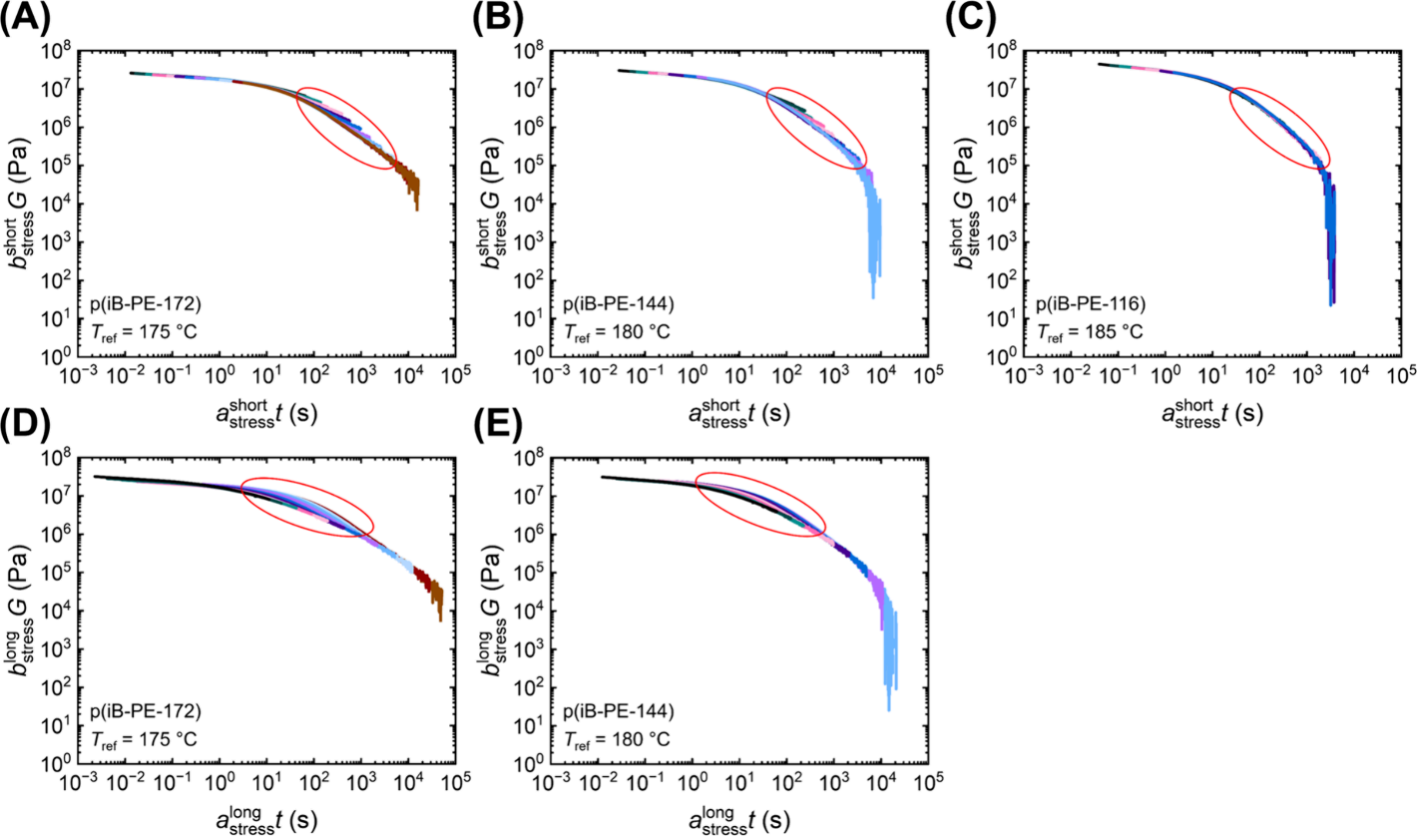
Stress relaxation curves for the p­(iB-PE-X) networks in order of decreasing diamine MW (A–C) with *a*_stress_^short^ and *b*_stress_^short^ applied, and (D, E) with *a*_stress_^long^ and *b*_stress_^long^ applied. Red ovals highlight deviation from the pseudomaster curves.

Each set of horizontal shift factors exhibited an Arrhenius temperature dependence. Thus, apparent activation energies (*E*_a_) were estimated for each set of horizontal shift factors with a linearized form of the Arrhenius equation:

$$\ln(a_{\mathrm{method}}^{\mathrm{time}\;\mathrm{scale}})=\frac{E_{a,\mathrm{method}}^{\mathrm{time}\;\mathrm{scale}}}{R}\left(\frac{1000}{T}\right)+\mathrm{intercept}$$ 1

where *a*_method_^time scale^ represents the horizontal shift factor according to the experimental method (either “stress” for stress relaxation or SAOS) and time scale (either short or long), *R* is the gas constant, and *T* is temperature (Table S3). For a given material, *E*_a,stress_^short^ and *E*_a,SAOS_^long^ are generally lower than *E*_a,stress_^long^ and *E*_a,SAOS_^short^, which is evident when comparing the slopes for each of the linear fits (Figure 5). In general, the estimates of *E*_a_ increase with decreasing diamine MW.

**Figure 5:**
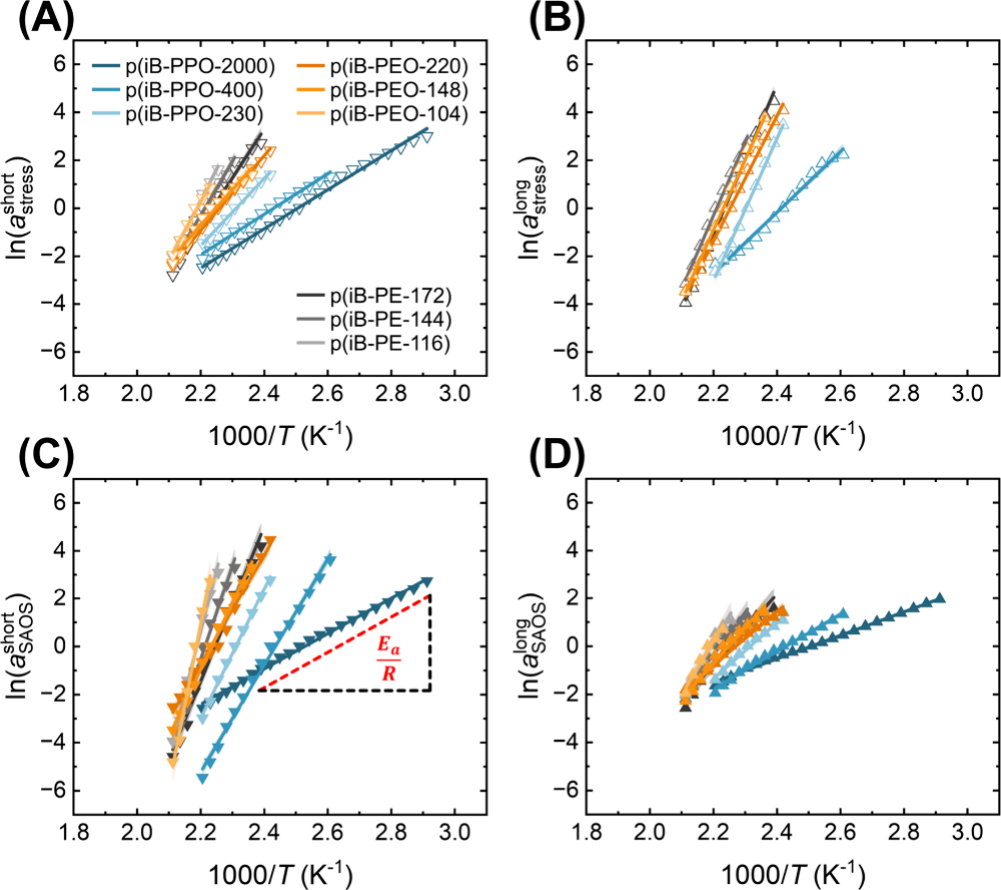
Horizontal shift factors and corresponding Arrhenius fits (with 95% confidence intervals for the slope of the linear fit) for (A) stress relaxation at short time scales, (B) stress relaxation at long time scales, (C) SAOS at short time scales, and (D) SAOS at long time scales.

The influence of network structure on the linear viscoelasticity of p­(iBOX) networks was investigated by shear rheology. The TTS principle was applied to the dynamic moduli (*G′* and *G″*) and relaxation modulus (*G*) curves. In most cases, two sets of shift factors were required to superpose the curves at short and long time scales, respectively. All shift factors exhibited an Arrhenius temperature dependence, enabling the estimation of *E*_a_. The estimates of *E*_a_ varied according to both network structure and the time scale corresponding to the estimate.

## Discussion

The inherent modularity of the iBOX platform enabled elucidation of structure–property relationships by emerging rheological methods, allowing for comparisons based on backbone segment length, polarity, and steric hindrance. Nine iBOX monomers were prepared through the condensation reaction of an aldehyde-functionalized benzoxazine monomer with nine different difunctional amines. The iBOX monomers were polymerized through the cationic benzoxazine ring-opening polymerization, leading to networks with *T*_g_s ranging from −39 to 140 °C. The observed changes in *E*_a_ indicate the nuanced interplay between structure and vitrimer properties, since the influence on dynamics is not identical at long and short time scales.

### Time scale-Dependent Estimation of Apparent Activation Energy

With the pseudomaster curves constructed by aligning different portions of the moduli curves, the approximate range of corresponding time scales must be considered when comparing the estimated *E*_a_ values. The reciprocal (∼0.01 to ∼0.02 s) of the approximate short time scale range for SAOS (∼50 to 100 rad/s) is considerably shorter than the shortest time scales used to superpose the *G* curves from stress relaxation (>0.1 s). However, for the approximate long time scale range in SAOS, the reciprocal (∼0.3 to 10 s) of ∼0.1 to 3 rad/s resembles the shortest time scales observed in stress relaxation. In contrast, the approximate time scale used to superpose the long-time *G* data from stress relaxation (∼500 to 1000 s) is considerably longer than all other time scales used to estimate *E*_a_. Thus, *E*_a,SAOS_^short^ and *E*_a,stress_^long^ respectively describe the temperature dependences corresponding to the shortest and longest time scales investigated herein. The estimated values of *E*_a_ in order of increasing time scale are as follows: *E*_a,SAOS_^short^ < *E*_a,stress_^short^ ∼ *E*_a,SAOS_^long^ < *E*_a,stress_^long^. Each estimate of *E*_a_ is provided in Table S3, and estimates of *E*_a_^MM^ were included for comparison, which most closely resemble *E*_a,stress_^short^ and *E*_a,SAOS_^long^.

Shorter time scales have been associated with the fast dynamics corresponding to segmental motions.^57,72^ It has been proposed that the slow dynamics, prominent at long time scales, are influenced by factors such as the diffusion of the dynamic bonds and matrix effects such as backbone polarity and flexibility.^7,15^ For the p­(iBOX) networks herein, the individual contributions of the fast and slow dynamics are convoluted, particularly in stress relaxation experiments, and the extent of their contributions at different time scales varies between the p­(iBOX) networks. In the pseudomaster curves obtained by aligning *G* at short and long time scales, the observed deviation at intermediate time scales varies between networks. For example, when comparing the p­(iB-PE-X) networks, the prominence of this deviation appears to increase with PE diamine molecular weight (Figure 4), while the reverse is observed for the p­(iB-PPO-X) networks (Figures S53–S55). In both cases, the reduction in the deviation at intermediate time scales results in only needing one set of shift factors to superpose *G* for p­(iB-PE-116) and p­(iB-PPO-2000). Although the origin of this result is not clear, the superposition of *G* with a single set of shift factors resembles what was observed for previously reported polystyrene-based imine vitrimers,^15^ while the presence of two separate superposable regions requiring two sets of shift factors to superpose *G* resembles that for previously reported dioxaborolane vitrimers.^57^ In both reports and for the present p­(iBOX) networks, two distinct superposable regimes in the *G′* and *G″* curves obtained through SAOS are simultaneously observed.

When comparing the *E*_a_ values estimated by superposing the SAOS data at short and long time scales (Figure 6), the shorter time scales are associated with a significantly greater temperature dependence than the longer time scales, as indicated by *E*_a,SAOS_^short^ and *E*_a,SAOS_^long^ (Figure 5), which compares favorably to previously reported polystyrene-imine vitrimers.^15^ The heightened temperature dependence is attributed to the increased influence of segmental motions at shorter time scales. This is supported by the estimates of *E*_a,SAOS_^short^ that exceed 300 kJ/mol for p­(iB-PE-144), p­(iB-PE-116), and p­(iB-PEO-104), where restricted segmental mobility at time scales on the order of ∼10 ms is attributed to high cross-link density (as estimated by the classical theory of rubbery elasticity) (Tables S1 and S3). Overall, a decrease in *E*_a_ is observed as the corresponding time scale increases when comparing *E*_a,SAOS_^short^, *E*_a,stress_^short^, and *E*_a,SAOS_^long^. The estimated values of *E*_a,stress_^short^ and *E*_a,SAOS_^long^ are generally similar to one another, which is attributed to the similarity in the corresponding time scales for each estimate. However, the decrease in *E*_a_ with increasing time scale is not observed when also considering *E*_a,stress_^long^, which we attribute to the decreased prominence of chain perturbation during continuous application of a step strain. Although *E*_a,SAOS_^short^ and *E*_a,stress_^long^ respectively correspond to the shortest and longest time scales discussed herein, the two values are often both similar and higher than both of the *E*_a_ values corresponding to more intermediate time scales (*E*_a,stress_^short^ and *E*_a,SAOS_^long^) for a given network. The observed similarity in the estimates at the opposite extremes of corresponding time scales (*E*_a,SAOS_^short^ and *E*_a,stress_^long^) illustrates the multifaceted nature of comparing *E*_a_ values coinciding with different time scales for superposition. In addition to the potential influence of corresponding time scale, the nature of the applied strain is particularly relevant in using TTS to estimate *E*_a_.

**Figure 6:**
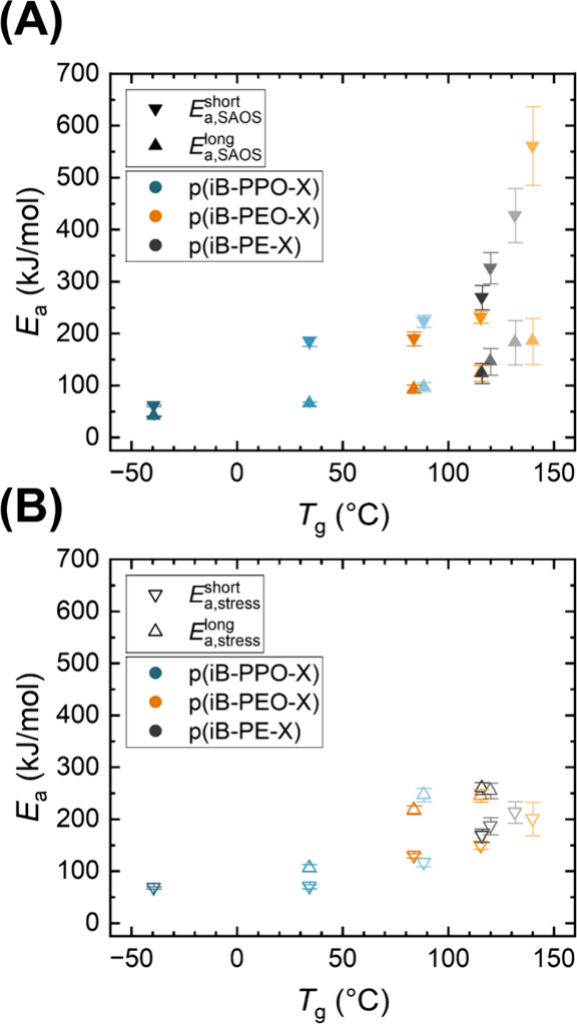
Estimates of *E*_a_ corresponding to (A) SAOS and (B) stress relaxation plotted against *T*_g_ (taken as where tanδ reaches a maximum in DMA), with the color for each backbone structure being darker for higher diamine MW. Error bars represent the 95% confidence intervals of the Arrhenius fits.

Upon the application of a step strain during stress relaxation, an imine-based vitrimer network will accommodate for the resulting stress through topological rearrangement. Unlike in the SAOS experiments where an oscillatory strain is applied at rates ranging from approximately 60 s or <0.1 s, in stress relaxation, the network is allowed to accommodate a strain applied constantly over a given time (in the present work, 1000 s). Dynamic exchange reactions in vitrimers typically occur through addition–elimination pathways, and for imine-based vitrimers, recent literature supports that dynamic exchange occurs predominantly through transamination, rather than imine metathesis.^13^ Transamination occurs when imine functional groups undergo nucleophilic attack by a primary amine within the network, leading to the formation of a tetrahedral intermediate. Elimination of the primary amine originally acting as a nucleophile, rather than the original constituent of the imine bond, does not result in topological rearrangement (topology-preserving exchange). When stress accumulates upon the application of strain, we hypothesize that the realization of thermodynamically unfavorable chain conformations, or chain perturbation, leads to preferential elimination of one amine constituting the tetrahedral intermediate. Upon elimination of the original amine constituting the imine (topology-altering exchange), topological rearrangement occurs. Moreover, when the apparent stress resulting from an applied strain is alleviated through topological rearrangement over longer periods of time, there should be an increased prevalence of topology-preserving exchange events, or a reduction in exchange events overall. Thus, we hypothesize that at long times in stress relaxation (>500 s), the corresponding estimate of apparent activation energy, *E*_a,stress_^long^, is elevated by the overall reduction of chain perturbation in the network.

### Comparisons of Estimated Activation Energy According to Network Structure

Out of the series of p­(iBOX) networks herein, the p­(iB-PPO-X) networks are associated with the largest range of *T*_g_s and diamine MW. Thus, the p­(iB-PPO-X) networks are most suitable for comparisons based on significant differences in diamine MW relative to one another. As the number of PPO repeat units decreases, *E*_a_ is observed to increase regardless of the method corresponding to the estimation. The present observations agree with a previously reported comparison of PPO-based imine networks, where *E*_a_ was found to increase with reducing molecular weight between cross-links.^73^ Compared to all other p­(iBOX) networks presented herein, p­(iB-PPO-2000) exhibited *E*_a_ values (∼40–70 kJ/mol) most comparable to those previously reported for solution-state imine exchange in small-molecule systems (∼33–37 kJ/mol).^12,74^ In contrast, the estimated *E*_a_ values for p­(iB-PPO-230) (Figure 6) are more comparable to the other glassy networks presented herein, such as the lower-*T*_g_ p­(iB-PEO-X) and p­(iB-PPO-X) networks, which we attribute to the similarity in molecular weight of diamine segments.

The PPO- and PEO-based networks were selected to probe the impact of steric hindrance on dynamic bond exchange. The p­(iB-PPO-230) network most closely resembles p­(iB-PEO-220) in terms of *T*_g_. However, p­(iB-PPO-230) exhibits a much lower rubbery modulus than p­(iB-PEO-220) (Figure 7A and Table S1). Statistically significant changes in *E*_a_ are observed at the shortest and longest time scales, where in both cases, p­(iB-PPO-230) is observed to have a greater *E*_a_ (Figure 6). Given that *E*_a,SAOS_^short^ corresponds to short time scales, like those associated with segmental motions, we attribute the apparent increase to the greater rigidity of the PPO repeat unit caused by the pendent methyl group. Furthermore, we attribute the increased *E*_a,stress_^long^ for p­(iB-PPO-230) to the increased steric hindrance associated with the pendent methyl group adjacent to the imine bond, resulting in a higher energy barrier to form the tetrahedral intermediate in the addition–elimination exchange reaction. Additionally, the normalized relaxation modulus curves for p­(iB-PPO-230) indicate a more significant decrease in relaxation modulus prior to long time scales (>500 s), which supports the presence of less chain perturbation compared to p­(iB-PEO-220), leading to a greater estimate of *E*_a,stress_^long^ for the former (Figure S61).

**Figure 7:**
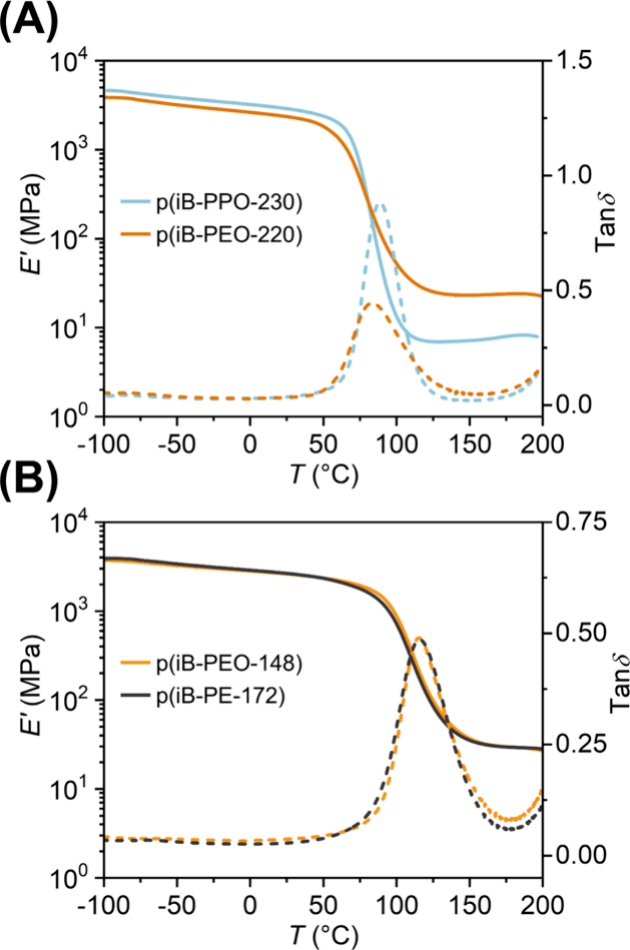
Tensile storage modulus (*E*′) and tanδ plotted against temperature for (A) p­(iB-PPO-230) and p­(iB-PEO-220), and (B) p­(iB-PEO-148) and p­(iB-PE-172).

Similar to the comparisons between the three p­(iB-PPO-X) networks, the *E*_a_ values for the p­(iB-PEO-X) networks are observed to increase with decreasing diamine segment MW, independent of the method corresponding to the estimation of *E*_a_. Out of all nine p­(iBOX) networks, p­(iB-PEO-104) was consistently observed to have the highest *E*_a_ value in each method, which we attribute to it having the lowest diamine segment MW. Both backbone flexibility and polarity will change through modulation of PEO repeat unit length. As the PEO segment length decreases, the influence of decreased backbone flexibility on viscoelasticity is supported by the increase in *E*_a_ estimated at the shorter time scales, since these time scales are especially influenced by segmental motions. Backbone polarity has previously been suggested as an influence on exchange kinetics in imine^7^ and other vitrimer networks,^8^ and since solution-state dynamic exchange of small-molecule imine compounds is accelerated by solvent polarity,^75,76^ the observed increase in *E*_a,stress_^long^, though only obtained for p­(iB-PEO-220) and p­(iB-PEO-148), supports that backbone polarity may have some influence on exchange kinetics. However, the increase in *E*_a,stress_^long^ was not as drastic as the increases observed in the *E*_a_ values corresponding to shorter time scales. We speculate that this is due to one or more factors such as (a) backbone flexibility having a greater impact at shorter time scales than backbone polarity does at long time scales, (b) the changes in PEO segment length not being drastic enough to result in large changes in polarity relative to the other polar functional groups present from benzoxazine ring-opening (i.e., phenolic species, tertiary amines, etc.), and (c) convolution with the chain perturbation arguments presented previously.

The p­(iB-PEO-X) and p­(iB-PE-X) networks were selected to more rigorously compare differences in backbone polarity between two networks than in the comparison of different PEO repeat unit lengths. While p­(iB-PEO-148) and p­(iB-PE-144) have identical structures other than the presence or absence of two ether groups, the inclusion of the ether groups led to a notable decrease in *T*_g_ (Figure 2). However, the increased flexibility of the longer alkyl backbone in p­(iB-PE-172) led to an approximately equivalent *T*_g_ and more similar rubbery plateau *G*′ to that of p­(iB-PEO-148), enabling comparisons independent of these parameters (Figure 7B and Table S1). When comparing the estimates of *E*_a_ in order of time scale, p­(iB-PE-172) exhibits a greater *E*_a_ than p­(iB-PEO-148), and the disparity decreases when comparing *E*_a,SAOS_^short^, *E*_a,stress_^short^, and *E*_a,SAOS_^long^ in order. This suggests that the presence of the more polar and flexible PEO repeat unit impacts linear viscoelasticity at short time scales due to the increase of chain flexibility. For p­(iB-PE-172), the reduced backbone polarity of the alkyl backbone is believed to elevate *E*_a,stress_^long^ by slowing imine exchange kinetics. In contrast, its higher normalized relaxation modulus at long times (>500 s) is expected to slightly depress *E*_a,stress_^long^ through the increased prominence of topology-preserving exchange events due to lessened chain perturbation. Since *E*_a stress_^long^ is ultimately greater for p­(iB-PE-172), we conclude that the influence of backbone polarity dominates over the effect of differing extents of chain perturbation at long time scales. Overall, the observed differences in *E*_a_ estimated at different time scales support that even with approximately equivalent *T*_g_ and rubbery plateau modulus, *E′*, the linear viscoelasticity does not change uniformly with structure across different time scales. These findings highlight the complexity of structure–property relationships in dynamic polymer networks, which has significant implications for their application as recyclable and repairable thermosets.

When comparing the p­(iB-PE-X) networks, *E*_a_ is found to increase with decreasing PE segment length in all cases except for *E*_a,stress_^long^ for p­(iB-PE-144) and p­(iB-PE-172), which exhibits no change and corresponds to long time scales. Aside from the lack of change in *E*_a,stress_^long^, the observed trends in *E*_a_ are reminiscent of those observed for both the p­(iB-PPO-X) and p­(iB-PEO-X) networks. The observed increase in *E*_a_ with decreasing PE segment length becomes more prominent as the corresponding time scale for the estimate becomes shorter. With the modulation of PE segment length, the lack of change in *E*_a,stress_^long^ suggests that the subtle changes in backbone polarity and diamine MW do not contribute significantly to the exchange behavior at long time scales, which coincides with the comparisons between the p­(iB-PEO-X) networks themselves.

## Conclusions

Expanding upon our previous work, nine imine-containing p­(iBOX) vitrimer networks were prepared with varied structures to further demonstrate the tunability of network properties and facilitate a systematic investigation of linear viscoelastic behavior according to structure. The networks were varied according to incremental changes in the respective diamine used to synthesize each iBOX monomer, allowing for comparisons of PEO-, PPO-, and PE-based structures of different molecular weights. Stress relaxation and SAOS experiments were conducted to probe the influence of p­(iBOX) network structure on rheological behavior.

Superposition of *G*′, *G″*, and *G* was observed for all networks; however, for all *G*′, *G″* curves and most *G* curves, two sets of shift factors were required to superpose the curves at different time scales. Each set of shift factors exhibited an Arrhenius temperature dependence, which enabled the estimation of *E*_a_ in all cases. For a given p­(iBOX) network, the disparities observed between each of the estimates support the presence of multiple, distinct processes contributing to the viscoelastic behavior at different time scales, and *E*_a,SAOS_^short^ and *E*_a,SAOS_^long^ were typically associated with the highest and lowest magnitudes, respectively, for a given material. When comparing between p­(iBOX) networks, changes in the estimated values of *E*_a_ were attributed to backbone flexibility, steric hindrance, and backbone polarity, which was corroborated by the relative time scales corresponding to the affected estimates of *E*_a_ for each network. The observed influence of structure was not uniform across all estimates of *E*_a_, pointing to the significant intricacy of structure–property relationships in vitrimers. Regardless, we hypothesize that a decrease in chain perturbation, or increase in topology-preserved exchange events, leads to an increased energy barrier for additional stress relaxation, as indicated by the elevated values of *E*_a,stress_^long^. We believe this occurs when the network is allowed to alleviate stress through topological rearrangement over a prolonged period (approaching 1000 s or more in the present cases). Overall, the present findings exemplify the added complexity of elucidating structure–property relationships when dynamic bonds are present in cross-linked polymer networks, which will serve as a significant hurdle in the utilization of vitrimers as recyclable alternatives to conventional thermosets. Future analyses of structure–property relationships in vitrimers should consider that structural changes may have separate, yet potentially intertwined, impacts on network behavior at different time scales, as well as how this impacts reprocessing efficacy.

## Supplementary Material



## Notes

Synthesis of iBOX monomers, NMR spectroscopy of benzoxazine monomers, ATR-FTIR spectroscopy of iBOX monomers and p­(iBOX) networks, thermogravimetric analysis (TGA) of iBOX monomers and p­(iBOX) networks, differential scanning calorimetry (DSC) of iBOX monomers and p­(iBOX) networks, strain amplitude sweep experiments, temperature conditions for rheological experiments, stress relaxation of p­(iBOX) networks, horizontal and vertical shift factors for superposing the relaxation modulus curves, normalized stress relaxation curves, small-amplitude oscillatory shear (SAOS) of p­(iBOX) networks, horizontal and vertical shift factors for superposing the tanδ and shear moduli curves, and estimates of apparent energy of activation (*E*_a_) for each p­(iBOX) network (PDF)
