# Supplementary material for: Time Scale-Dependent Structure–Property Relationships in Dynamic Imine-Benzoxazine Networks
Source: Macromolecules. 2025 Dec 19;59(1):503–14. doi: 10.1021/acs.macromol.5c02664 (PMC12805638; doi:10.1021/acs.macromol.5c02664)
Supplement: Supplementary file 1 [file ma5c02664_si_001.pdf]

Supporting Information

Timescale-dependent structure-property relationships in dynamic imine-benzoxazine networks

John J. Peyrefitte[a], Levi J. Hamernik[a], Elaina M. Booker[a], J. Drake Arrington Jr.[a], Jeffrey S. Wiggins[a,*]

[a]School of Polymer Science and Engineering, The University of Southern Mississippi, 118 College Dr. #5050, Hattiesburg, Mississippi 39406, United States

Table of Contents

S1. Synthesis of iBOX monomers....3

Synthesis of iB-PPO-230....3

Synthesis of iB-PPO-400....3

Synthesis of iB-PPO-2000....3

Synthesis of iB-PE-116....4

Synthesis of iB-PE-144....4

Synthesis of iB-PE-172....4

Synthesis of iB-PEO-104....4

Synthesis of iB-PEO-220....5

S2. Spectroscopic and Thermal Analyses of iBOX Monomers....6

Nuclear Magnetic Resonance (NMR) Spectroscopy of HBA-BA....6

NMR Spectroscopy of iBOX Monomers....7

Attenuated Total Reflectance Fourier-Transform Infrared (ATR-FTIR) of Benzoxazine Monomers....22

Thermogravimetric Analysis and Differential Scanning Calorimetry (DSC) of Benzoxazine Monomers....23

S3. Spectroscopic and Thermal Analyses of p(iBOX) Networks....24

ATR-FTIR of p(iBOX) Networks....24

Thermogravimetric Analysis and Differential Scanning Calorimetry of p(iBOX) Networks....26

Dynamic Mechanical Analysis....29

S4. Rheological Characterization 30
Strain Amplitude Sweep Experiments for p(iBOX) Networks 30
Temperature Conditions for Rheological Experiments 31
Stress Relaxation of p(iBOX) Networks 34
Small-Amplitude Oscillatory Shear (SAOS) of p(iBOX) Networks 39
Estimation of Apparent Activation Energy 49

S1. Synthesis of iBOX monomers

Synthesis of iB-PPO-230

HBA-BA (5.266 g, 20.79 mmol) was added as a solid to a 20 mL scintillation vial and melted at 100 °C. In a dropwise fashion, PPO-230 (2.758 g, 11.43 mmol) was added to the scintillation vial, and the mixture was stirred at 100 °C for 1 h. Then, the temperature was increased to 120 °C, and the mixture was allowed to stir for 1 h under vacuum, which resulted in a pale yellow, tacky amorphous solid upon cooling to room temperature (quantitative yield, T_g = 9.35 °C). ^{1}H NMR (600 MHz, DMSO-d_6) δ 8.25 – 8.00 (m, 2H), 7.63 – 7.11 (m, 15H), 6.90 – 6.58 (m, 2H), 4.90 (d, J = 8.0 Hz, 3H), 4.04 – 3.74 (m, 8H), 3.72 – 3.03 (m, 18H), 1.24 – 0.70 (m, 14H). ^{13}C NMR (151 MHz, DMSO-d_6) δ 159.2, 159.1, 155.9, 138.1, 128.8, 128.7, 128.5, 128.3, 128.0, 127.8, 127.6, 127.5, 127.44, 127.41, 127.2, 120.1, 116.1, 115.3, 82.2, 75.4, 74.5, 74.30, 74.25, 74.2, 74.1, 73.5, 73.3, 65.1, 65.0, 64.58, 64.55, 64.5, 54.6, 51.8, 48.6, 19.01, 18.96, 18.89, 18.86, 18.83, 18.80, 17.2, 17.1, 17.0.

Synthesis of iB-PPO-400

HBA-BA (3.753 g, 14.82 mmol) was added as a solid to a 20 mL scintillation vial and melted at 100 °C. In a dropwise fashion, PPO-400 (3.742 g, 8.135 mmol) was added to the scintillation vial, and the mixture was stirred at 100 °C for 1 h. Then, the temperature was increased to 120 °C, and the mixture was allowed to stir for 1 h under vacuum, which resulted in a pale yellow, tacky viscous liquid upon cooling to room temperature (quantitative yield, T_g = -16.80 °C). ^{1}H NMR (600 MHz, DMSO-d_6) δ 8.16 (d, J = 3.5 Hz, 2H), 7.74 – 7.11 (m, 15H), 6.94 – 6.64 (m, 2H), 4.89 (s, 3H), 4.16 – 3.00 (m, 40H), 1.42 – 0.59 (m, 26H). ^{13}C NMR (151 MHz, DMSO-d_6) δ 159.1, 155.9, 138.1, 128.8, 128.5, 128.3, 128.1, 127.54, 127.50, 127.42, 127.40, 127.2, 120.1, 120.0, 116.05, 116.03, 82.2, 74.53, 74.47, 74.4, 74.3, 74.2, 74.1, 73.5, 73.3, 72.3, 72.2, 65.1, 65.0, 54.6, 48.6, 19.01, 18.96, 17.33, 17.29, 17.24, 17.19, 17.14, 17.08, 17.0.

Synthesis of iB-PPO-2000

HBA-BA (1.547 g, 6.107 mmol) was added as a solid to a 20 mL scintillation vial and melted at 100 °C. In a dropwise fashion, PPO-2000 (7.124 g, 3.399 mmol) was added to the scintillation vial, and the mixture was stirred at 100 °C for 1 h. Then, the temperature was increased to 120 °C, and the mixture was allowed to stir for 1 h under vacuum, which resulted in a yellow, viscous liquid upon cooling to room temperature (quantitative yield). ^{1}H NMR (600 MHz, DMSO-d_6) δ 8.25 – 8.05 (m, 2H), 7.77 – 7.03 (m, 15H), 7.03 – 6.48 (m, 2H), 4.90 (s, 2H), 4.05 – 2.94 (m, 163H), 1.26 – 0.70 (m, 109H). ^{13}C NMR (151 MHz, DMSO-d_6) δ 159.0, 138.1, 128.8, 128.5, 128.3, 127.5, 127.4, 127.2, 116.05, 116.02, 82.2, 75.1, 74.6, 74.5, 74.4, 74.3, 74.2, 73.5, 73.3, 72.4, 72.24, 72.19, 72.0, 65.1, 65.0, 54.6, 48.6, 19.01, 18.96, 18.3, 18.1, 17.9, 17.3, 17.21, 17.16, 17.1, 17.0.

Synthesis of iB-PE-116

HBA-BA (4.226 g, 16.68 mmol) was added as a solid to a 20 mL scintillation vial and melted at 100 °C. In a dropwise fashion, PE-116 (1.072 g, 9.225 mmol) was added to the scintillation vial, and the mixture was stirred at 100 °C for 1 h. Then, the temperature was increased to 120 °C, and the mixture was allowed to stir for 1 h under vacuum, which resulted in a pale yellow, tacky amorphous solid upon cooling to room temperature (quantitative yield, T_g = 8.56 °C). ^{1}H NMR (600 MHz, Chloroform-*d*) δ 8.22 – 7.98 (m, 2H), 7.62 – 6.61 (m, 19H), 4.77 (s, 4H), 4.14 – 3.41 (m, 14H), 1.68 (h, *J* = 7.2 Hz, 4H), 1.38 (h, *J* = 9.6, 6.8 Hz, 6H). ^{13}C NMR (151 MHz, Chloroform-*d*) δ 160.1, 156.3, 137.9, 129.2, 129.0, 128.8, 128.7, 128.6, 128.5, 128.4, 128.2, 128.0, 127.5, 127.1, 120.1, 116.5, 82.6, 65.0, 61.6, 55.6, 49.5.

Synthesis of iB-PE-144

HBA-BA (3.764 g, 14.86 mmol) was added as a solid to a 20 mL scintillation vial and melted at 100 °C. In a dropwise fashion, PE-144 (1.176 g, 8.152 mmol) was added to the scintillation vial, and the mixture was stirred at 100 °C for 1 h. Then, the temperature was increased to 120 °C, and the mixture was allowed to stir for 1 h under vacuum, which resulted in a pale yellow, tacky amorphous solid upon cooling to room temperature (quantitative yield, T_g = 5.39 °C). ^{1}H NMR (600 MHz, Chloroform-*d*) δ 8.22 – 8.04 (m, 2H), 7.55 – 7.13 (m, 17H), 7.05 – 6.57 (m, 2H), 5.06 – 4.80 (m, 4H), 4.14 – 3.45 (m, 14H), 1.90 – 0.88 (m, 17H). ^{13}C NMR (151 MHz, Chloroform-*d*) δ 161.3, 160.3, 160.12, 160.08, 156.3, 137.9, 129.8, 129.7, 129.2, 129.0, 128.8, 128.7, 128.5, 128.4, 128.2, 128.0, 127.7, 127.6, 127.5, 127.1, 126.92, 126.89, 120.1, 116.5, 116.4, 82.9, 82.7, 82.6, 65.0, 61.7, 55.6, 52.9, 52.7, 51.8, 51.4, 50.1, 49.5, 46.6, 31.1, 31.0, 29.5, 29.44, 29.40, 28.1, 27.5, 27.3, 27.2.

Synthesis of iB-PE-172

HBA-BA (3.141 g, 12.40 mmol) was added as a solid to a 20 mL scintillation vial and melted at 100 °C. In a dropwise fashion, PE-172 (1.178 g, 6.836 mmol) was added to the scintillation vial, and the mixture was stirred at 100 °C for 1 h. Then, the temperature was increased to 120 °C, and the mixture was allowed to stir for 1 h under vacuum, which resulted in a pale yellow, tacky amorphous solid upon cooling to room temperature (quantitative yield, T_g = -2.21 °C). ^{1}H NMR (600 MHz, Chloroform-*d*) δ 8.22 – 7.95 (m, 2H), 7.62 – 7.10 (m, 17H), 7.01 – 6.62 (m, 2H), 4.90 (s, 4H), 4.16 – 3.40 (m, 13H), 1.81 – 1.01 (m, 20H). ^{13}C NMR (151 MHz, Chloroform-*d*) δ 161.3, 160.3, 160.1, 156.3, 137.9, 129.2, 129.0, 128.8, 128.7, 128.5, 128.4, 128.2, 128.0, 127.7, 127.6, 127.5, 127.1, 126.9, 120.1, 116.5, 82.7, 82.6, 65.0, 61.7, 55.6, 52.9, 52.7, 51.8, 51.4, 50.1, 49.5, 31.0, 29.6, 29.5, 28.1, 27.4, 27.2.

Synthesis of iB-PEO-104

HBA-BA (3.07 g, 12.12 mmol) was added as a solid to a 20 mL scintillation vial and melted at 100 °C. In a dropwise fashion, PEO-104 (0.695 g, 6.673 mmol) was added to the scintillation vial, and the mixture was stirred at 100 °C for 1 h. Then, the temperature was increased to 120 °C, and the mixture was allowed to stir for 1 h under vacuum, which resulted in a pale yellow,

tacky amorphous solid upon cooling to room temperature (quantitative yield, T_g = 17.38 °C). ^{1}H NMR (600 MHz, DMSO-d_6) δ 8.26 – 7.98 (m, 2H), 7.75 – 7.00 (m, 19H), 7.00 – 6.58 (m, 2H), 4.96 – 4.74 (m, 3H), 4.03 – 3.76 (m, 7H), 3.76 – 3.44 (m, 16H). ^{13}C NMR (151 MHz, DMSO-d_6) δ 161.1, 160.9, 155.9, 138.1, 128.7, 128.5, 128.3, 128.1, 127.8, 127.7, 127.6, 127.4, 127.3, 127.2, 126.7, 120.1, 116.1, 82.3, 69.9, 63.9, 60.0, 54.6, 50.4, 49.5, 48.6.

Synthesis of iB-PEO-220

HBA-BA (4.7270 g, 18.66 mmol) was added as a solid to a 20 mL scintillation vial and melted at 100 °C. In a dropwise fashion, PEO-220 (2.254 g, 10.23 mmol) was added to the scintillation vial, and the mixture was stirred at 100 °C for 1 h. Then, the temperature was increased to 120 °C, and the mixture was allowed to stir for 1 h under vacuum, which resulted in a pale yellow, tacky amorphous solid upon cooling to room temperature (quantitative yield, T_g = -7.72 °C). ^{1}H NMR (600 MHz, DMSO-d_6) δ 8.15 (d, J = 5.8 Hz, 2H), 7.64 – 7.08 (m, 17H), 6.90 – 6.61 (m, 2H), 4.97 – 4.75 (m, 4H), 4.10 – 3.07 (m, 35H), 1.93 – 1.47 (m, 5H). ^{13}C NMR (151 MHz, DMSO-d_6) δ 160.9, 160.1, 155.9, 138.1, 128.7, 128.5, 128.30, 128.26, 128.21, 128.15, 128.1, 127.8, 127.7, 127.5, 127.4, 127.2, 126.8, 126.6, 120.1, 116.1, 115.4, 82.6, 82.3, 82.2, 69.80, 69.76, 69.7, 69.53, 69.51, 68.5, 68.2, 68.10, 68.05, 63.9, 57.1, 54.6, 51.8, 48.6, 47.7, 30.8, 30.7, 27.8.

S2. Spectroscopic and Thermal Analyses of iBOX Monomers

Nuclear Magnetic Resonance (NMR) Spectroscopy of HBA-BA

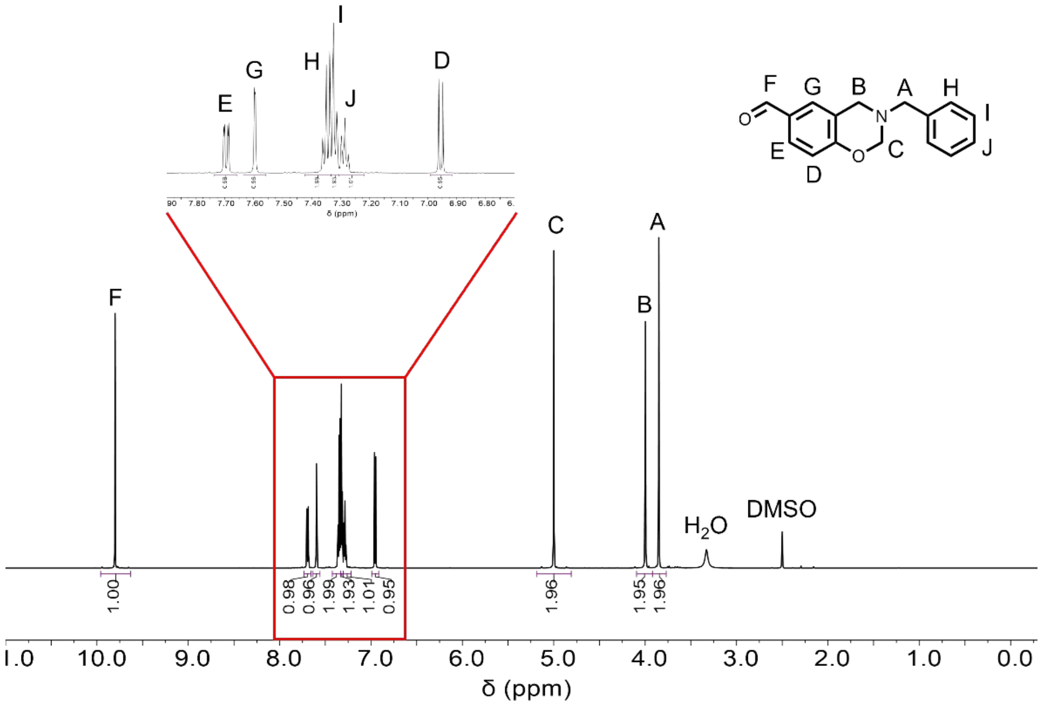

Figure S1. ^{1}H NMR spectrum of HBA-BA.

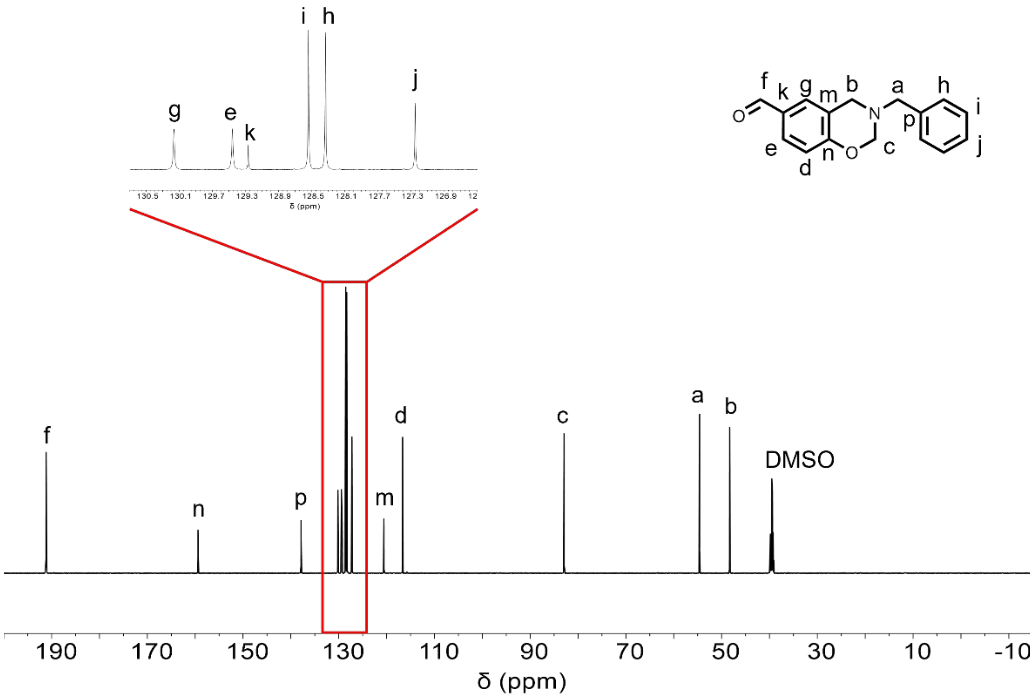

Figure S2. ^{13}C NMR of spectrum of HBA-BA.

NMR Spectroscopy of iBOX Monomers

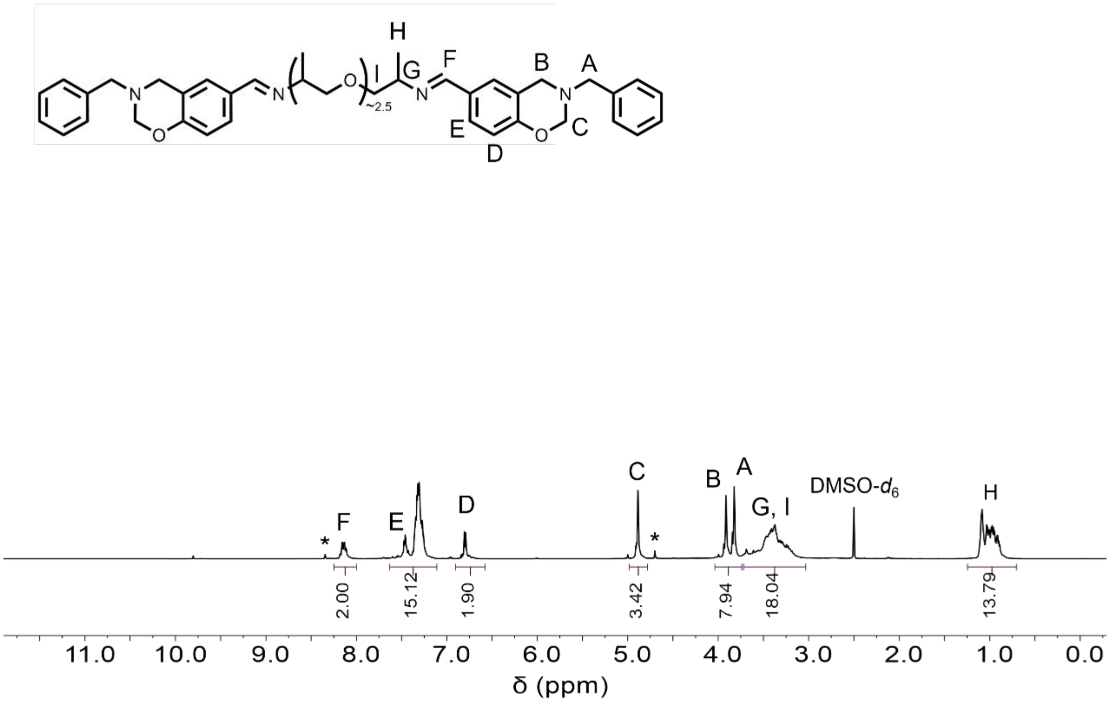

Figure S3. ^{1}H NMR spectrum of iB-PPO-230. Peaks denoted by (*) are associated with ring-opened species.

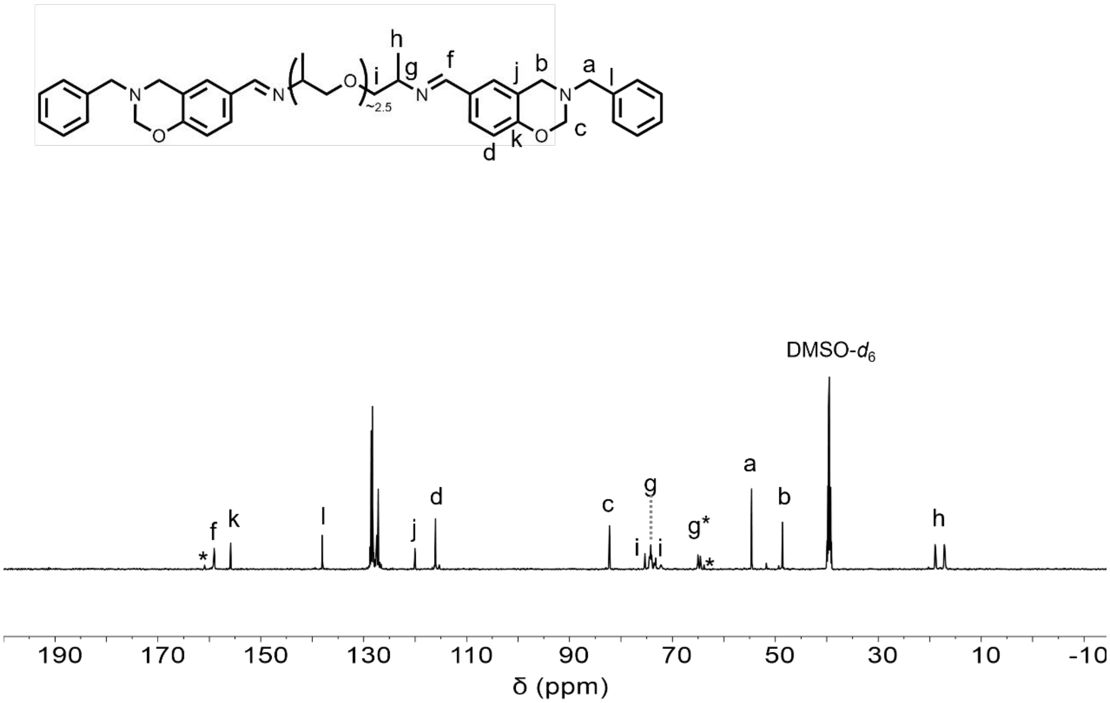

Figure S4. ^{13}C NMR of spectrum of iB-PPO-230.

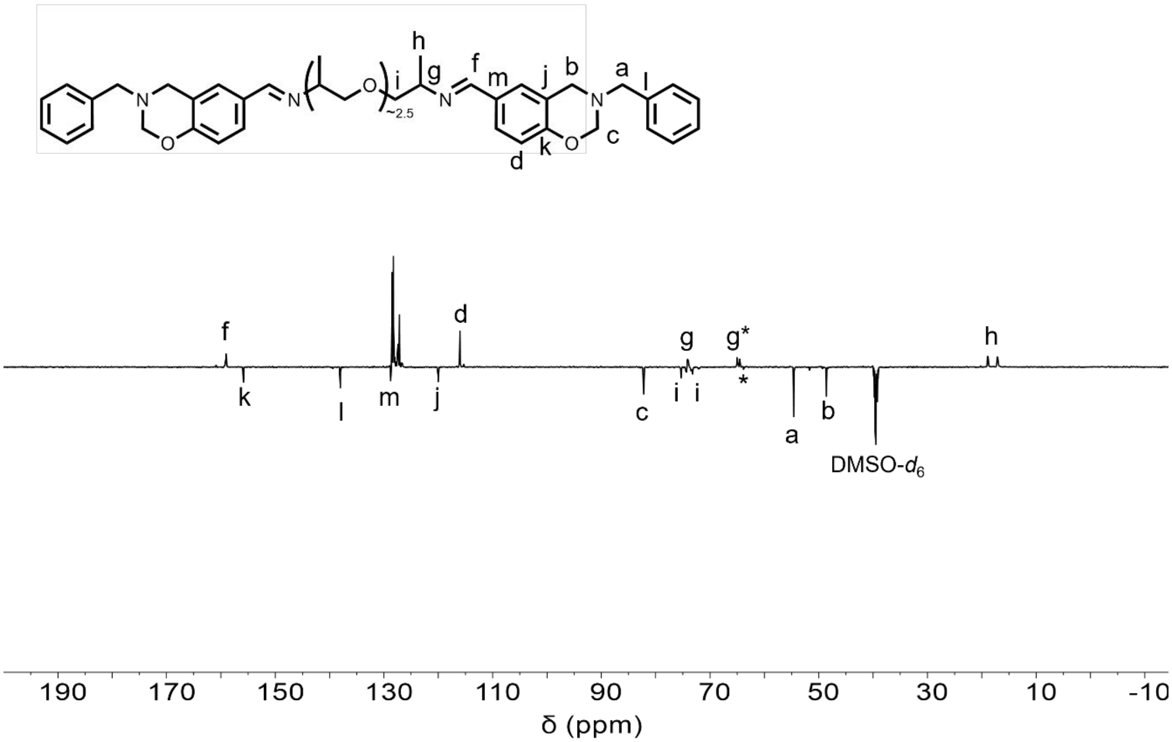

Figure S5. APT NMR spectrum of iB-PPO-230.

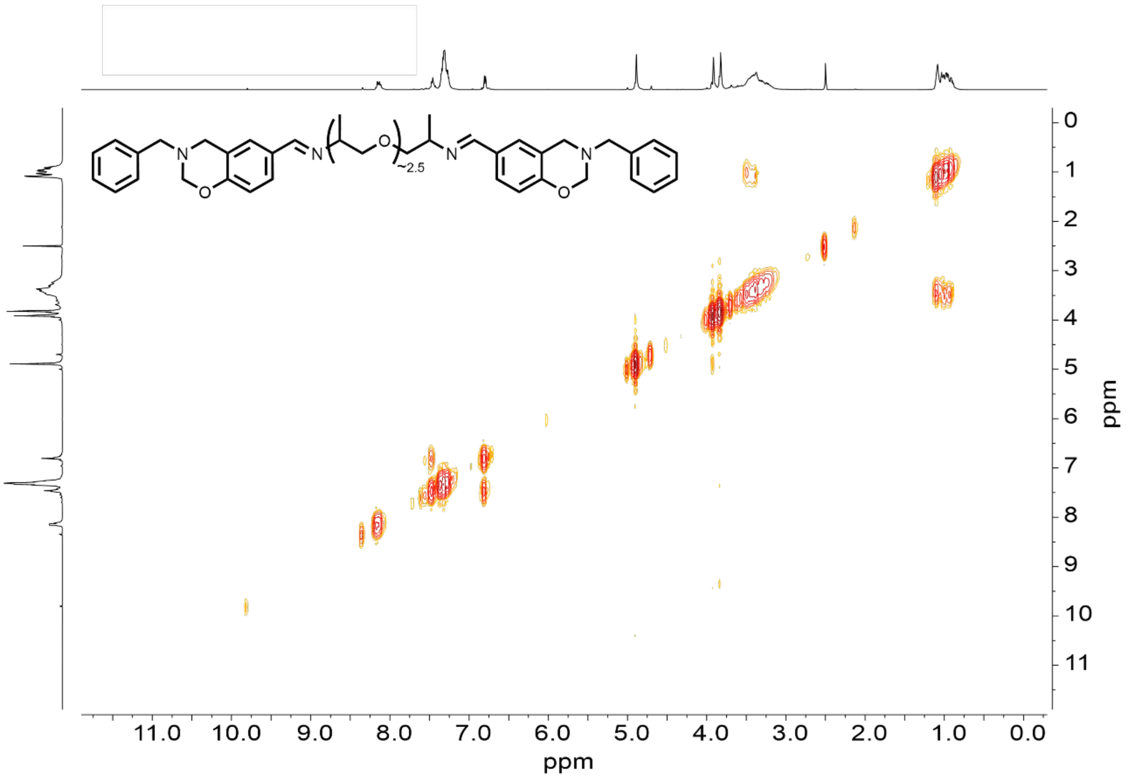

Figure S6. COSY NMR spectrum of iB-PPO-230.

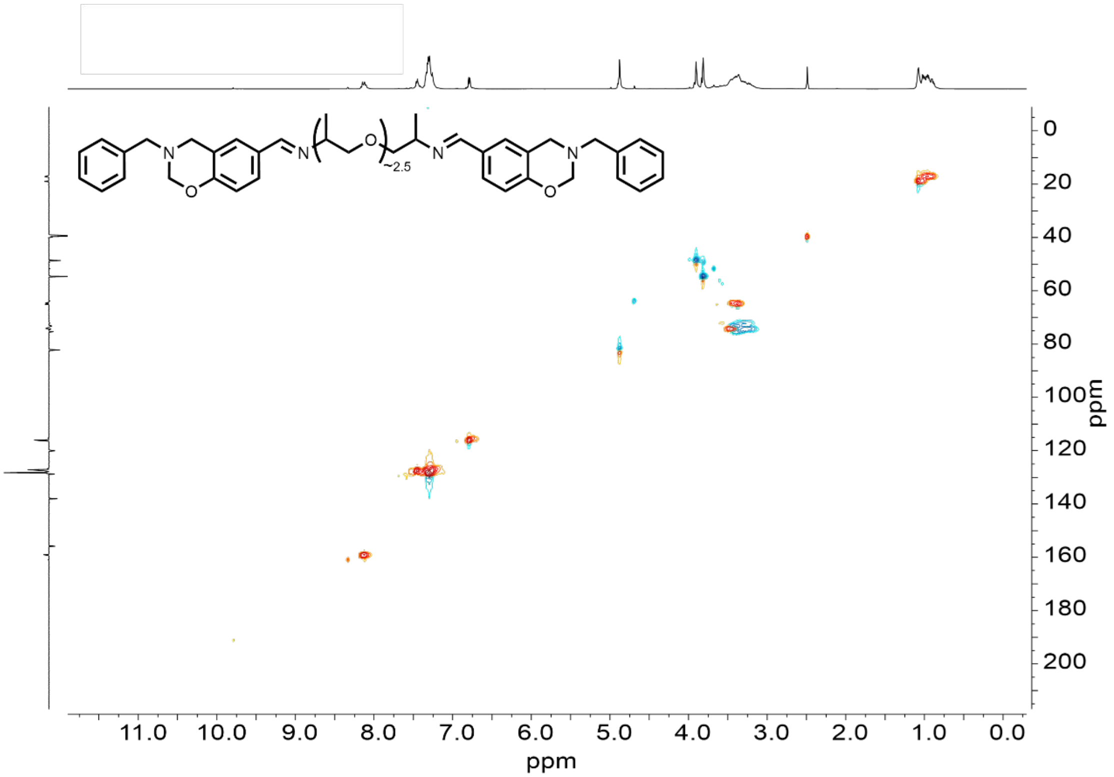

Figure S7. HSQC NMR spectrum of iB-PPO-230.

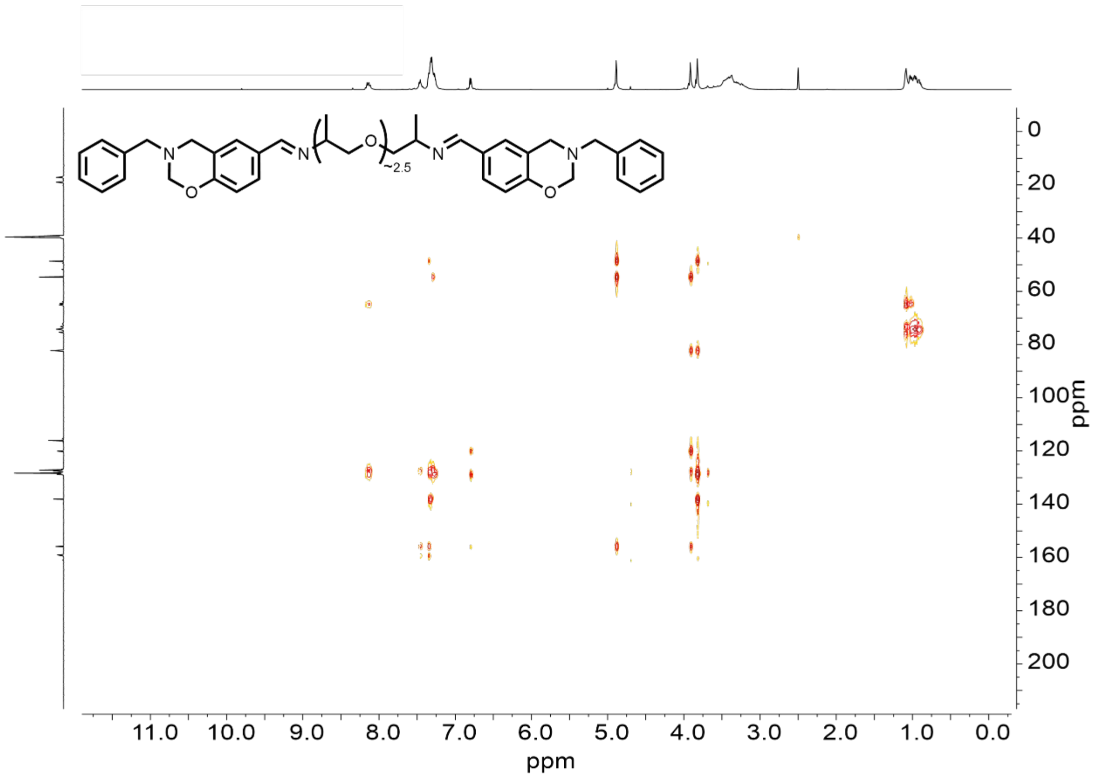

Figure S8. HMBC NMR spectrum of iB-PPO-230.

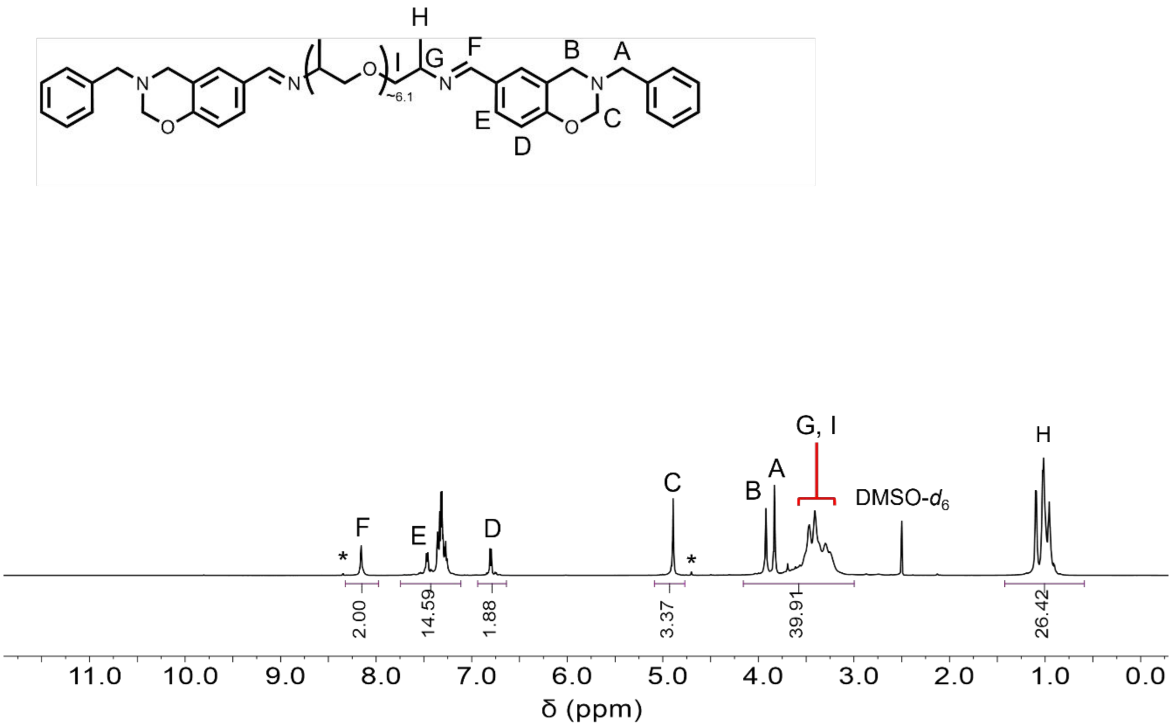

Figure S9. ^{1}H NMR spectrum of iB-PPO-400.

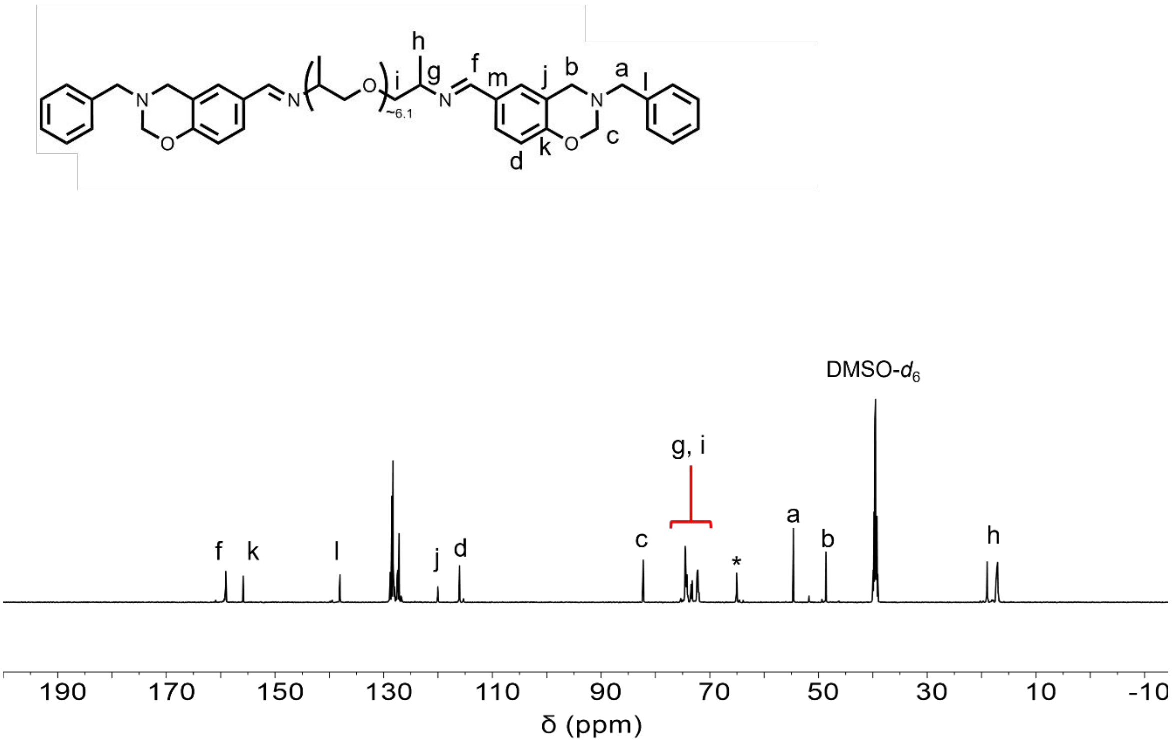

Figure S10. ^{13}C NMR of spectrum of iB-PPO-400.

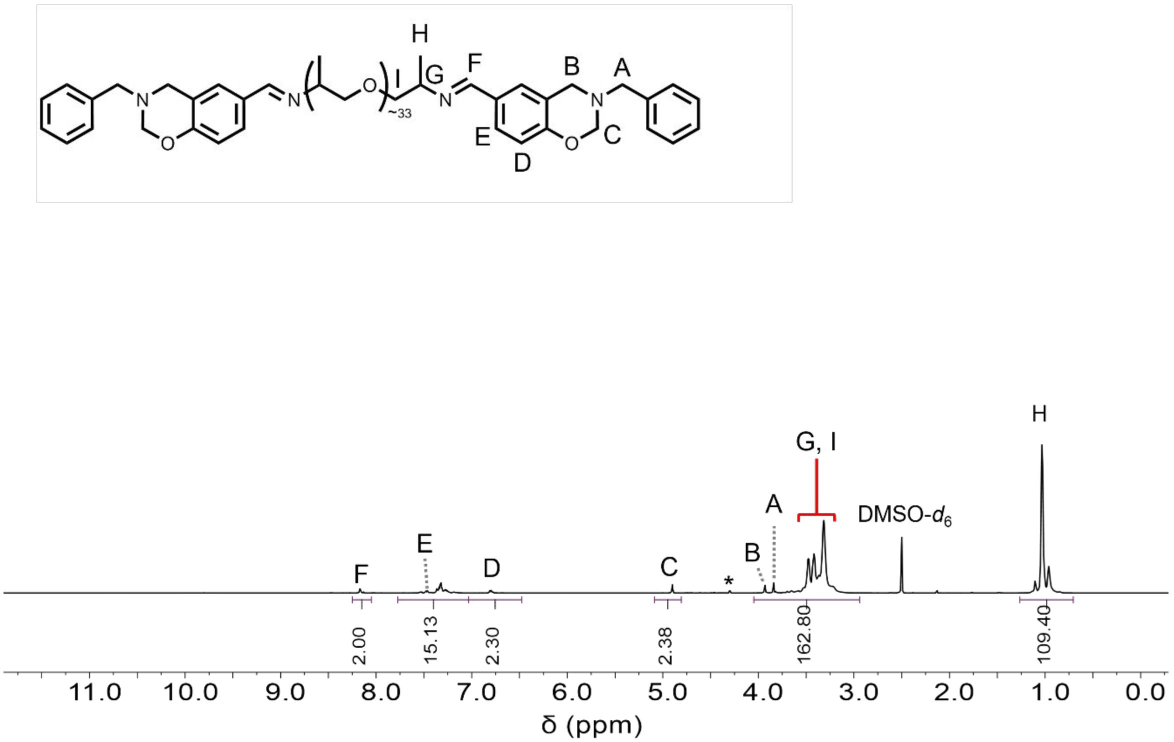

Figure S11. ^{1}H NMR spectrum of iB-PPO-2000.

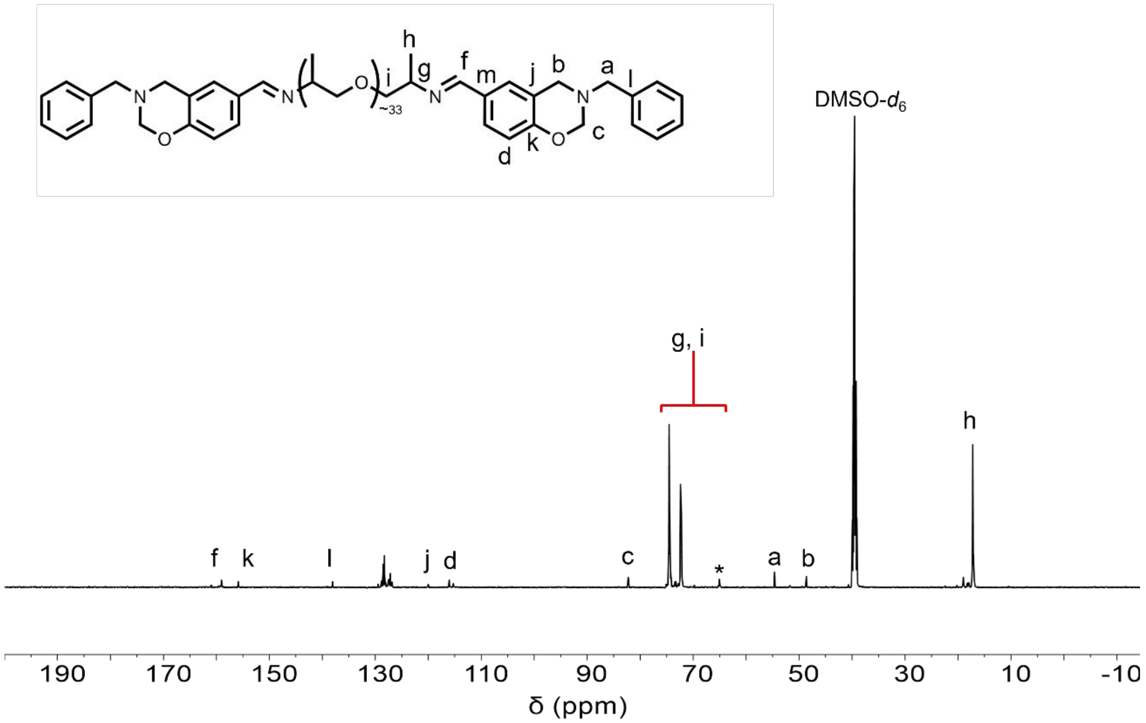

Figure S12. ^{13}C NMR of spectrum of iB-PPO-2000.

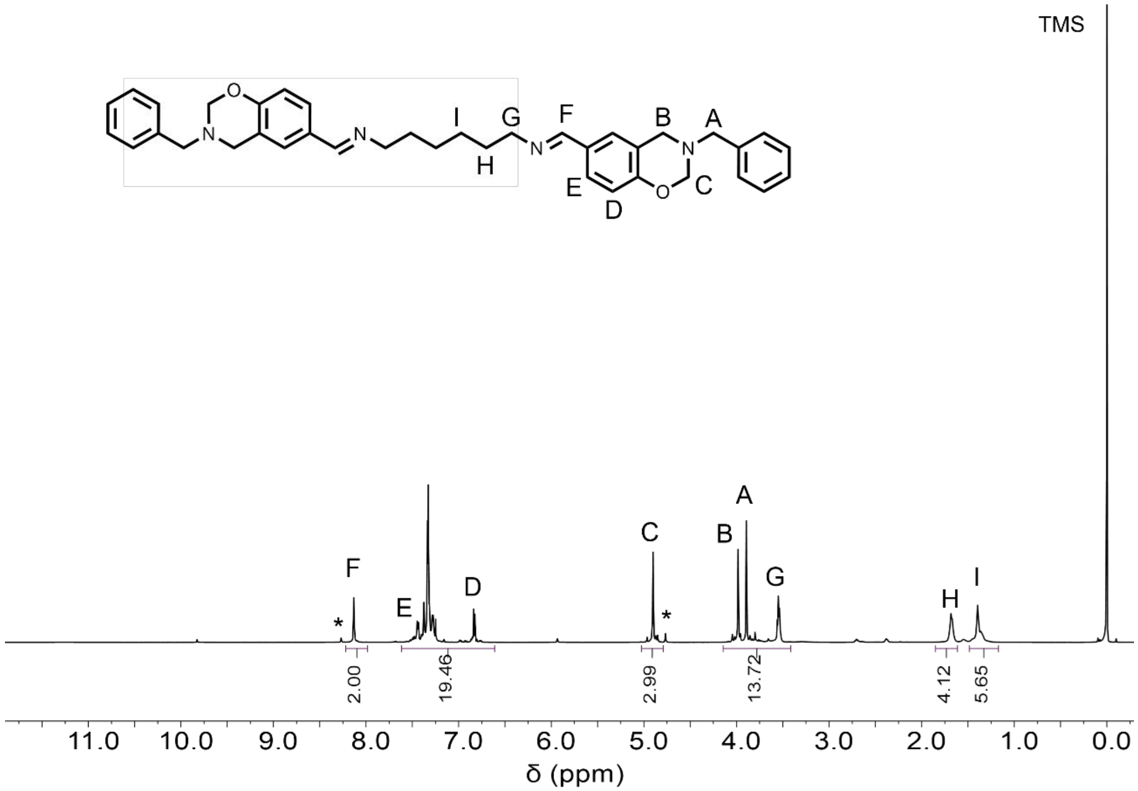

Figure S13. ^{1}H NMR spectrum of iB-PE-116.

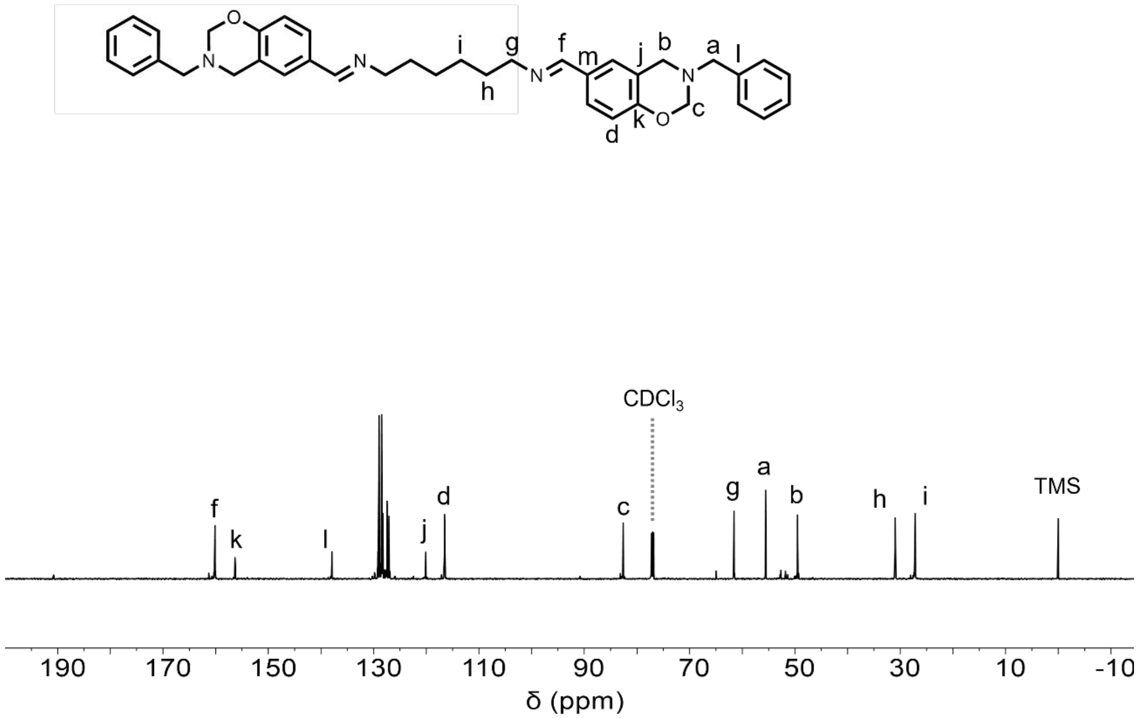

Figure S14. ^{13}C NMR of spectrum of iB-PE-116.

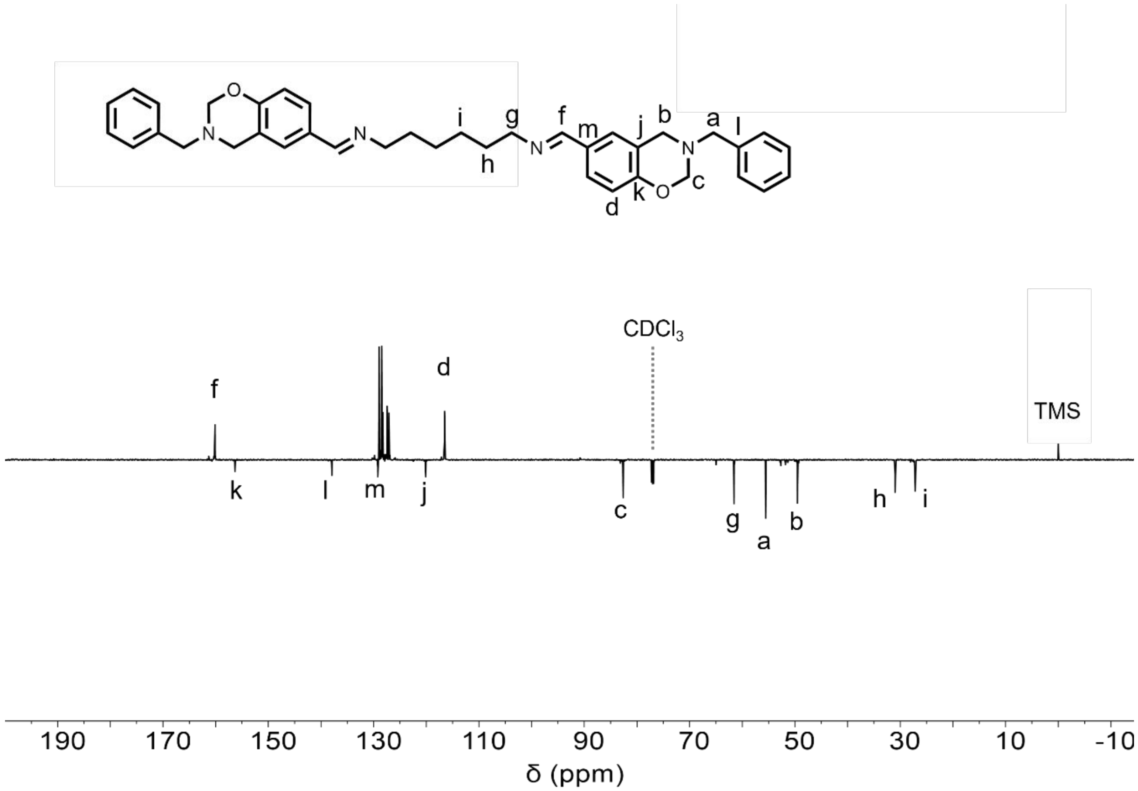

Figure S15. APT NMR spectrum of iB-PE-116.

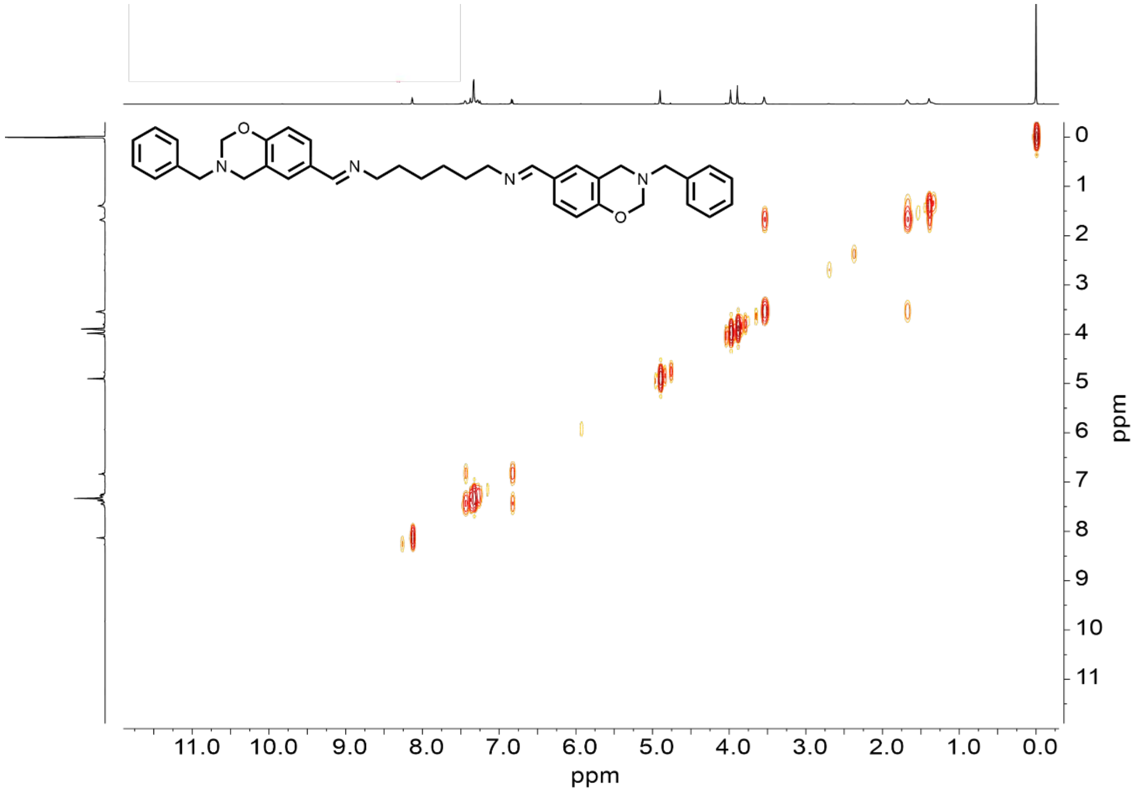

Figure S16. COSY NMR spectrum of iB-PE-116.

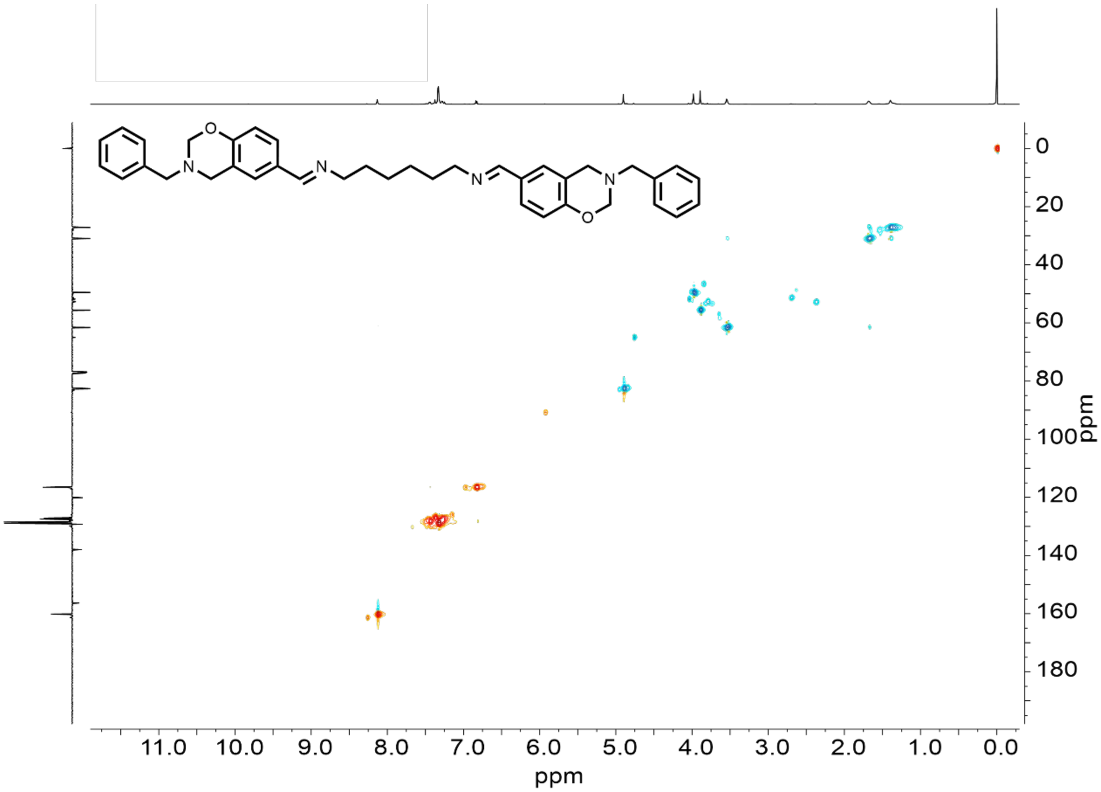

Figure S17. HSQC NMR spectrum of iB-PE-116.

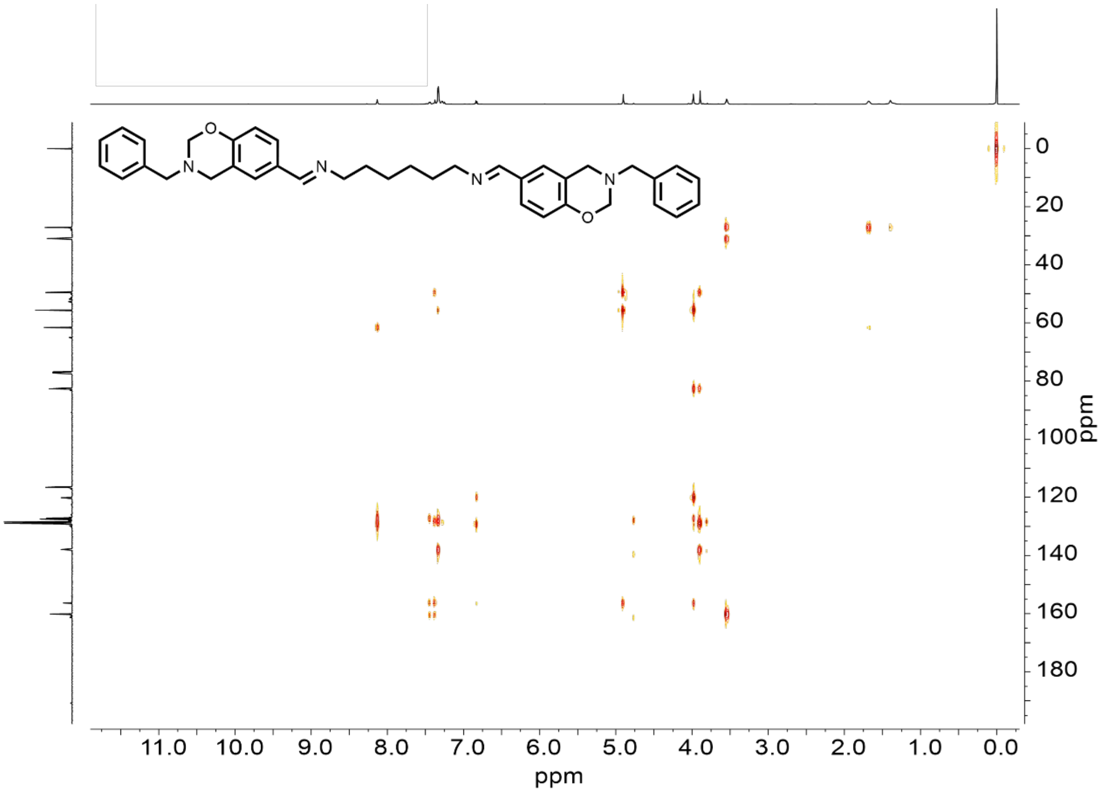

Figure S18. HMBC NMR spectrum of iB-PE-116.

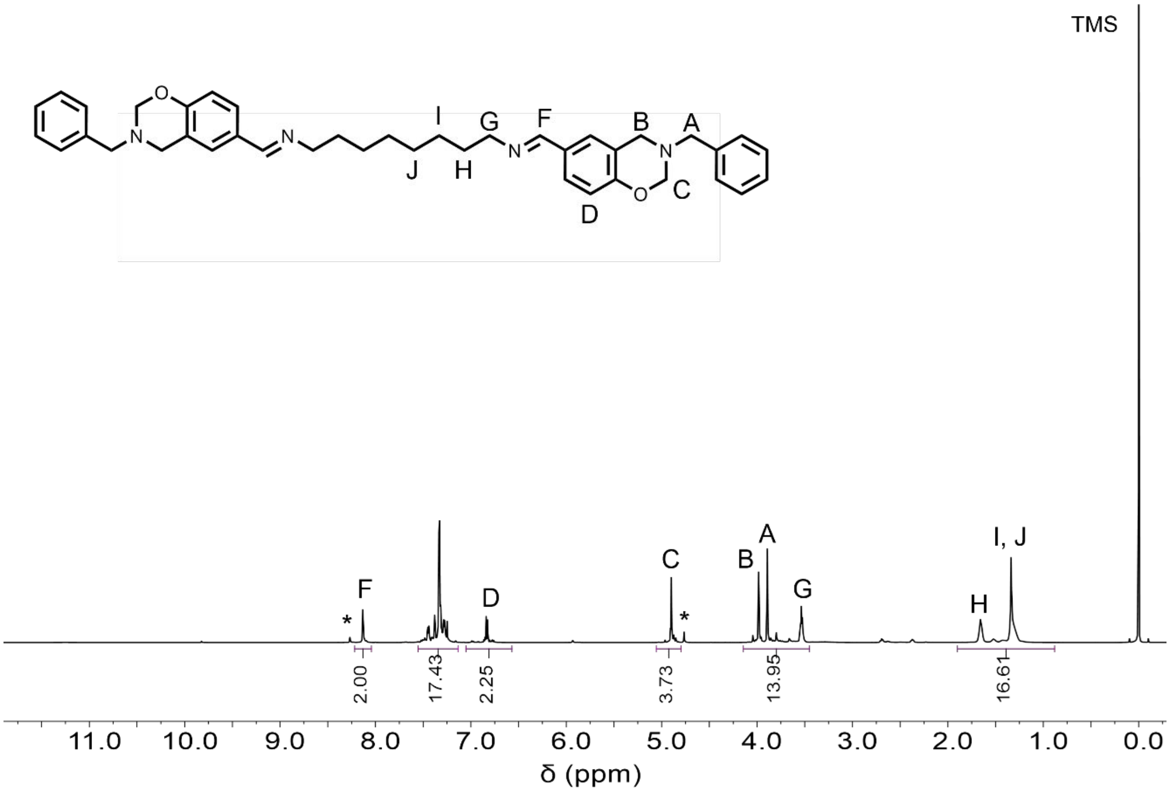

Figure S19. ^{1}H NMR spectrum of iB-PE-144.

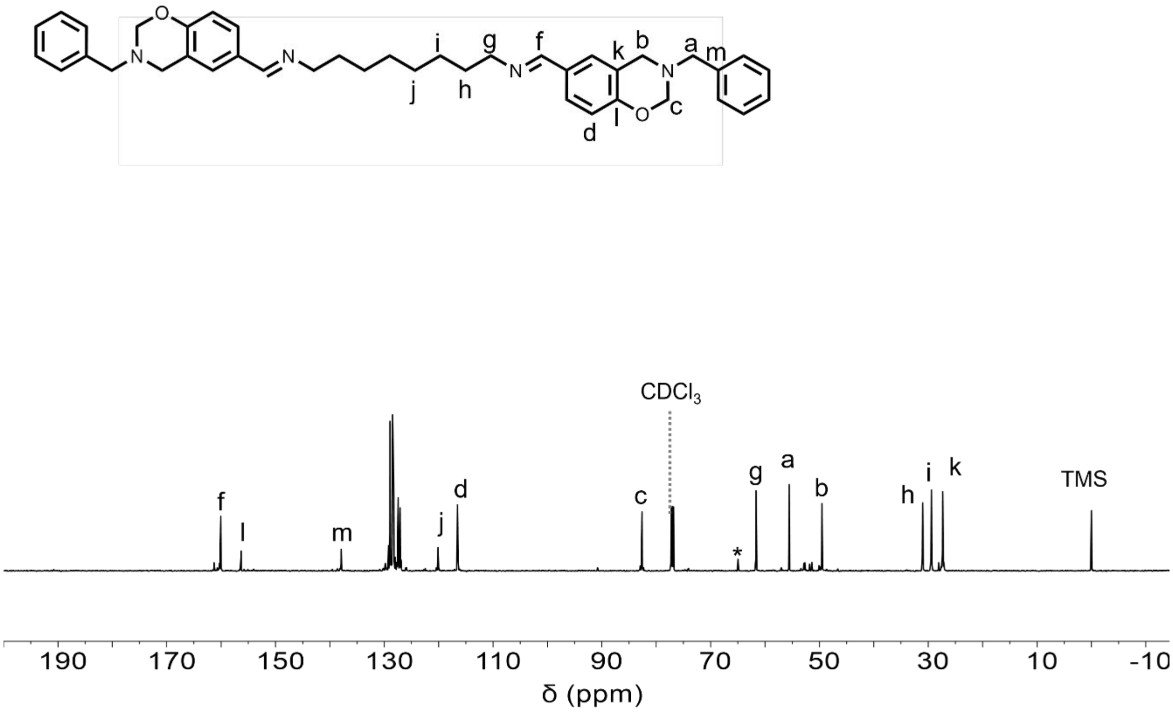

Figure S20. ^{13}C NMR of spectrum of iB-PE-144.

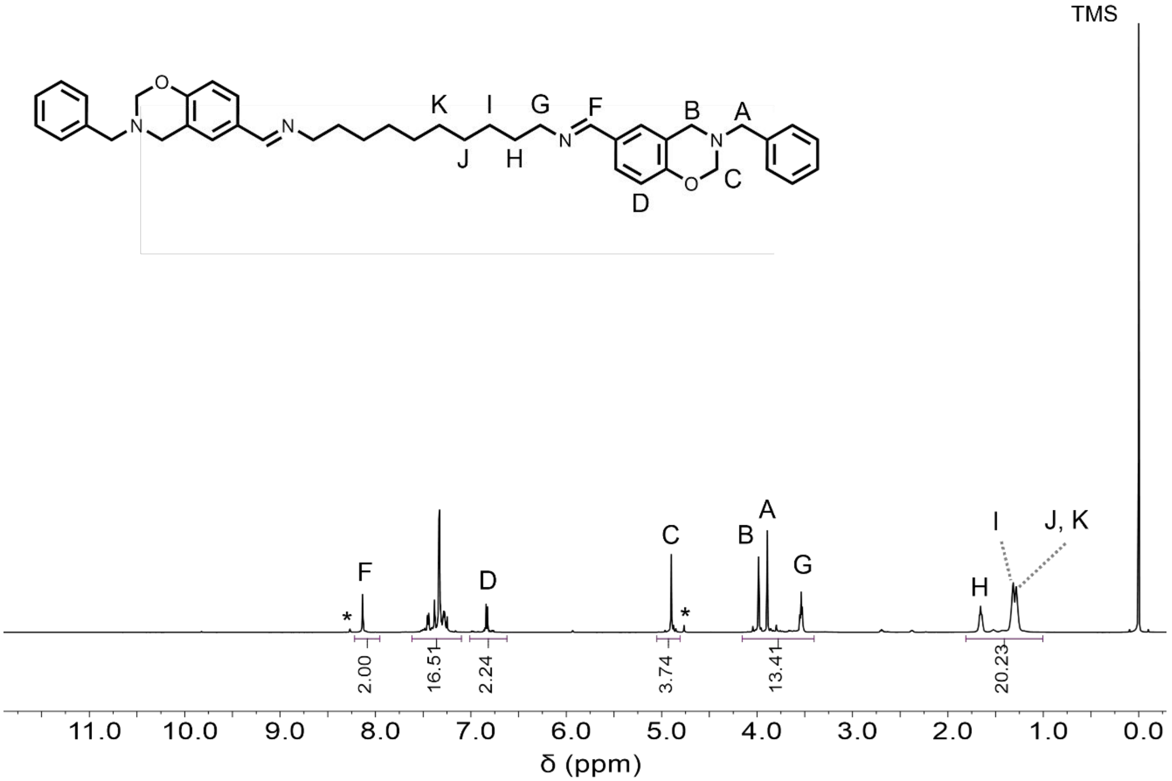

Figure S21. ^{1}H NMR spectrum of iB-PE-172.

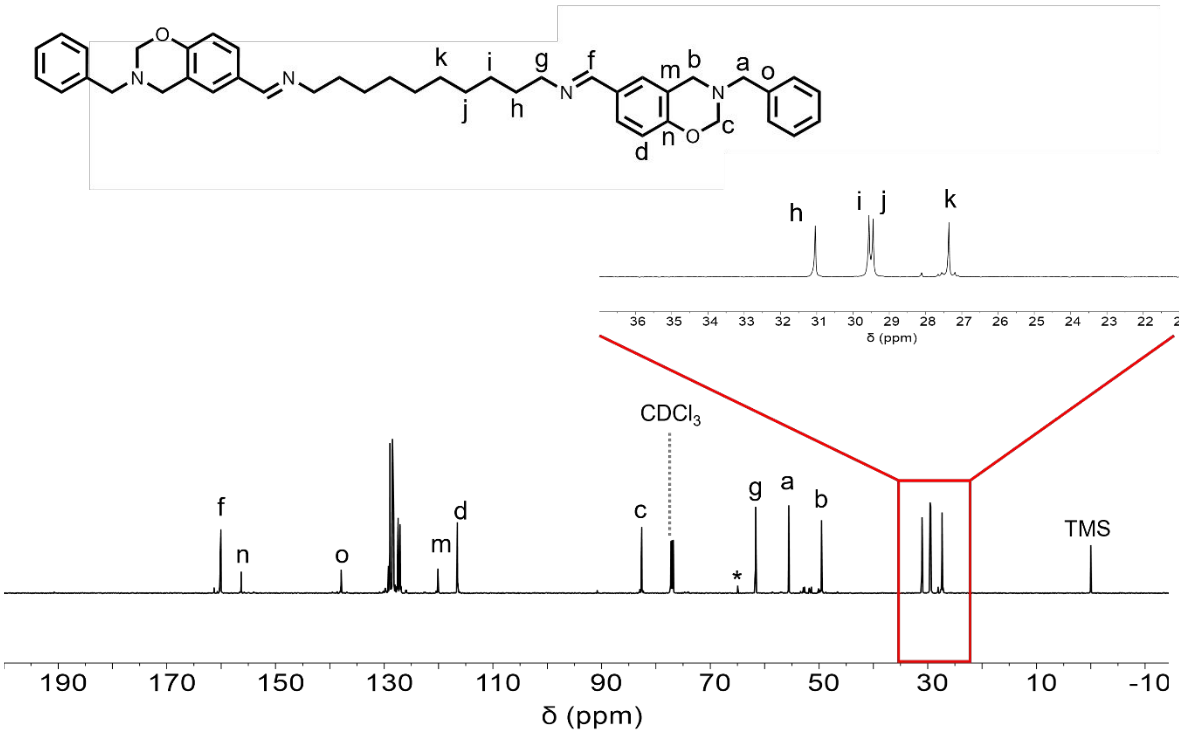

Figure S22. ^{13}C NMR of spectrum of iB- PE-172.

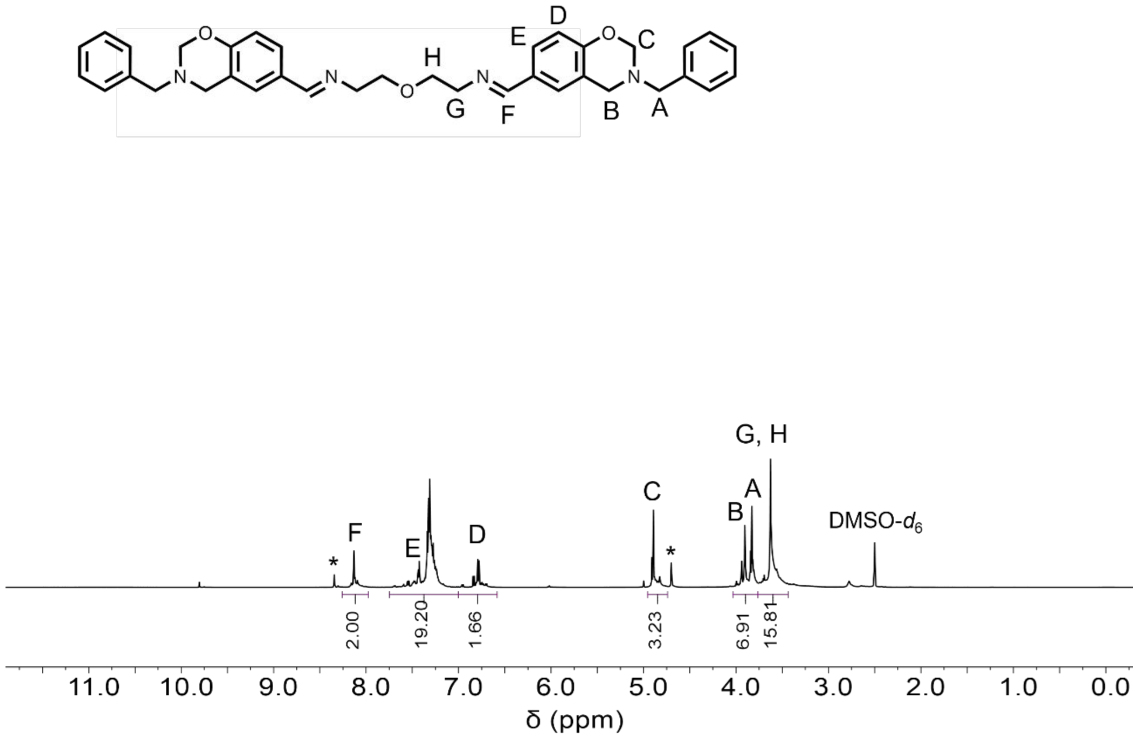

Figure S23. ^{1}H NMR spectrum of iB-PEO-104.

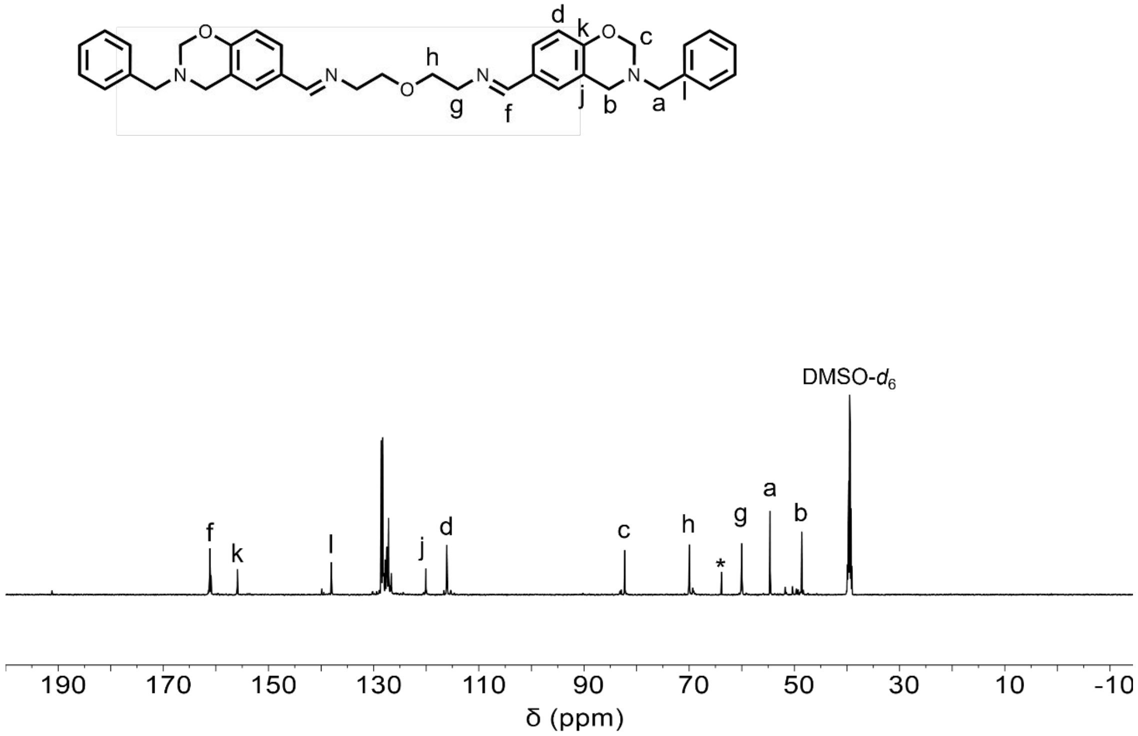

Figure S24. ^{13}C NMR of spectrum of iB-PEO-104.

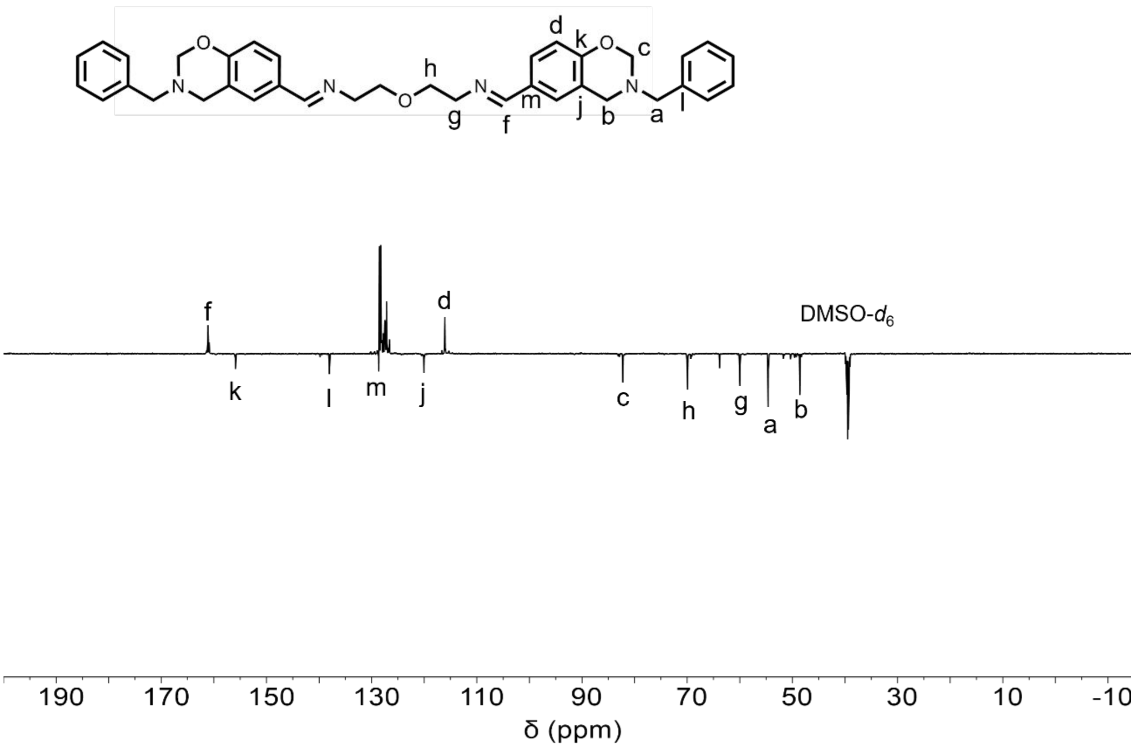

Figure S25. APT NMR spectrum of iB-PEO-104.

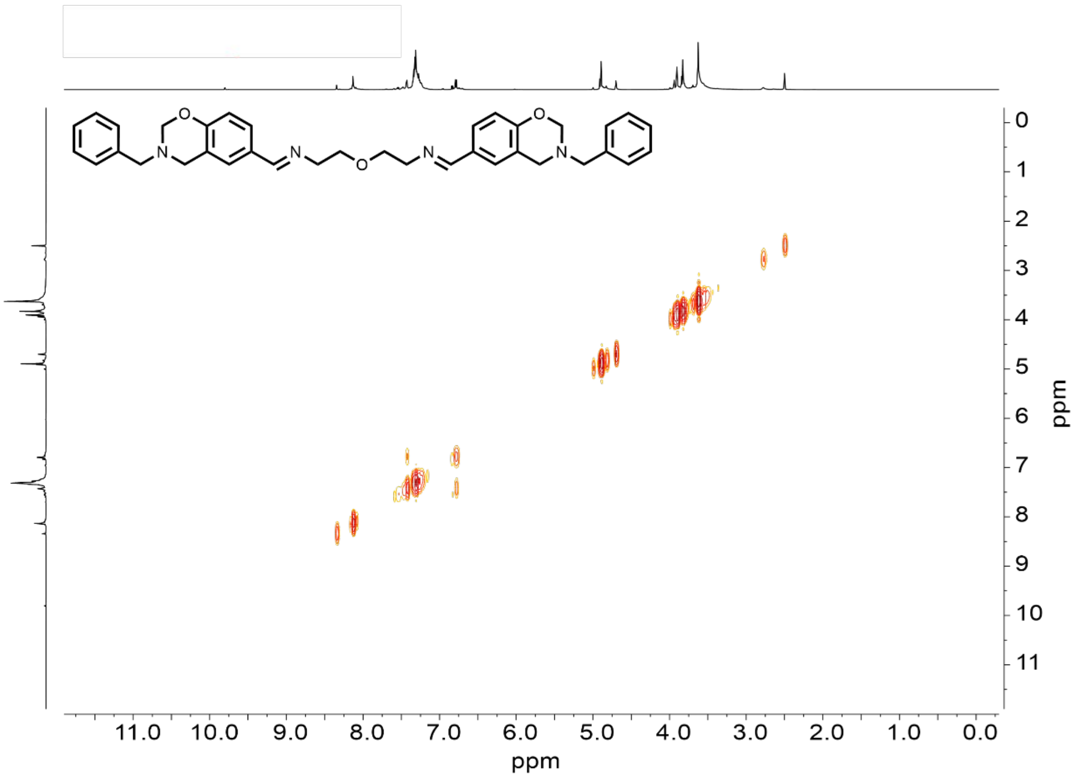

Figure S26. COSY NMR spectrum of iB-PEO-104.

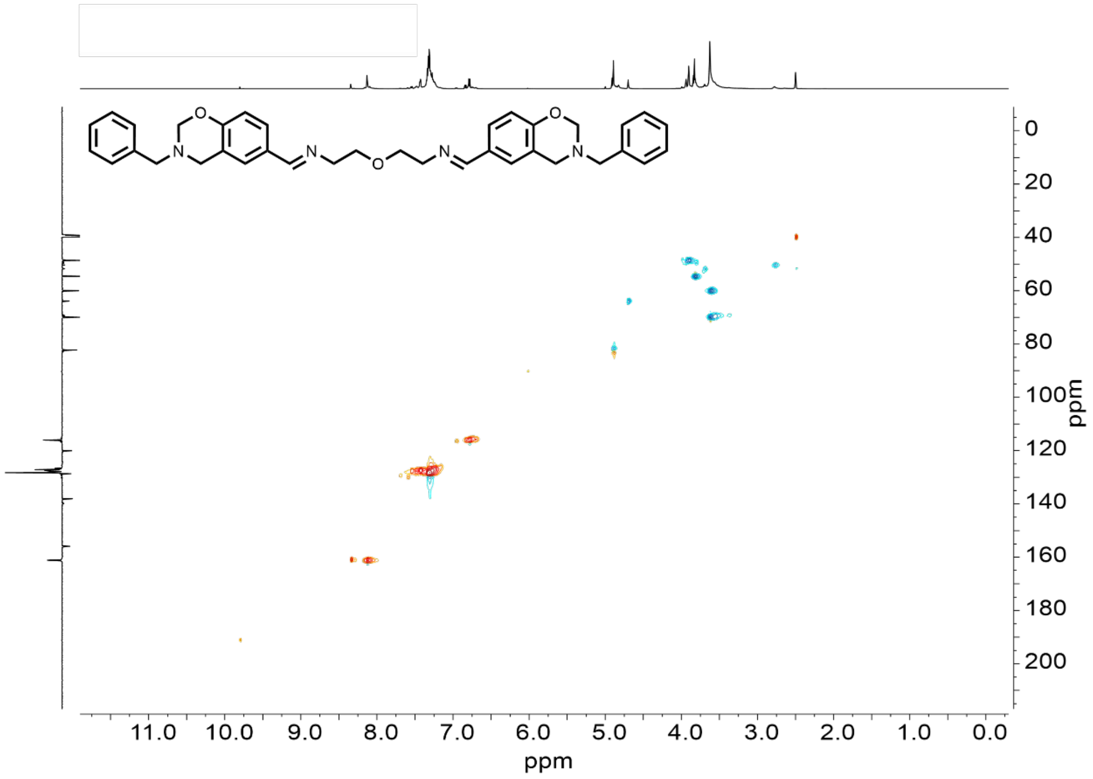

Figure S27. HSQC NMR spectrum of iB-PEO-104.

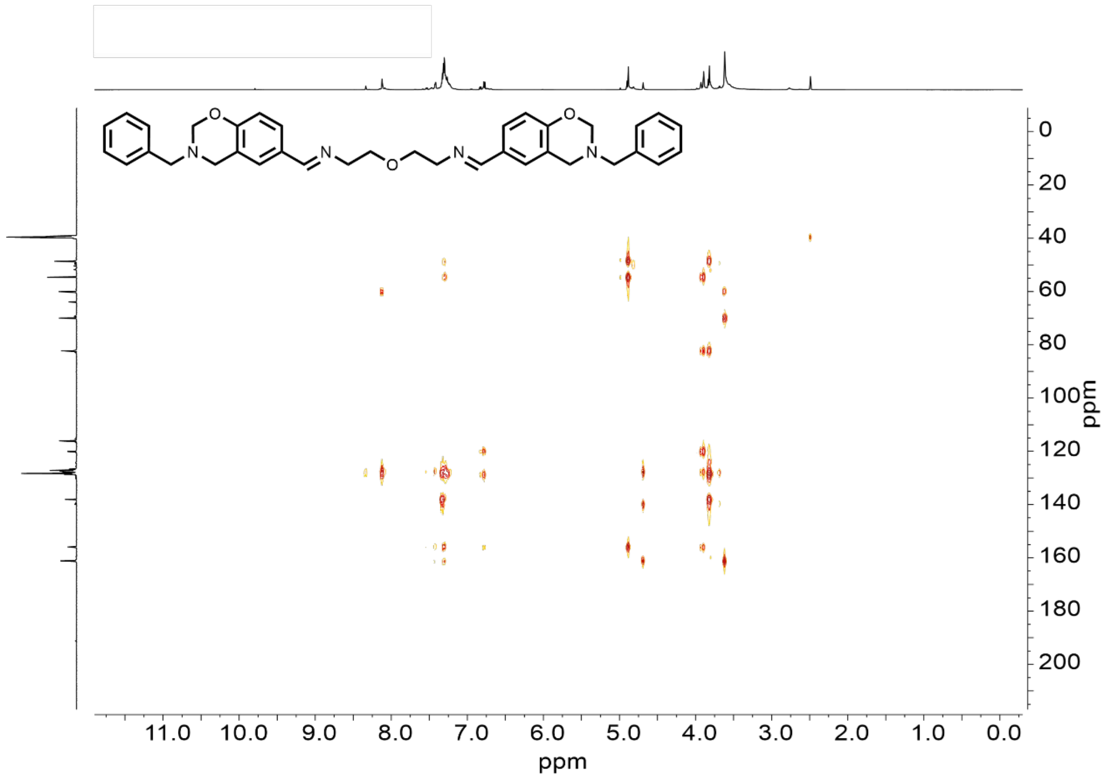

Figure S28. HMBC NMR spectrum of iB-PEO-104.

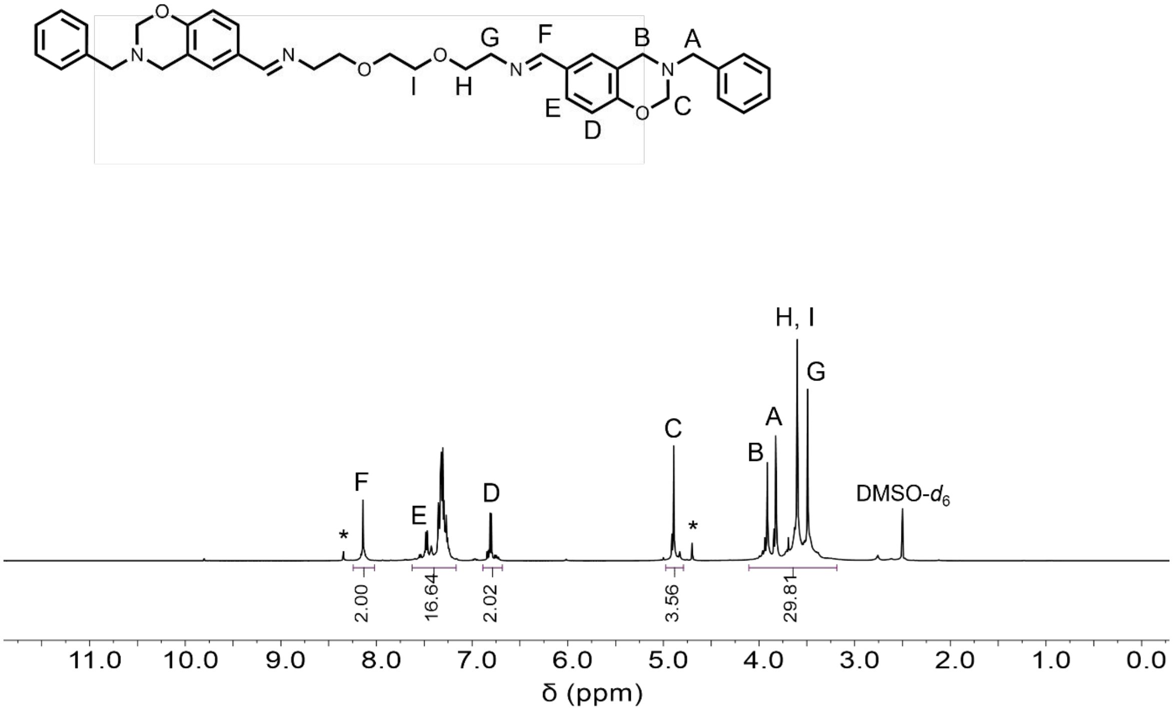

Figure S29. ^{1}H NMR spectrum of iB-PEO-148.

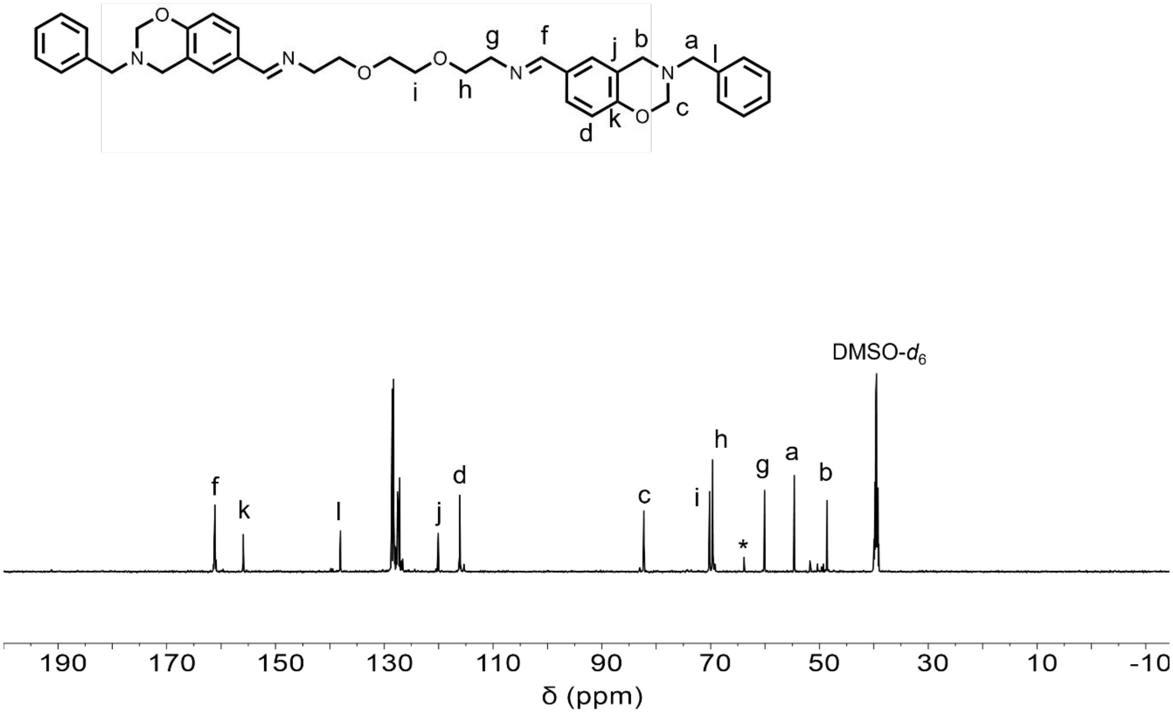

Figure S30. ^{13}C NMR of spectrum of iB-PEO-148.

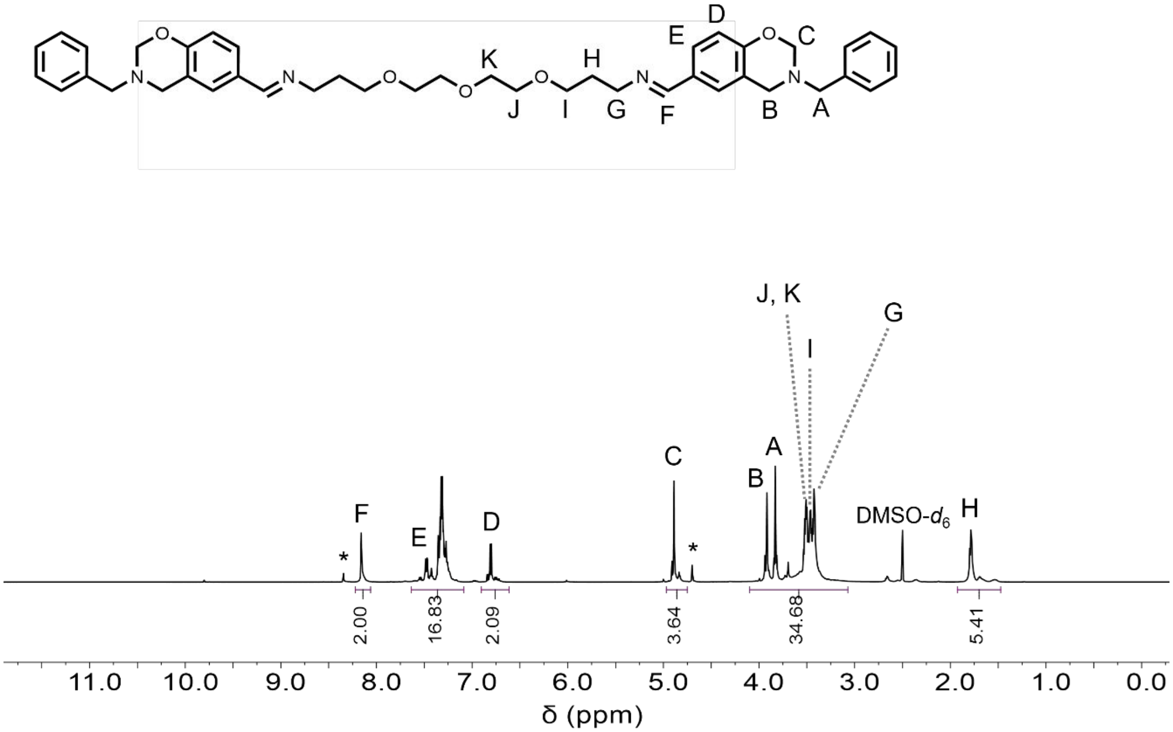

Figure S31. ^{1}H NMR spectrum of iB-PEO-220.

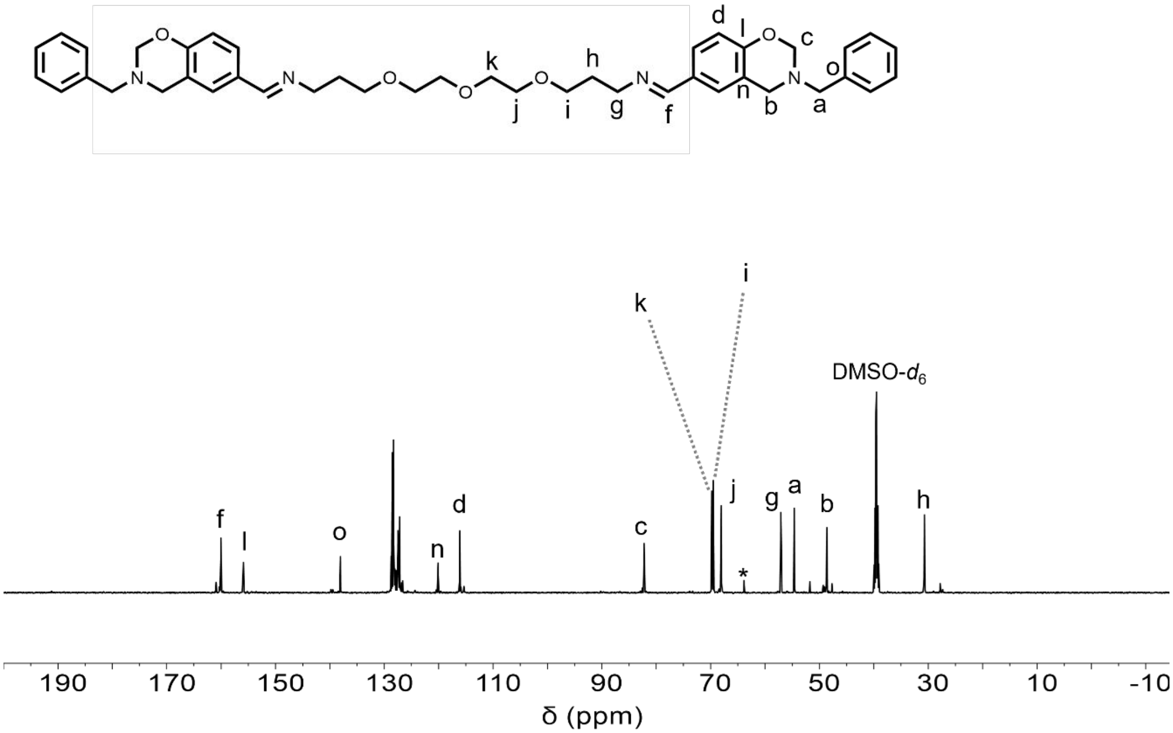

Figure S32. ^{13}C NMR of spectrum of iB-PEO-220.

Attenuated Total Reflectance Fourier-Transform Infrared (ATR-FTIR) of Benzoxazine Monomers

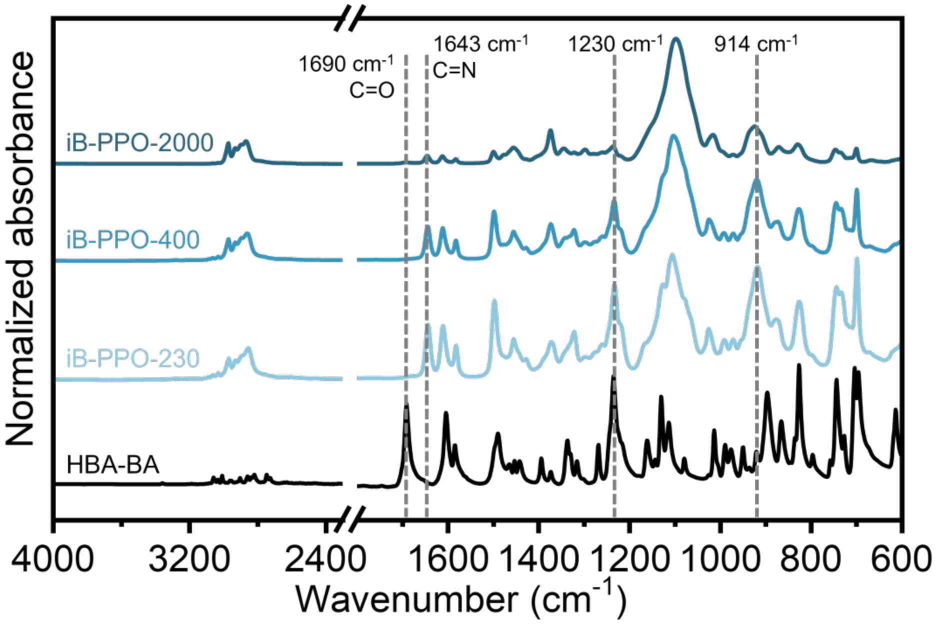

Figure S33. Attenuated total reflectance Fourier-transform infrared (ATR-FTIR) spectra of HBA-BA and iB-PPO-X monomers.

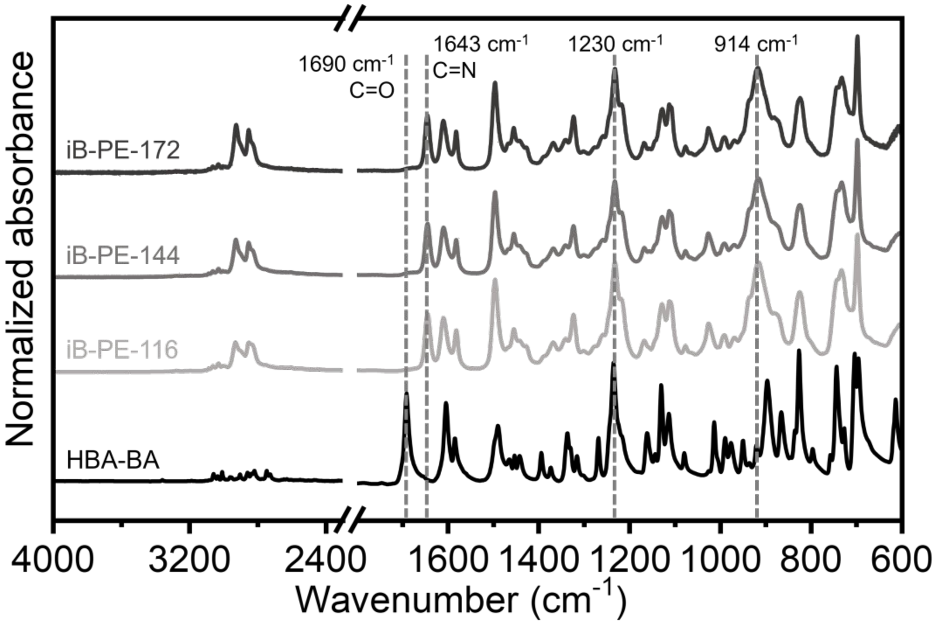

Figure S34. ATR-FTIR spectra of HBA-BA and iB-PE-X monomers.

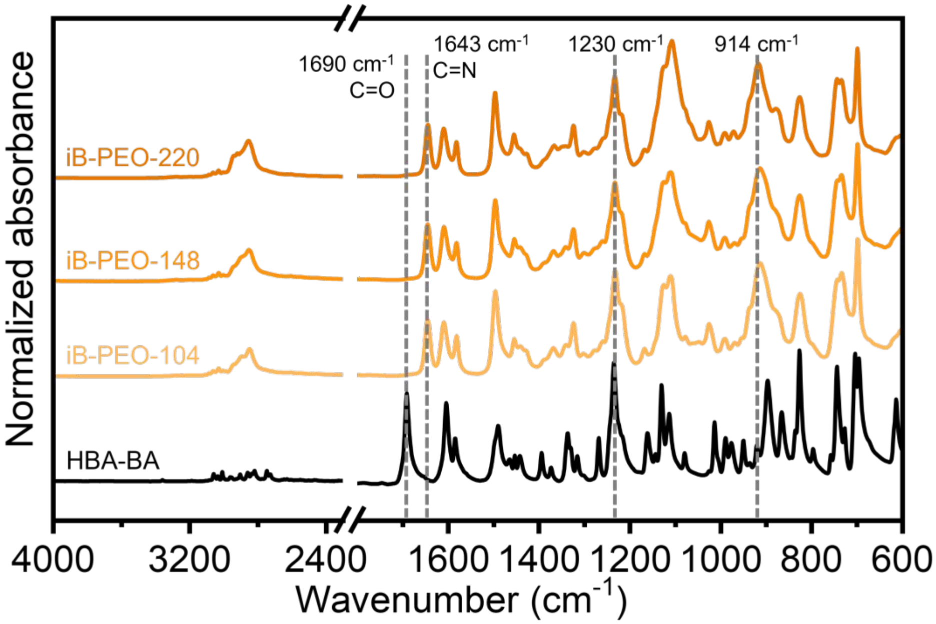

Figure S35. ATR-FTIR spectra of HBA-BA and iB-PEO-X monomers.

Thermogravimetric Analysis and Differential Scanning Calorimetry (DSC) of Benzoxazine Monomers

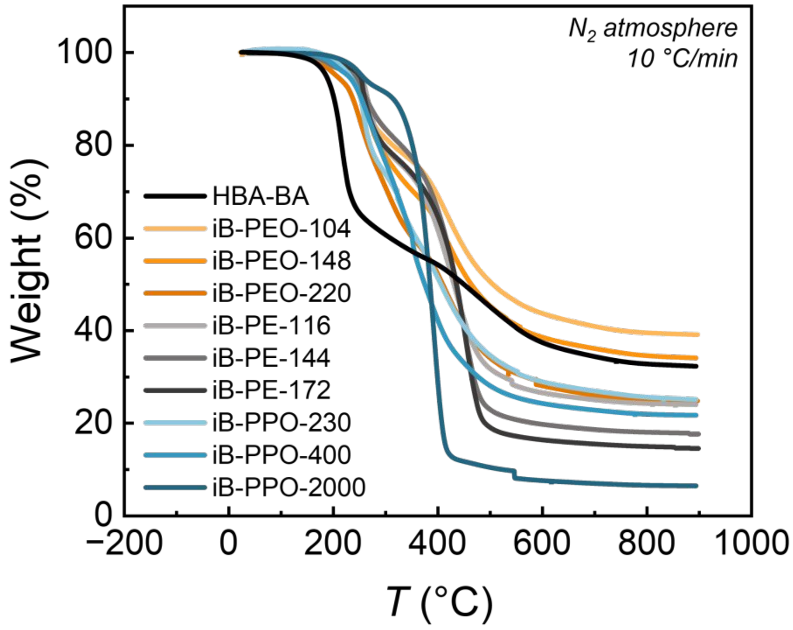

Figure S36. Thermogravimetric analysis of HBA-BA and each iBOX monomer.

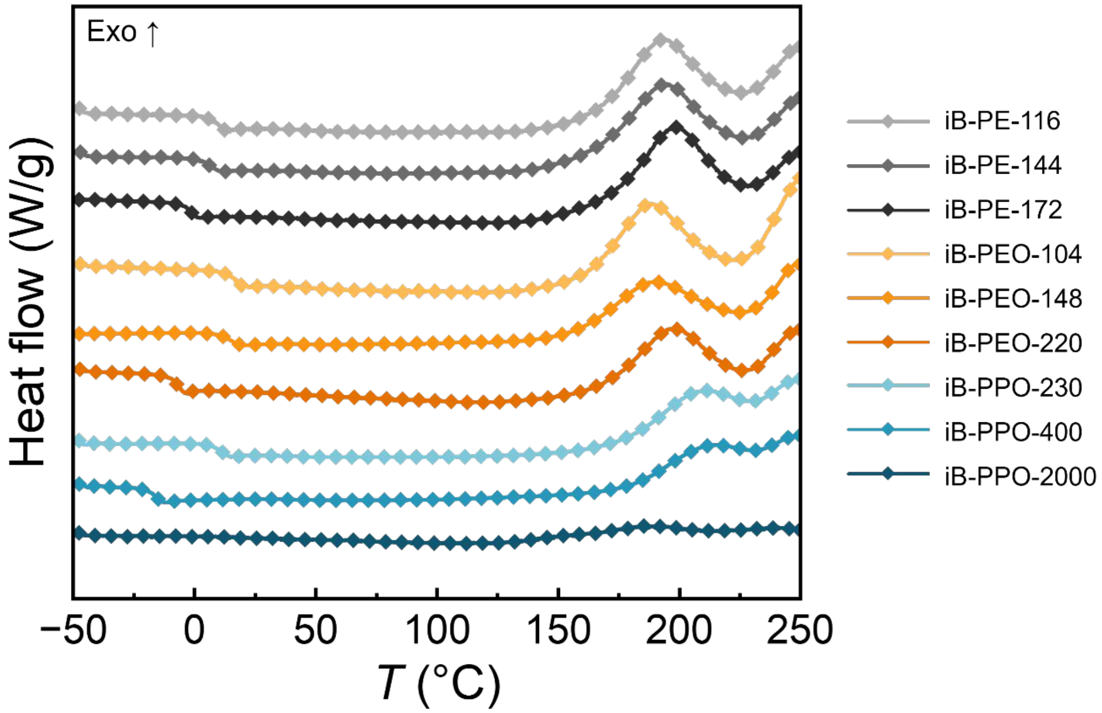

Figure S37. Differential scanning calorimetry of each iBOX monomer.

S3. Spectroscopic and Thermal Analyses of p(iBOX) Networks

ATR-FTIR of p(iBOX) Networks

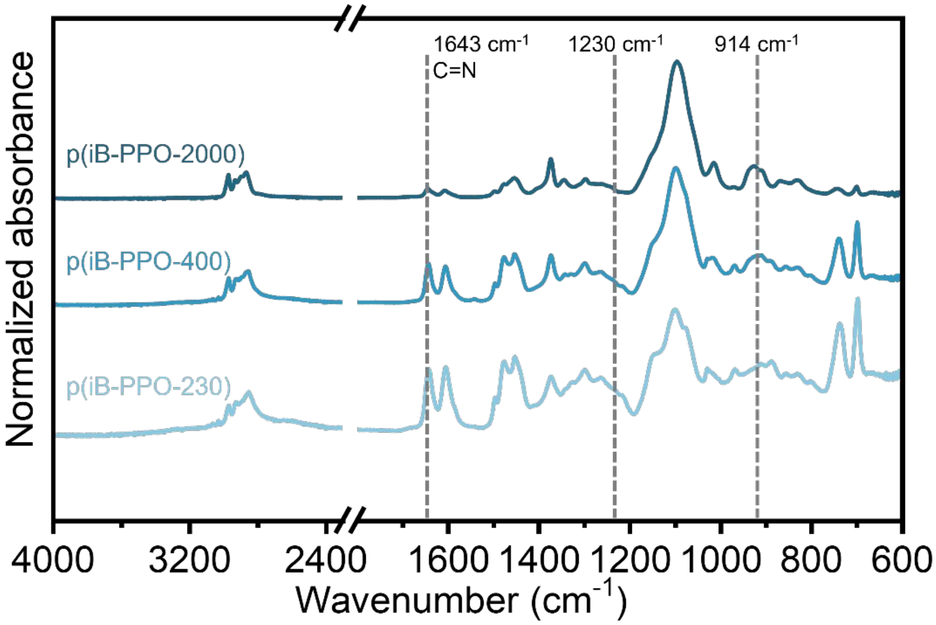

Figure S38 ATR-FTIR spectra of p(iB-PPO-X) networks, cross-linked at 180 °C for 2 h, except for p(iB-PPO-2000), which was cross-linked at 180 °C for 3 h.

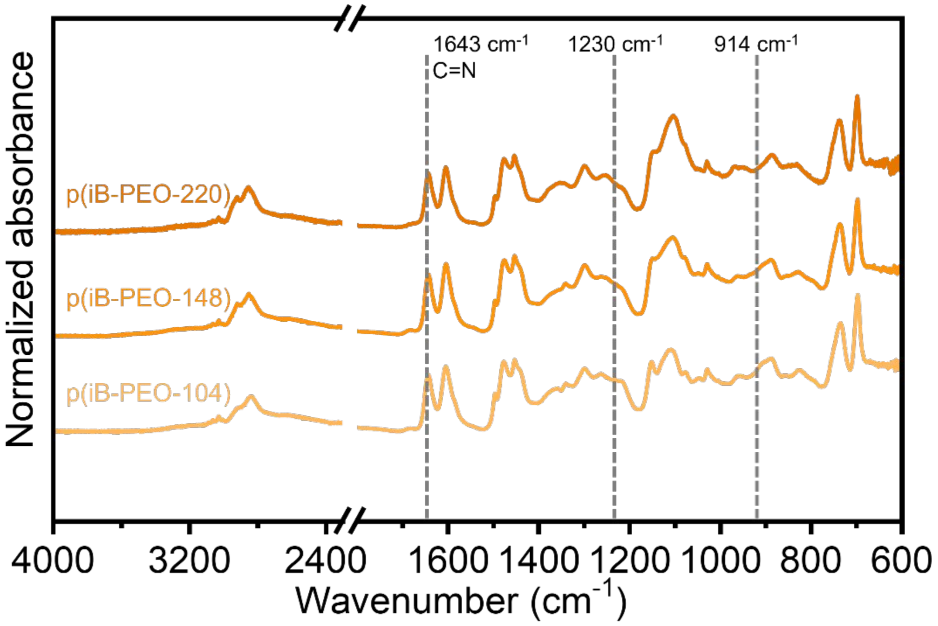

Figure S39. ATR-FTIR spectra of p(iB-PEO-X) networks, cross-linked at 180 °C for 2 h.

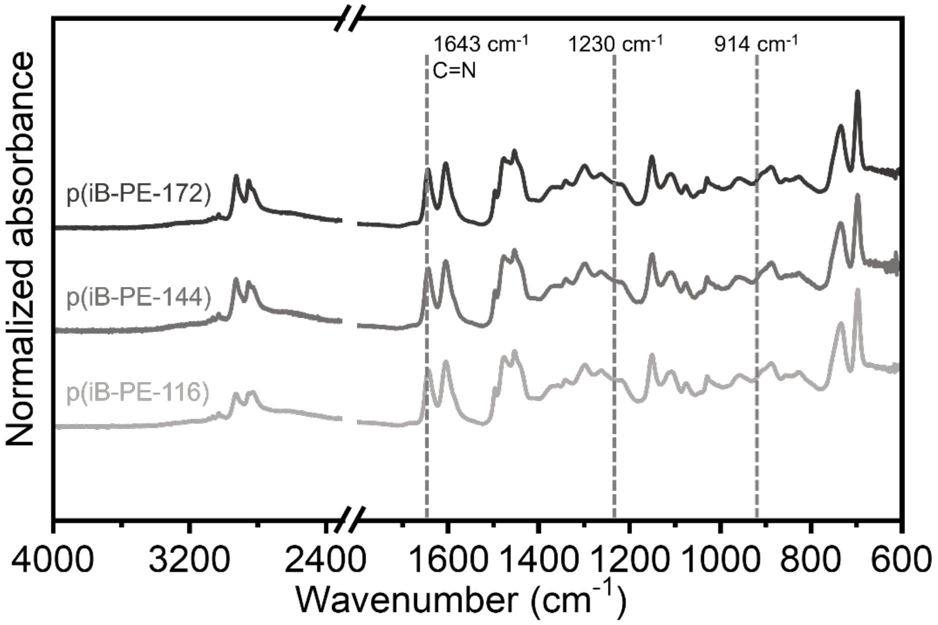

Figure S40. ATR-FTIR spectra of p(iB-PE-X) networks, cross-linked at 180 °C for 2 h.

Thermogravimetric Analysis and Differential Scanning Calorimetry of p(iBOX) Networks

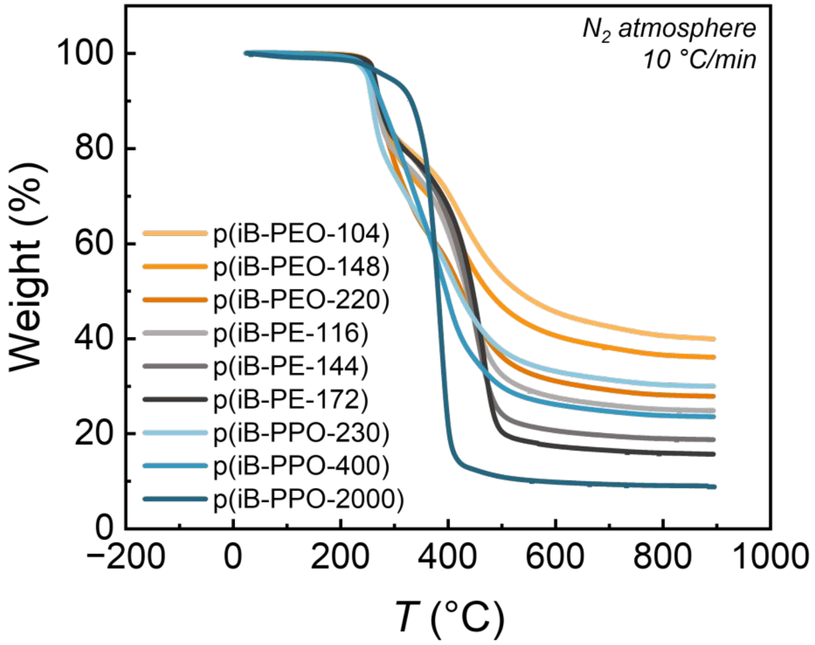

Figure S41. Thermogravimetric analysis of each p(iBOX) network.

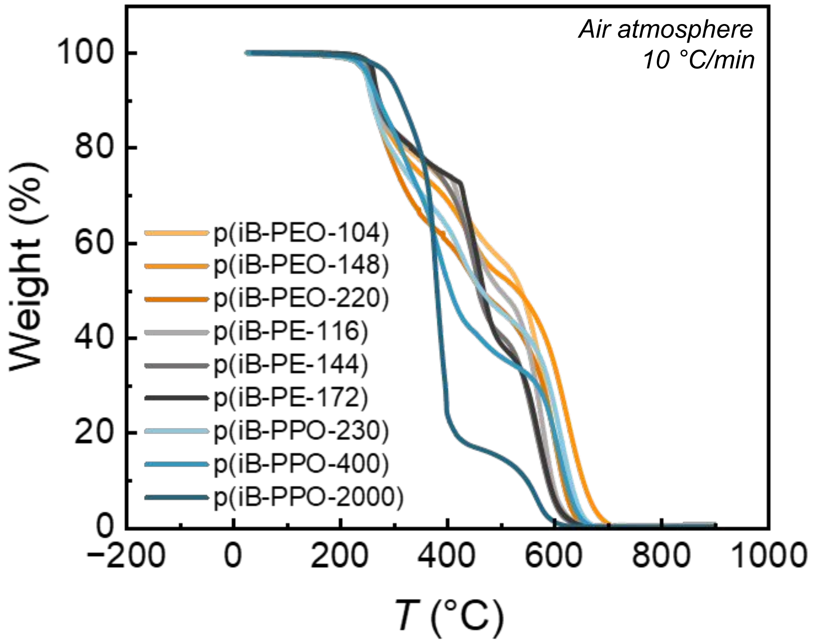

Figure S42. Thermogravimetric analysis of each p(iBOX) network.

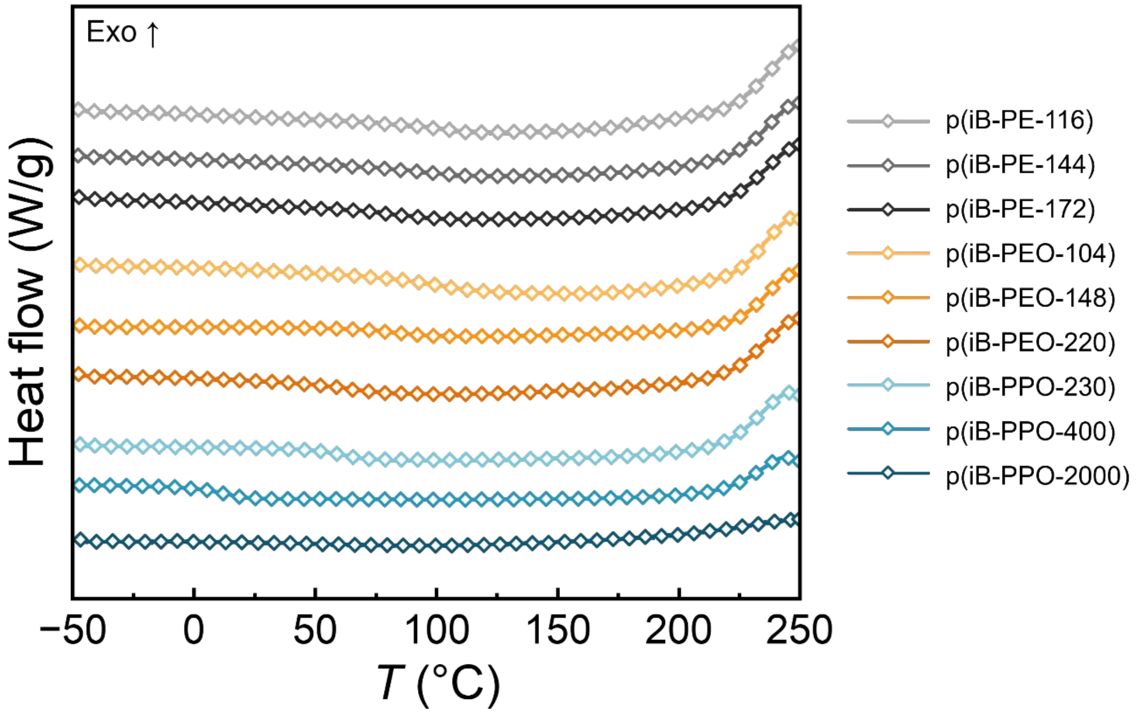

Figure S43. Differential scanning calorimetry of each p(iBOX) network.

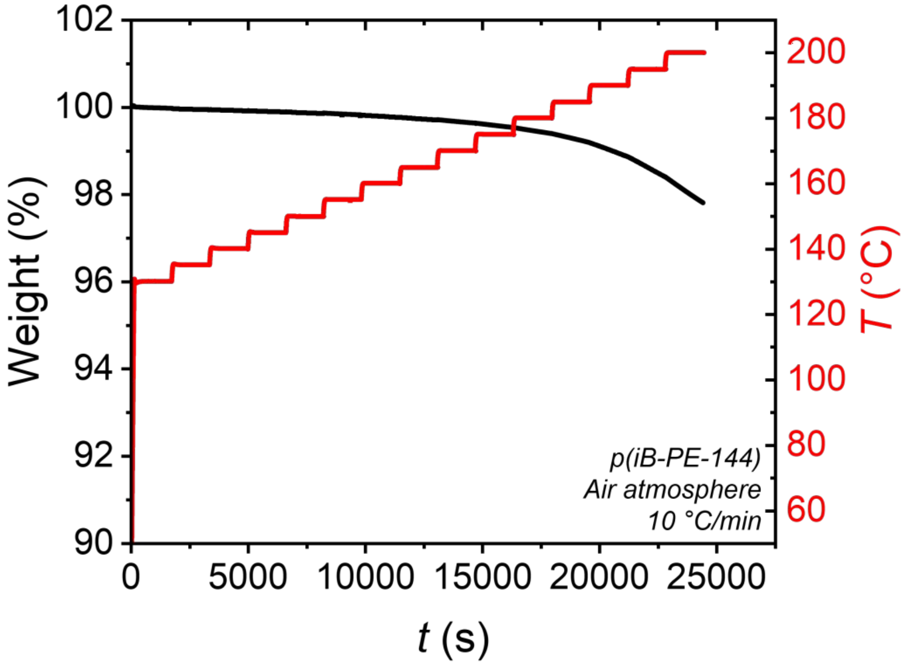

Figure S44. Step TGA thermogram simulating the temperature profile in stress relaxation for p(iB-PE-144).

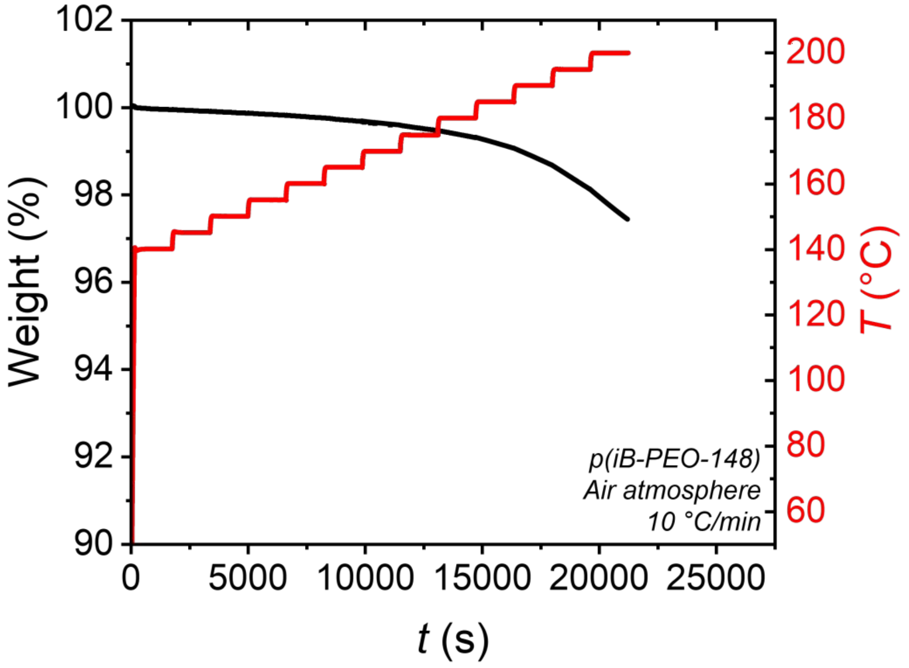

Figure S45. Step TGA thermogram simulating the temperature profile in stress relaxation for p(iB-PEO-148).

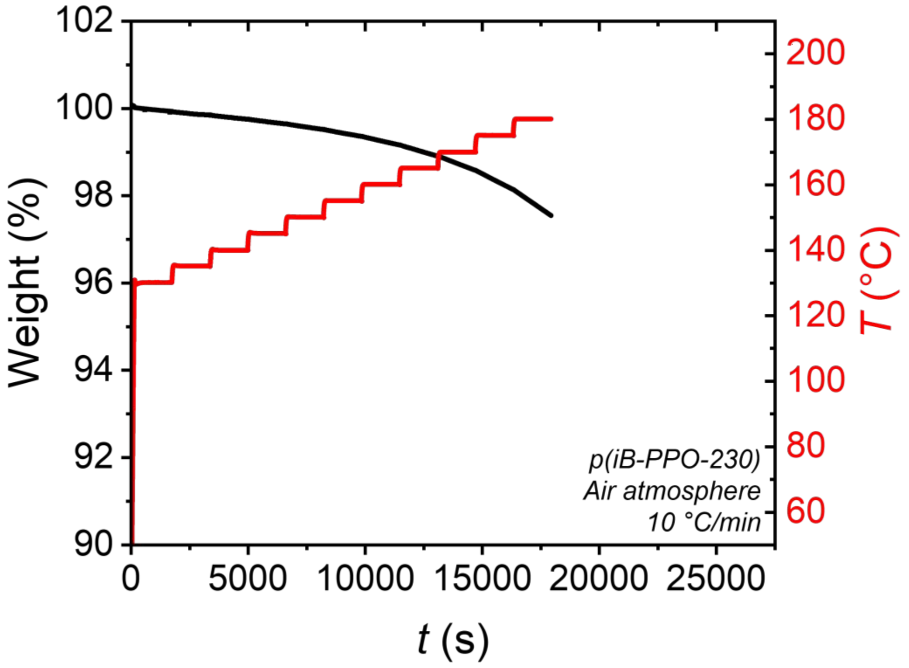

Figure S46. Step TGA thermogram simulating the temperature profile in stress relaxation for p(iB-PPO-230).

Dynamic Mechanical Analysis

Cross-link density was estimated according to the classical theory of rubber elasticity using $\nu_e = \frac{E'_\mathrm{R}}{3\phi RT}$, where R is the ideal gas constant, ϕ is the front factor (approximated to 1.0 for an ideal rubber), and T is the absolute temperature at T_g + 30 °C.[1,2]

Table S1. Summary of Dynamic Mechanical Analysis Results.

**p(iBOX) Network**	$\boldsymbol{T_g}$ [a] (°C)	***E'* at 25 °C** (MPa)	$\boldsymbol{E'_{rubbery}}$ [b] **at** $\boldsymbol{T_g}$ **+ 30 °C** (MPa)	$\boldsymbol{\nu_e}$ [c] (mol/m^3)
p(iB-PPO-230)	88.6	2883	7.08	724
p(iB-PPO-400)	34.4	292	5.25	623
p(iB-PPO-2000)	-39.4	0.54	1.08	164
p(iB-PEO-104)	140.1	3149	40.0	3619
p(iB-PEO-148)	115.5	2961	31.9	3059
p(iB-PEO-220)	83.9	2345	28.5	2948
p(iB-PE-116)	131.8	2819	49.0	4516
p(iB-PE-144)	120.1	2685	46.6	4403
p(iB-PE-172)	116.1	2643	37.5	3587

[a]Taken as the temperature corresponding to the peak of tanδ. [b]$E'_{rubbery}$ is the storage modulus from DMA in tension at T_g + 30 °C. [c]ν_e is the cross-link density per unit volume estimated through by the theory of rubbery elasticity.

S4. Rheological Characterization

Strain Amplitude Sweep Experiments for p(iBOX) Networks

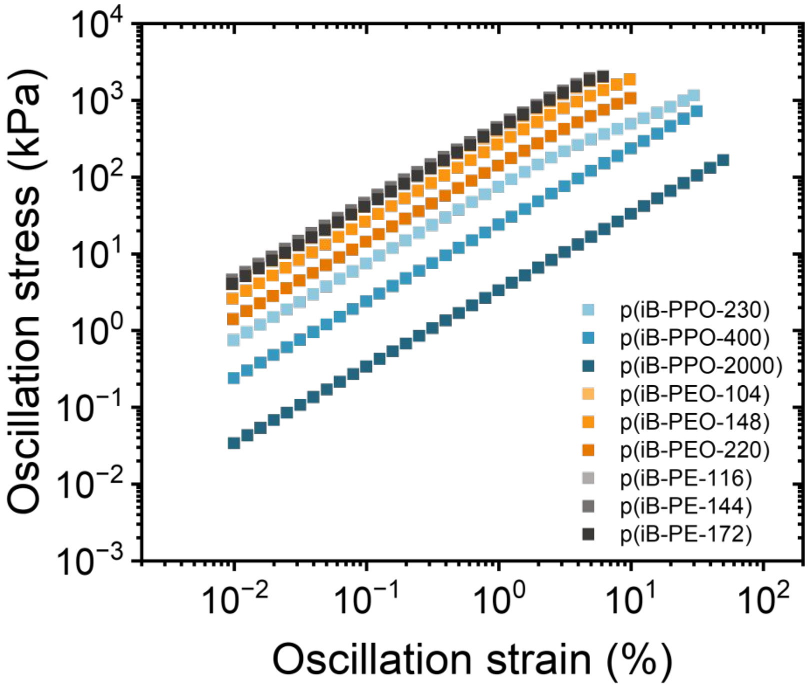

Figure S47. Strain amplitude sweeps for all networks performed at the lowest temperature used for stress relaxation and SAOS for each respective network.

Temperature Conditions for Rheological Experiments

For most networks, stress relaxation data at the first two temperatures was not used for fitting due to inconsistent relaxation behavior with increasing temperature, which is believed to be due to the lack of a pre-conditioning process like that used by Ricarte and coworkers.[3,4] However, for p(iB-PE-172), the data at the first three temperature intervals were omitted for fitting, while the data at the first four temperature intervals were omitted in the cases of p(iB-PE-144) and p(iB-PPO-2000). While polymerization *in-situ* facilitates excellent adhesion of the plates and cross-linked polymer, we postulate that internal stress accumulated during cross-linking may relax and contribute to the overall stress relaxation behavior observed at the first few temperature intervals. To maintain consistency of the thermal history between the small-amplitude oscillatory shear (SAOS) and stress relaxation experiments, the SAOS experiments were performed at the same temperatures, and the omission of the same temperature intervals was exercised. The temperature ranges for the experiments and fitting are defined in Table S2.

Table S2. Temperature Ranges for Rheological Experiments and Fitting.

p(iBOX) Network	Experimental Temperature Range	Temperature Range for Fitting
p(iB-PPO-230)	130 – 180 °C	140 – 200 °C
p(iB-PPO-400)	100 – 180 °C	110 – 180 °C
p(iB-PPO-2000)	50 – 180 °C	70 – 180 °C
p(iB-PEO-104)	165 – 200 °C	175 – 200 °C
p(iB-PEO-148)	140 – 200 °C	150 – 200 °C
p(iB-PEO-220)	130 – 200 °C	140 – 200 °C
p(iB-PE-116)	160 – 200 °C	170 – 200 °C
p(iB-PE-144)	140 – 200 °C	160 – 200 °C
p(iB-PE-172)	130 – 200 °C	145 – 200 °C

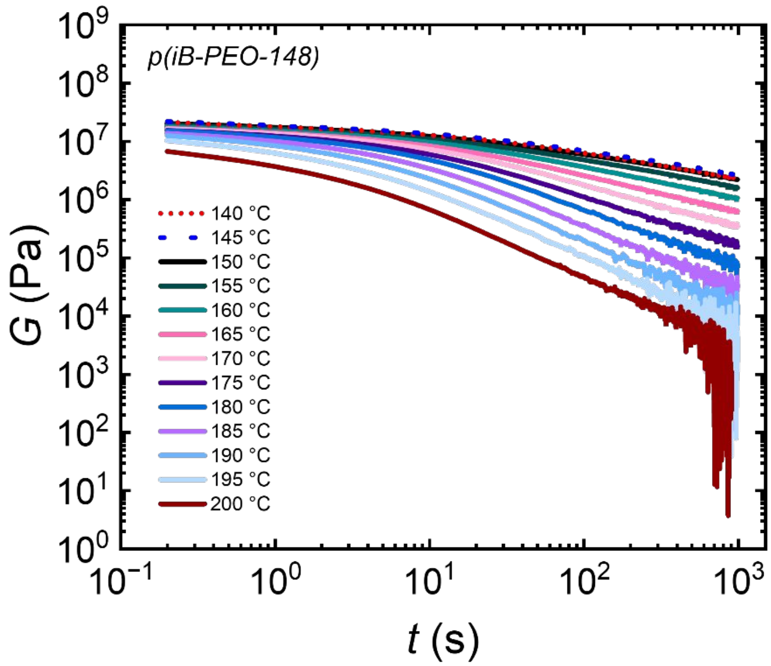

Figure S48. Stress relaxation of p(iB-PEO-148) including the curves, corresponding to 140 °C and 145 °C, excluded from fitting.

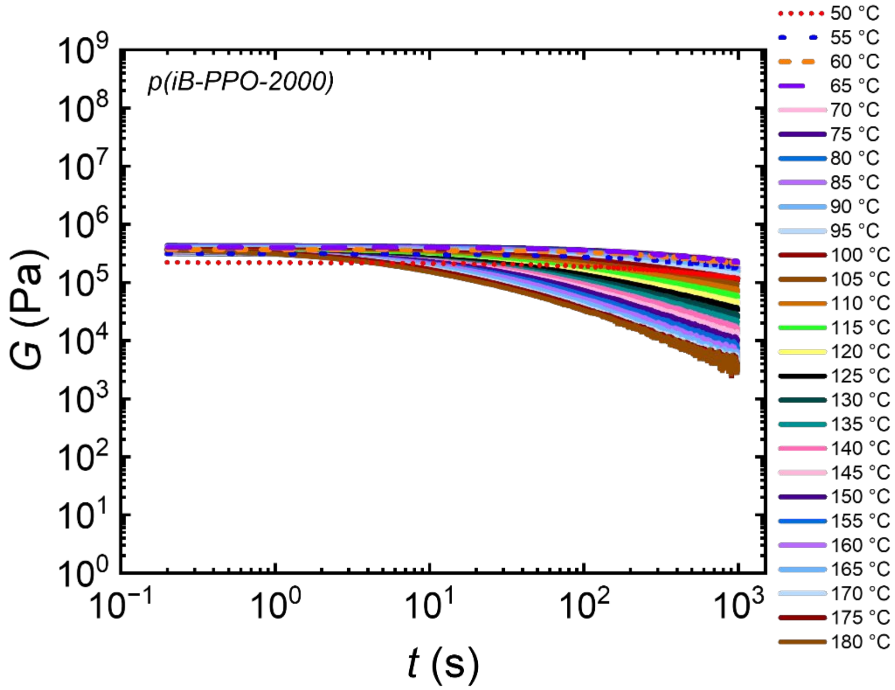

Figure S49. Stress relaxation of p(iB-PPO-2000) including the curves, corresponding to 50 – 65 °C, excluded from fitting.

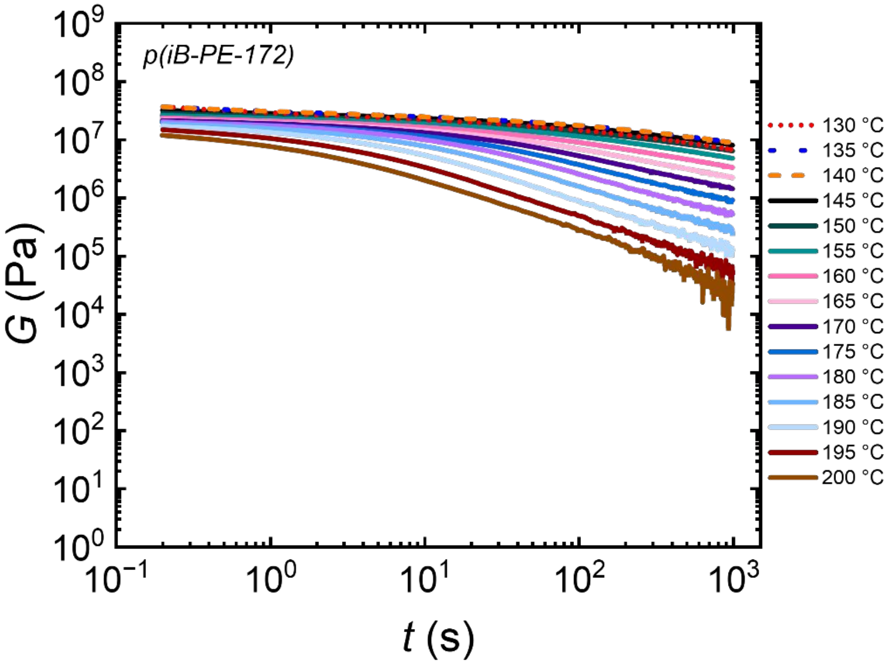

Figure S50. Stress relaxation of p(iB-PE-172) including the curves, corresponding to 130 – 140 °C, excluded from fitting.

Stress Relaxation of p(iBOX) Networks

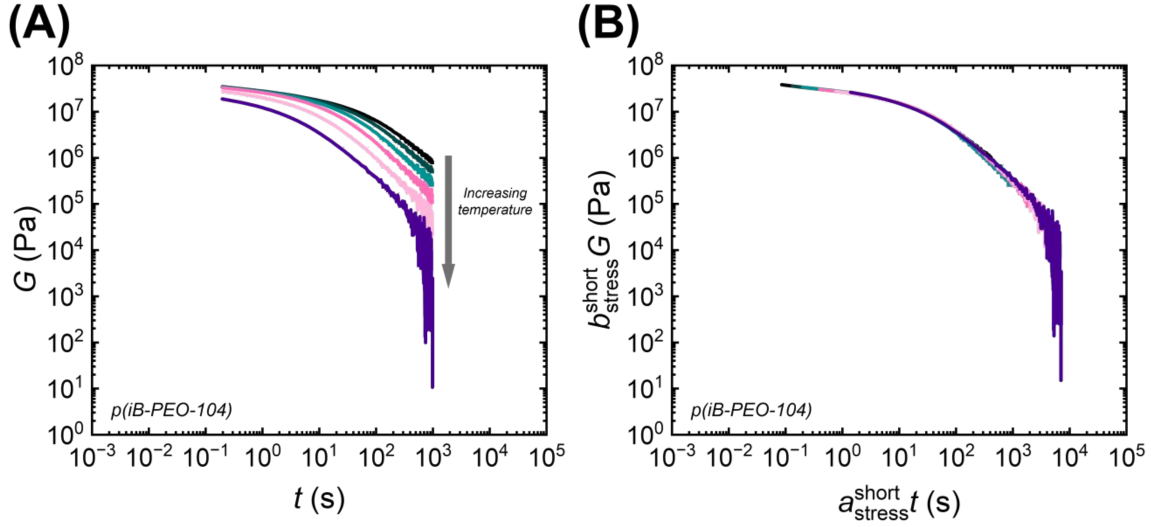

Figure S51. Stress relaxation of p(iB-PEO-104) with relaxation modulus (G) plotted against time (t) (A) as directly measured and (B) with a^{short}_{stress} and b^{short}_{stress} applied. T_{ref} = 185 °C.

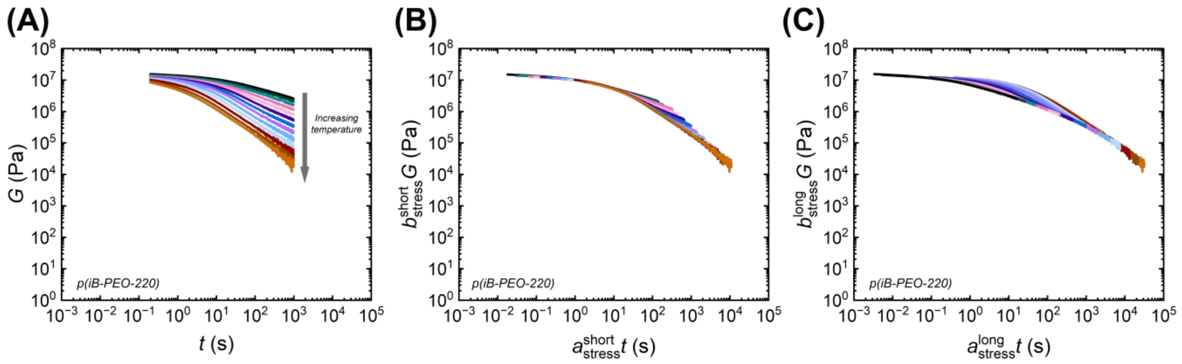

Figure S52. Stress relaxation of p(iB-PEO-220) with G plotted against t (A) as directly measured, (B) with a^{short}_{stress} and b^{short}_{stress} applied, and (C) with a^{long}_{stress} and b^{long}_{stress} applied. T_{ref} = 170 °C.

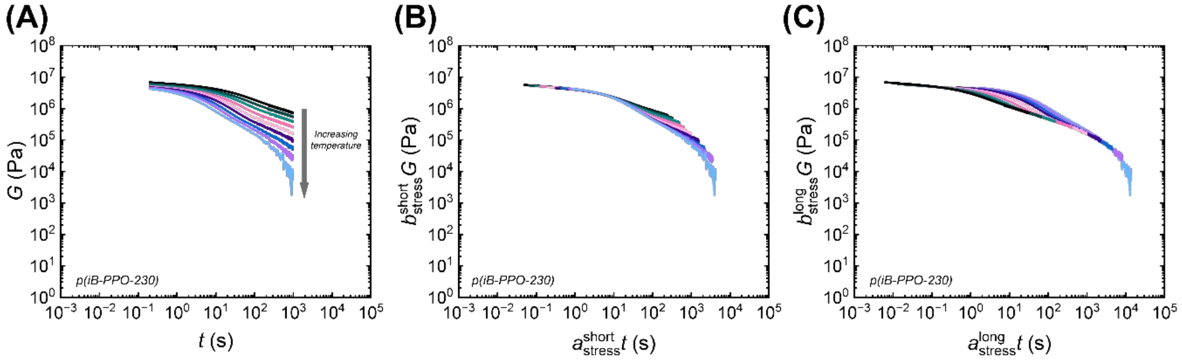

Figure S53. Stress relaxation of p(iB-PPO-230) with G plotted against t (A) as directly measured, (B) with a^{short}_{stress} and b^{short}_{stress} applied, and (C) with a^{long}_{stress} and b^{long}_{stress} applied. T_{ref} = 160 °C.

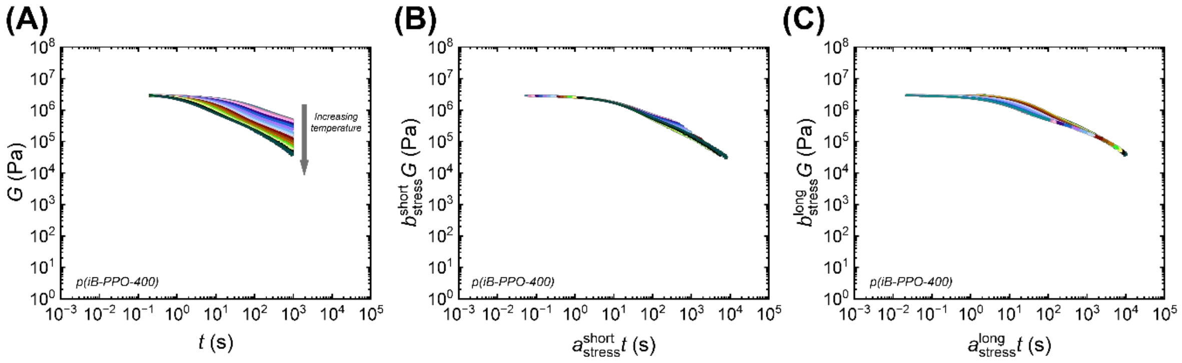

Figure S54. Stress relaxation of p(iB-PPO-400) with G plotted against t (A) as directly measured, (B) with $a_{\mathrm{stress}}^{\mathrm{short}}$ and $b_{\mathrm{stress}}^{\mathrm{short}}$ applied, and (C) with $a_{\mathrm{stress}}^{\mathrm{long}}$ and $b_{\mathrm{stress}}^{\mathrm{long}}$ applied. $T_{\mathrm{ref}} = 140$ °C.

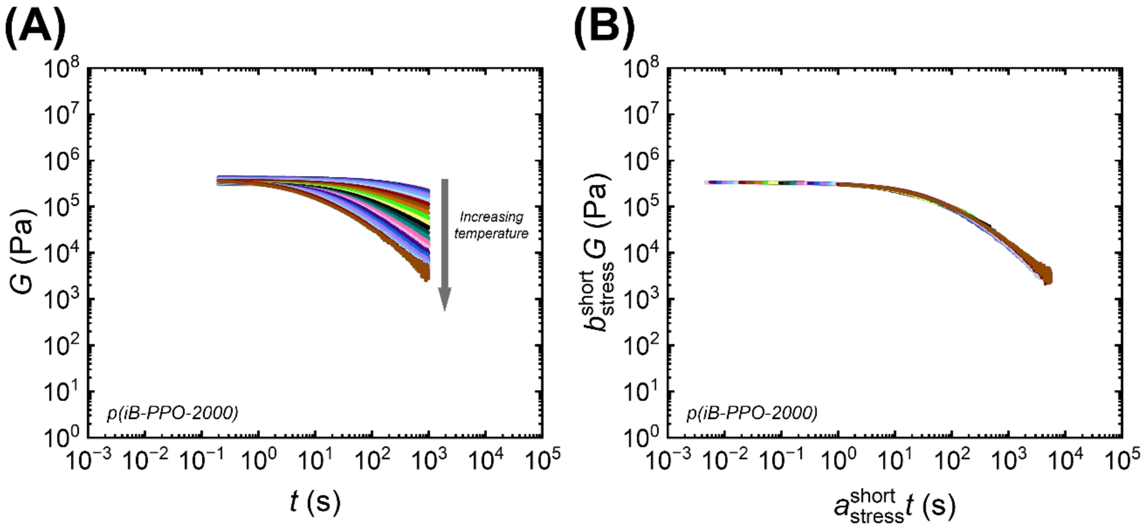

Figure S55. Stress relaxation of p(iB-PPO-2000) with G plotted against t (A) as directly measured and (B) with $a_{\mathrm{stress}}^{\mathrm{short}}$ and $b_{\mathrm{stress}}^{\mathrm{short}}$ applied. $T_{\mathrm{ref}} = 125$ °C.

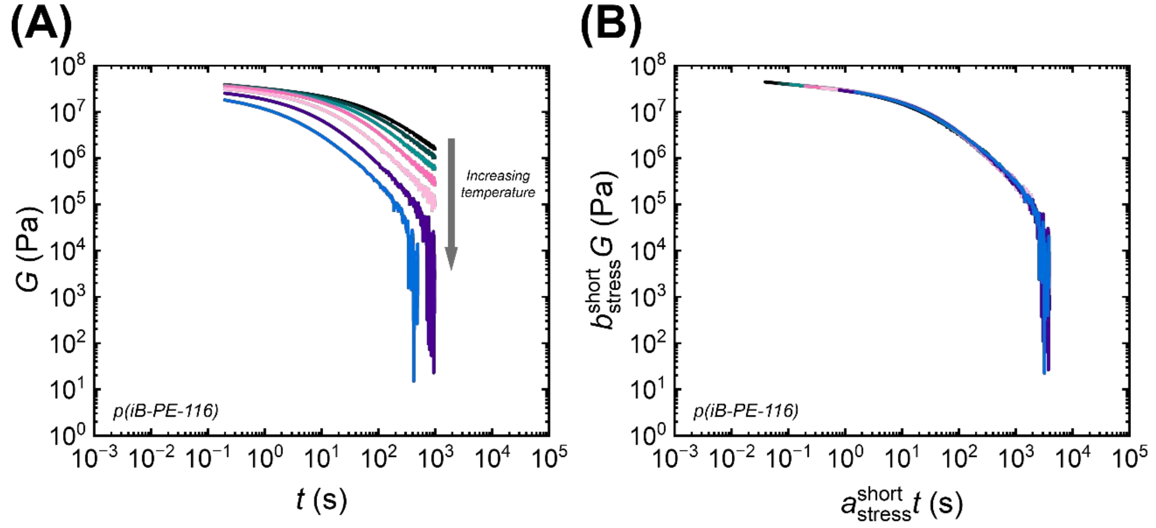

Figure S56. Stress relaxation of p(iB-PE-116) with G plotted against t (A) as directly measured and (B) with $a_{\text{stress}}^{\text{short}}$ and $b_{\text{stress}}^{\text{short}}$ applied. $T_{\text{ref}} = 185$ °C.

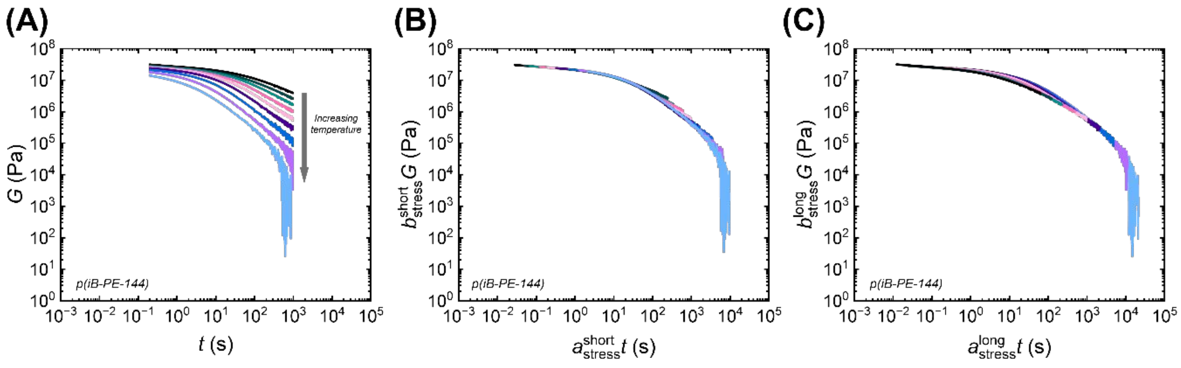

Figure S57. Stress relaxation of p(iB-PE-144) with G plotted against t (A) as directly measured, (B) with $a_{\text{stress}}^{\text{short}}$ and $b_{\text{stress}}^{\text{short}}$ applied, and (C) with $a_{\text{stress}}^{\text{long}}$ and $b_{\text{stress}}^{\text{long}}$ applied. $T_{\text{ref}} = 180$ °C.

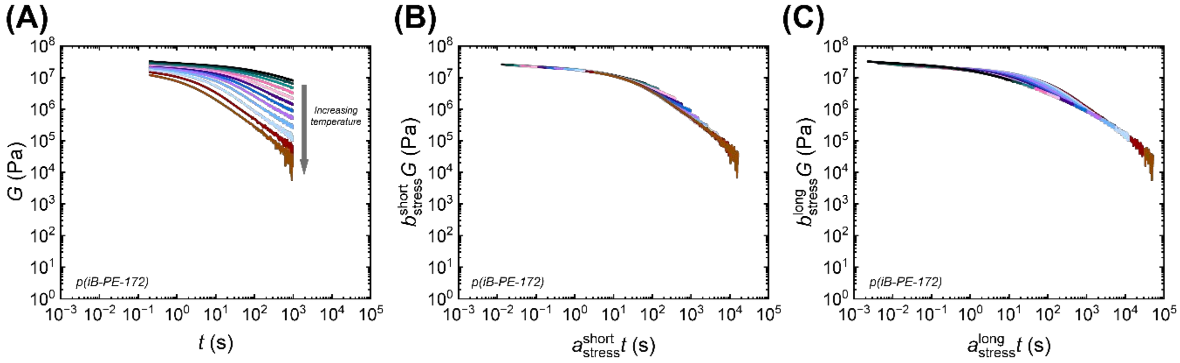

Figure S58. Stress relaxation of p(iB-PE-172) with G plotted against t (A) as directly measured, (B) with $a_{\text{stress}}^{\text{short}}$ and $b_{\text{stress}}^{\text{short}}$ applied, and (C) with $a_{\text{stress}}^{\text{long}}$ and $b_{\text{stress}}^{\text{long}}$ applied. $T_{\text{ref}} = 175$ °C.

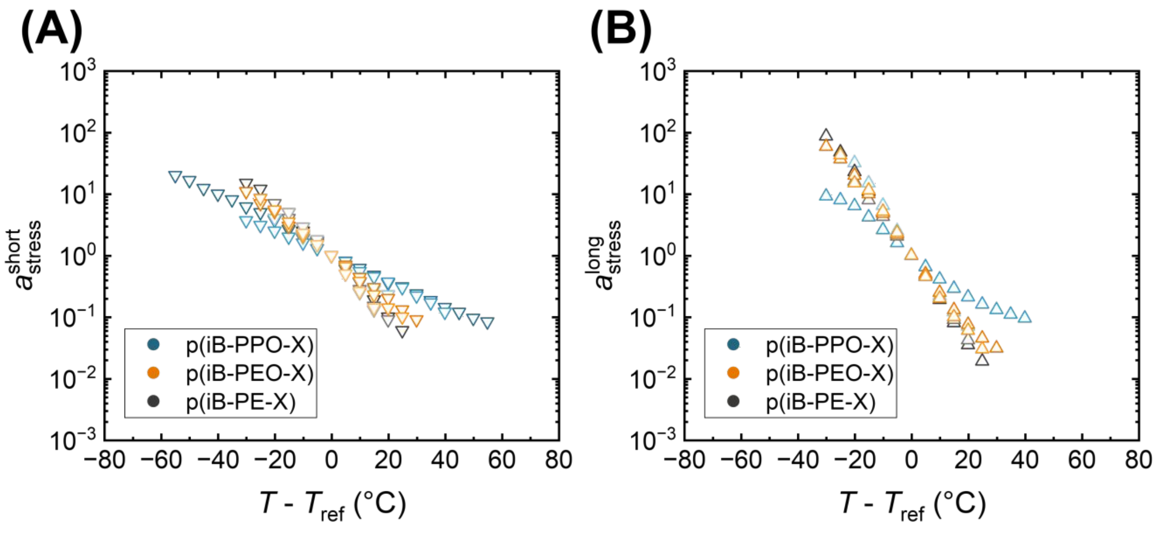

Figure S59. Horizontal shift factors for superposing stress relaxation data at (A) short timescales and (B) long timescales.

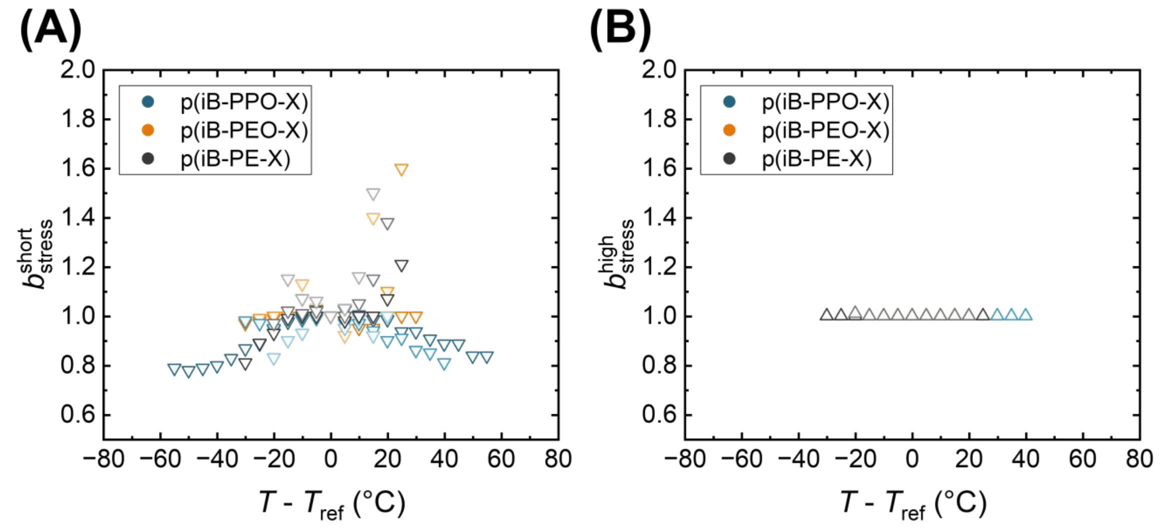

Figure S60. Vertical shift factors for superposing stress relaxation data at (A) short timescales and (B) long timescales.

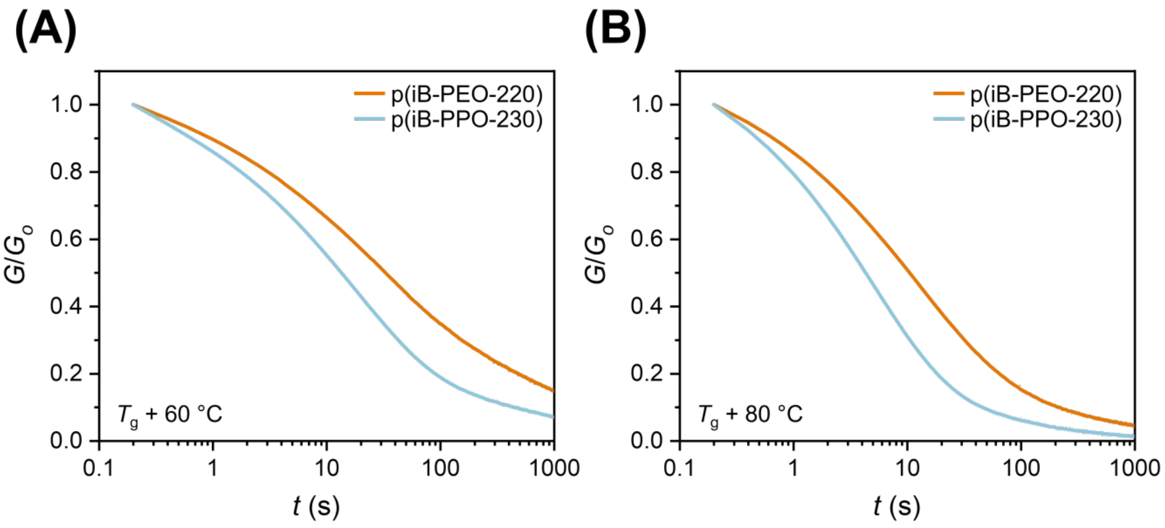

Figure S61. Normalized stress relaxation curves, with *G* normalized by initial relaxation modulus (G_0) plotted against time, for p(iB-PEO-220) and p(iB-PPO-230) at (A) T_g + 60 °C and (B) T_g + 80 °C.

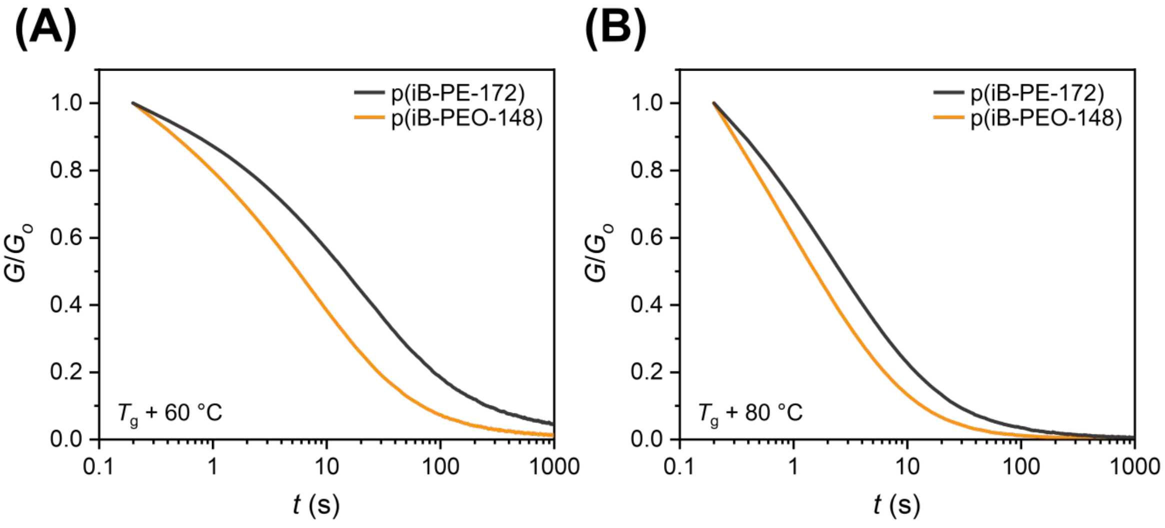

Figure S62. Normalized stress relaxation curves, with G/G_0 plotted against time, for p(iB-PEO-220) and p(iB-PPO-230) at (A) T_g + 60 °C and (B) T_g + 80 °C.

Small-Amplitude Oscillatory Shear (SAOS) of p(iBOX) Networks

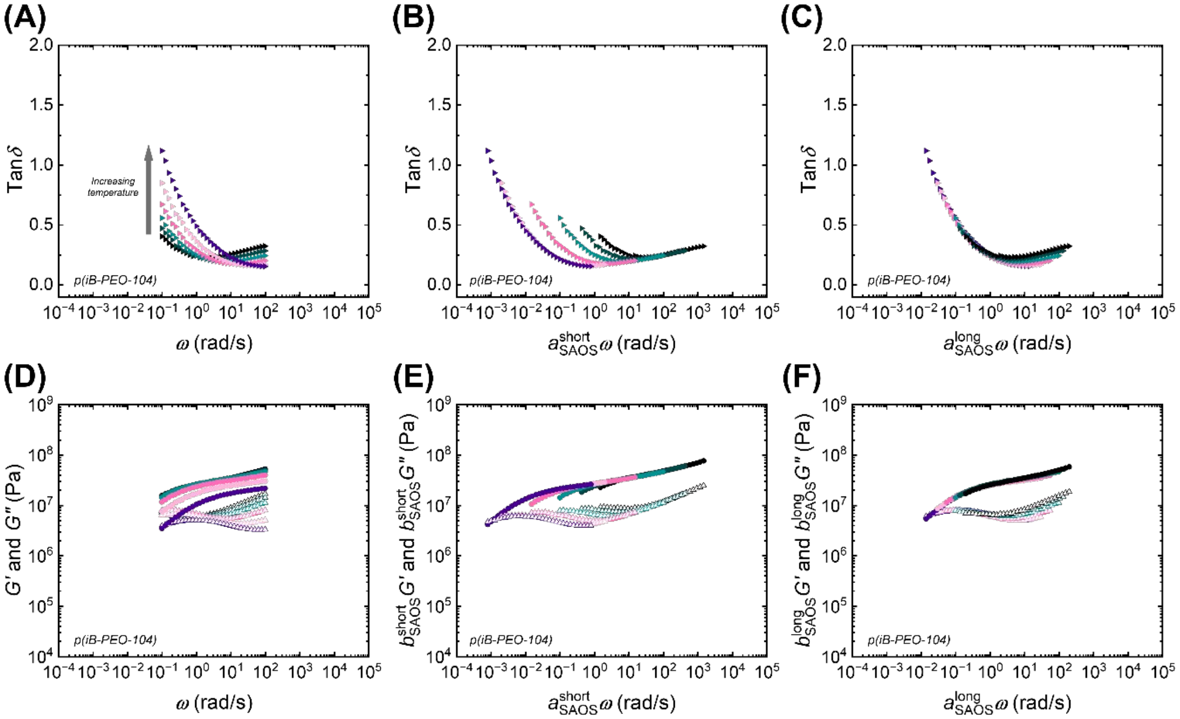

Figure S63. Small-amplitude oscillatory shear of p(iB-PEO-104) with tanδ plotted against angular frequency (ω) (A) as measured, (B) shifted horizontally with $a_{\mathrm{SAOS}}^{\mathrm{short}}$ and $b_{\mathrm{SAOS}}^{\mathrm{short}}$, and (C) with $a_{\mathrm{SAOS}}^{\mathrm{long}}$ and $b_{\mathrm{SAOS}}^{\mathrm{long}}$. Shear storage ($G'$) and loss ($G''$) moduli plotted against ω (D) as obtained, (E) with $a_{\mathrm{SAOS}}^{\mathrm{short}}$ and $b_{\mathrm{SAOS}}^{\mathrm{short}}$ applied, and (F) with $a_{\mathrm{SAOS}}^{\mathrm{long}}$ and $b_{\mathrm{SAOS}}^{\mathrm{long}}$ shifts applied. T_{ref} = 185 °C.

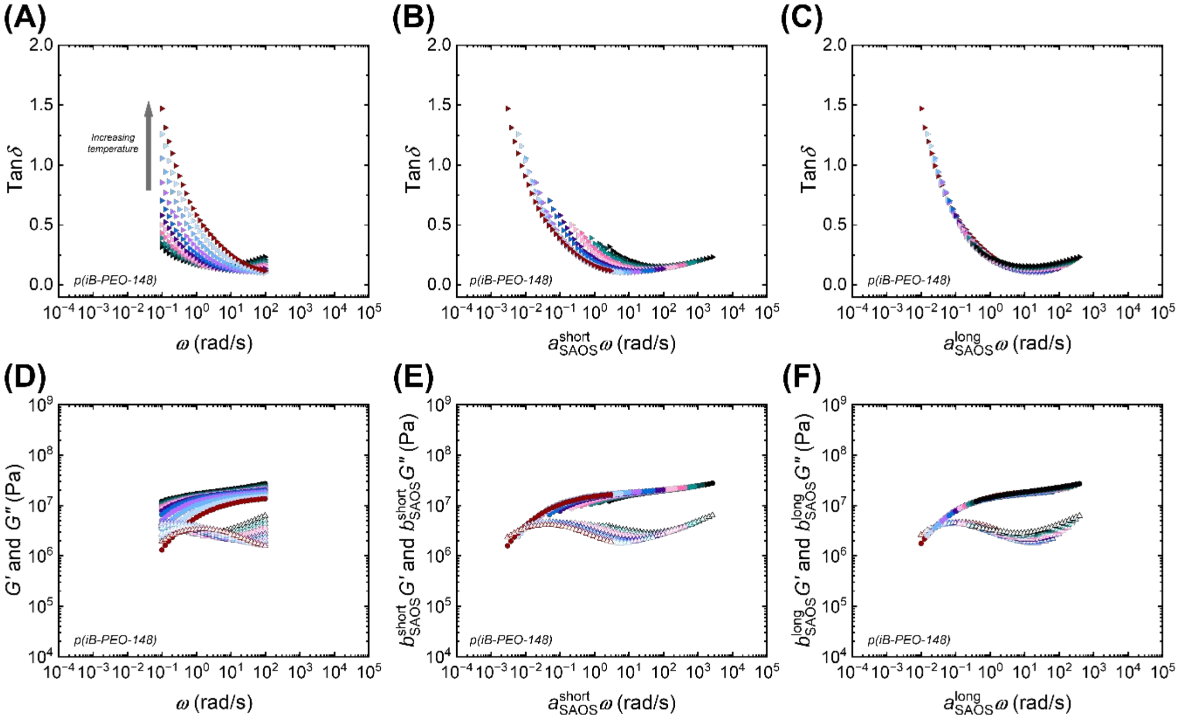

Figure S64. Small-amplitude oscillatory shear of p(iB-PEO-148) with tanδ plotted against ω (A) as measured, (B) shifted horizontally with $a_{\mathrm{SAOS}}^{\mathrm{short}}$ and $b_{\mathrm{SAOS}}^{\mathrm{short}}$, and (C) with $a_{\mathrm{SAOS}}^{\mathrm{long}}$ and $b_{\mathrm{SAOS}}^{\mathrm{long}}$. Storage ($G'$) and loss ($G''$) moduli plotted against ω (D) as obtained, (E) with $a_{\mathrm{SAOS}}^{\mathrm{short}}$ and $b_{\mathrm{SAOS}}^{\mathrm{short}}$ applied, and (F) with $a_{\mathrm{SAOS}}^{\mathrm{long}}$ and $b_{\mathrm{SAOS}}^{\mathrm{long}}$ shifts applied. T_{ref} = 175 °C.

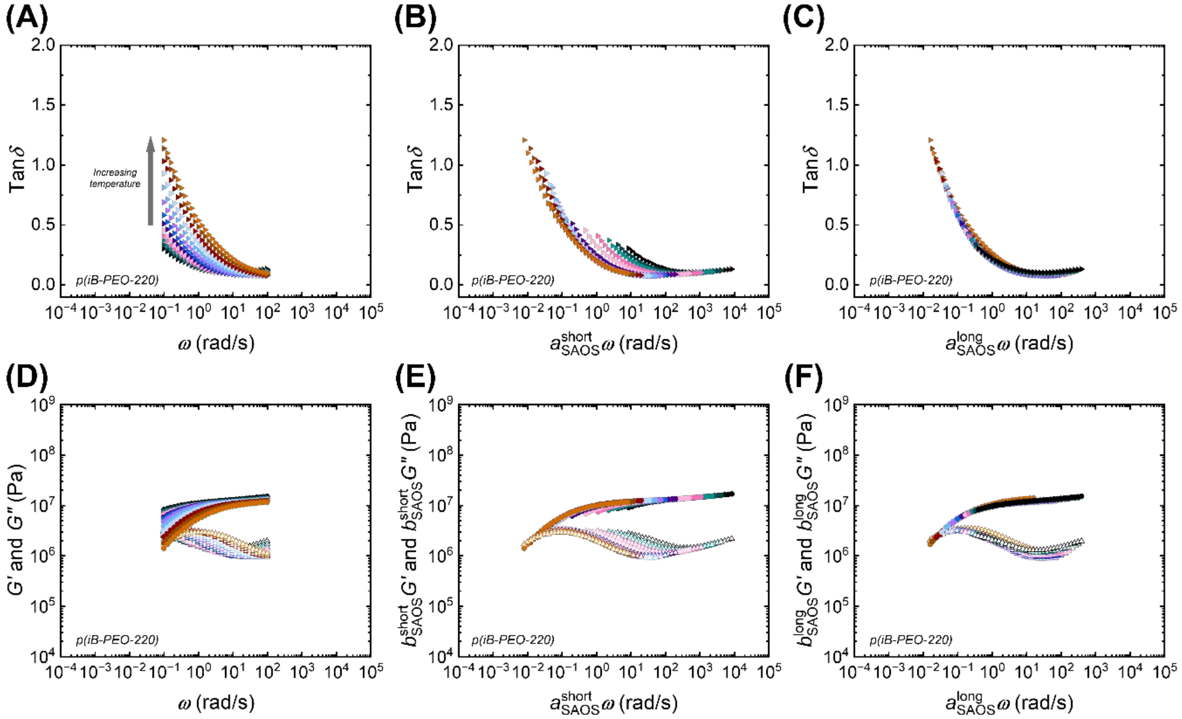

Figure S65. Small-amplitude oscillatory shear of p(iB-PEO-220) with tanδ plotted against ω (A) as measured, (B) shifted horizontally with $a_{\mathrm{SAOS}}^{\mathrm{short}}$ and $b_{\mathrm{SAOS}}^{\mathrm{short}}$, and (C) with $a_{\mathrm{SAOS}}^{\mathrm{long}}$ and $b_{\mathrm{SAOS}}^{\mathrm{long}}$. Shear storage ($G'$) and loss ($G''$) moduli plotted against ω (D) as obtained, (E) with $a_{\mathrm{SAOS}}^{\mathrm{short}}$ and $b_{\mathrm{SAOS}}^{\mathrm{short}}$ applied, and (F) with $a_{\mathrm{SAOS}}^{\mathrm{long}}$ and $b_{\mathrm{SAOS}}^{\mathrm{long}}$ shifts applied. T_{ref} = 170 °C.

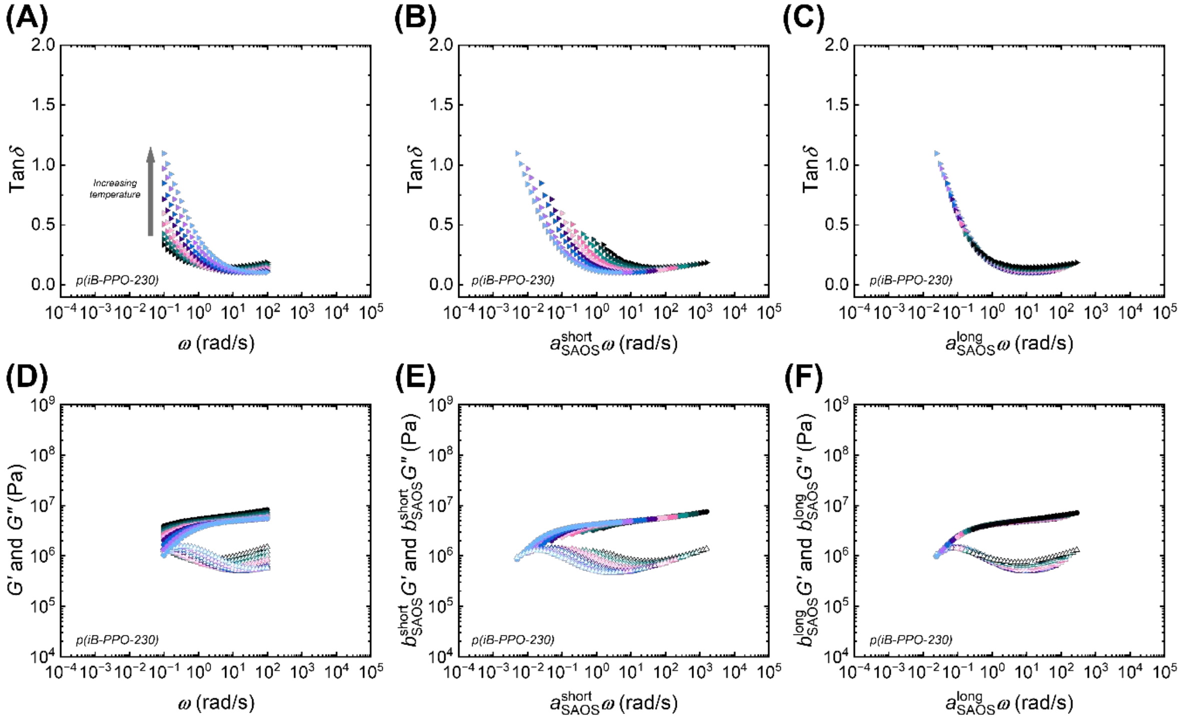

Figure S66. Small-amplitude oscillatory shear of p(iB-PPO-230) with tanδ plotted against ω (A) as measured, (B) shifted horizontally with $a_{\mathrm{SAOS}}^{\mathrm{short}}$ and $b_{\mathrm{SAOS}}^{\mathrm{short}}$, and (C) with $a_{\mathrm{SAOS}}^{\mathrm{long}}$ and $b_{\mathrm{SAOS}}^{\mathrm{long}}$. Shear storage ($G'$) and loss ($G''$) moduli plotted against ω (D) as obtained, (E) with $a_{\mathrm{SAOS}}^{\mathrm{short}}$ and $b_{\mathrm{SAOS}}^{\mathrm{short}}$ applied, and (F) with $a_{\mathrm{SAOS}}^{\mathrm{long}}$ and $b_{\mathrm{SAOS}}^{\mathrm{long}}$ shifts applied. T_{ref} = 160 °C.

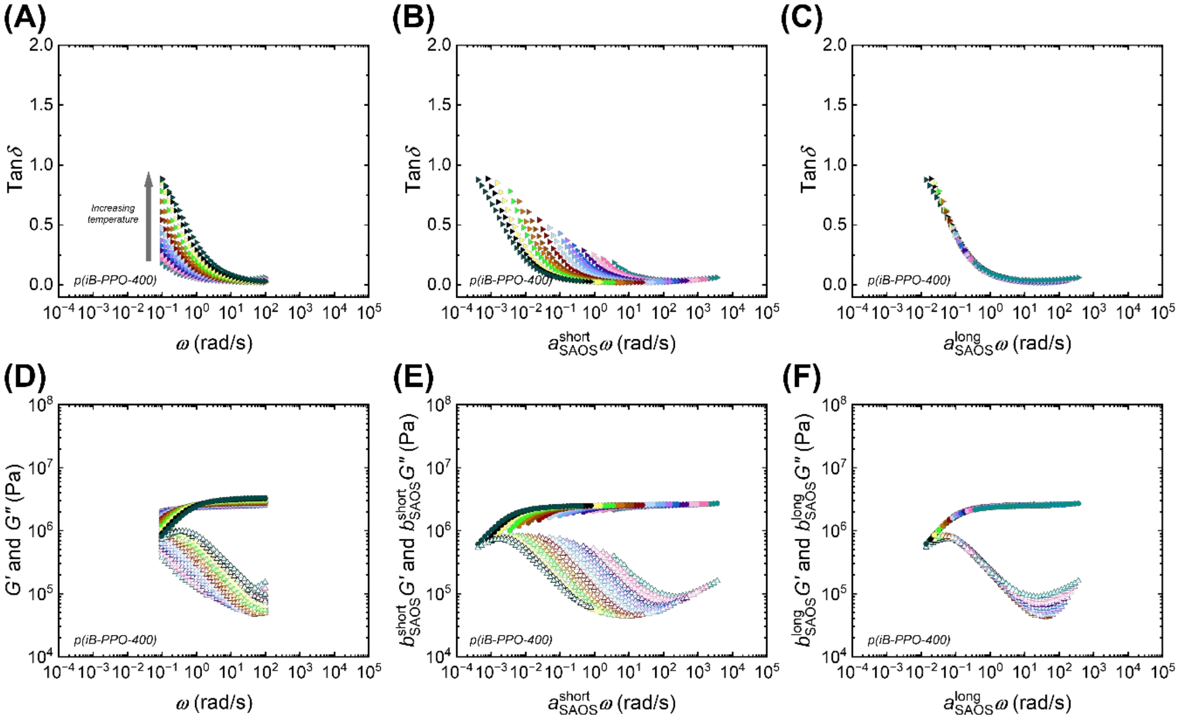

Figure S67. Small-amplitude oscillatory shear of p(iB-PPO-400) with tanδ plotted against ω (A) as measured, (B) shifted horizontally with $a_{\mathrm{SAOS}}^{\mathrm{short}}$ and $b_{\mathrm{SAOS}}^{\mathrm{short}}$, and (C) with $a_{\mathrm{SAOS}}^{\mathrm{long}}$ and $b_{\mathrm{SAOS}}^{\mathrm{long}}$. Shear storage ($G'$) and loss ($G''$) moduli plotted against ω (D) as obtained, (E) with $a_{\mathrm{SAOS}}^{\mathrm{short}}$ and $b_{\mathrm{SAOS}}^{\mathrm{short}}$ applied, and (F) with $a_{\mathrm{SAOS}}^{\mathrm{long}}$ and $b_{\mathrm{SAOS}}^{\mathrm{long}}$ shifts applied. T_{ref} = 140 °C.

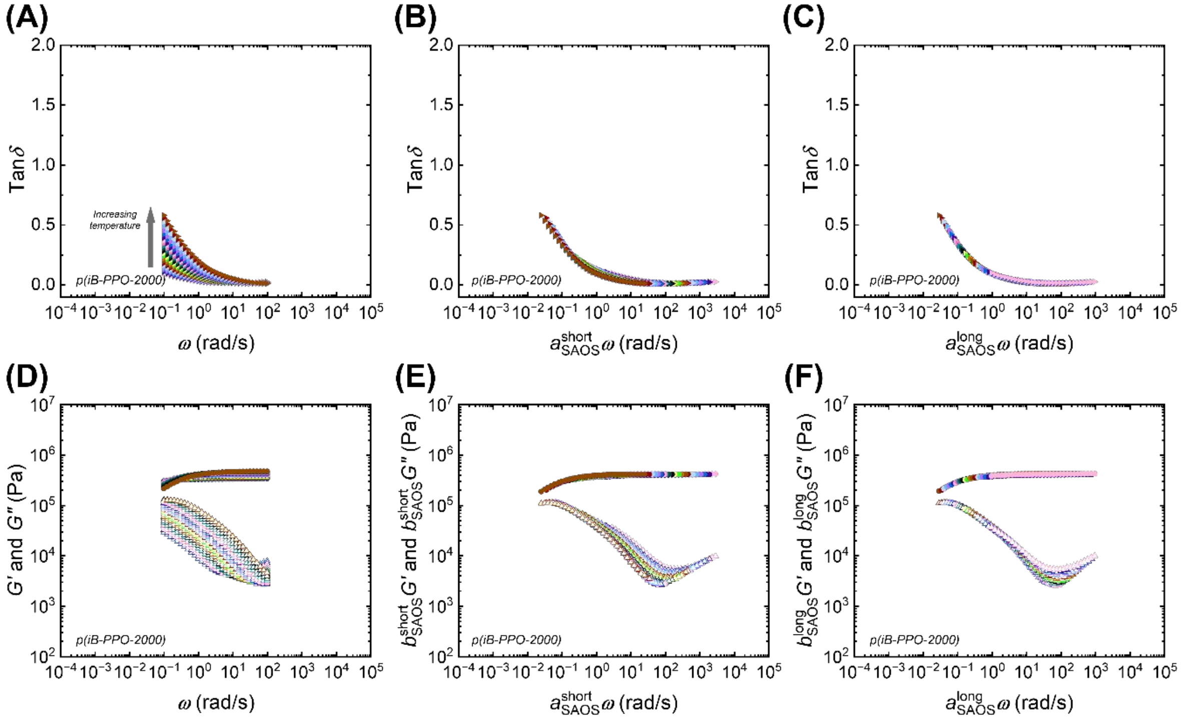

Figure S68. Small-amplitude oscillatory shear of p(iB-PPO-2000) with tanδ plotted against ω (A) as measured, (B) shifted horizontally with $a_{\mathrm{SAOS}}^{\mathrm{short}}$ and $b_{\mathrm{SAOS}}^{\mathrm{short}}$, and (C) with $a_{\mathrm{SAOS}}^{\mathrm{long}}$ and $b_{\mathrm{SAOS}}^{\mathrm{long}}$. Shear storage ($G'$) and loss ($G''$) moduli plotted against ω (D) as obtained, (E) with $a_{\mathrm{SAOS}}^{\mathrm{short}}$ and $b_{\mathrm{SAOS}}^{\mathrm{short}}$ applied, and (F) with $a_{\mathrm{SAOS}}^{\mathrm{long}}$ and $b_{\mathrm{SAOS}}^{\mathrm{long}}$ shifts applied. T_{ref} = 125 °C.

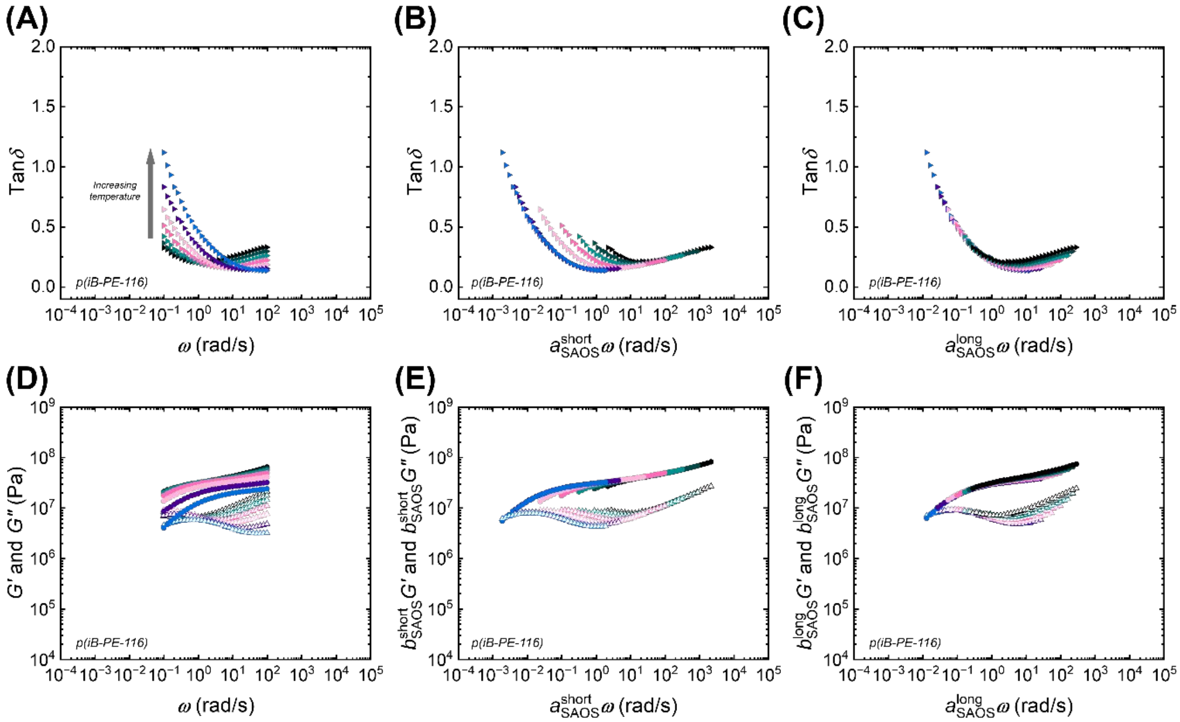

Figure S69. Small-amplitude oscillatory shear of p(iB-PE-116) with tanδ plotted against ω (A) as measured, (B) shifted horizontally with a^{short}_{SAOS} and b^{short}_{SAOS}, and (C) with a^{long}_{SAOS} and b^{long}_{SAOS}. Shear storage (G') and loss (G'') moduli plotted against ω (D) as obtained, (E) with a^{short}_{SAOS} and b^{short}_{SAOS} applied, and (F) with a^{long}_{SAOS} and b^{long}_{SAOS} shifts applied. T_{ref} = 185 °C.

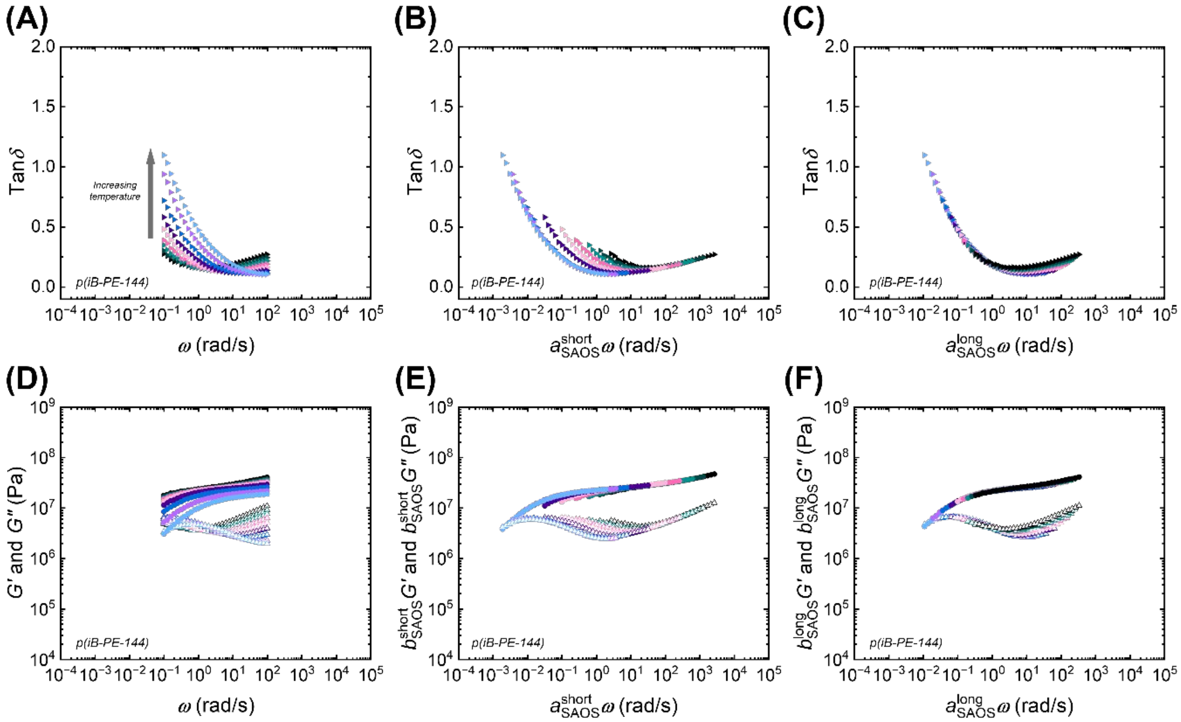

Figure S70. Small-amplitude oscillatory shear of p(iB-PE-144) with tanδ plotted against ω (A) as measured, (B) shifted horizontally with $a_{\mathrm{SAOS}}^{\mathrm{short}}$ and $b_{\mathrm{SAOS}}^{\mathrm{short}}$, and (C) with $a_{\mathrm{SAOS}}^{\mathrm{long}}$ and $b_{\mathrm{SAOS}}^{\mathrm{long}}$. Shear storage ($G'$) and loss ($G''$) moduli plotted against ω (D) as obtained, (E) with $a_{\mathrm{SAOS}}^{\mathrm{short}}$ and $b_{\mathrm{SAOS}}^{\mathrm{short}}$ applied, and (F) with $a_{\mathrm{SAOS}}^{\mathrm{long}}$ and $b_{\mathrm{SAOS}}^{\mathrm{long}}$ shifts applied. T_{ref} = 180 °C.

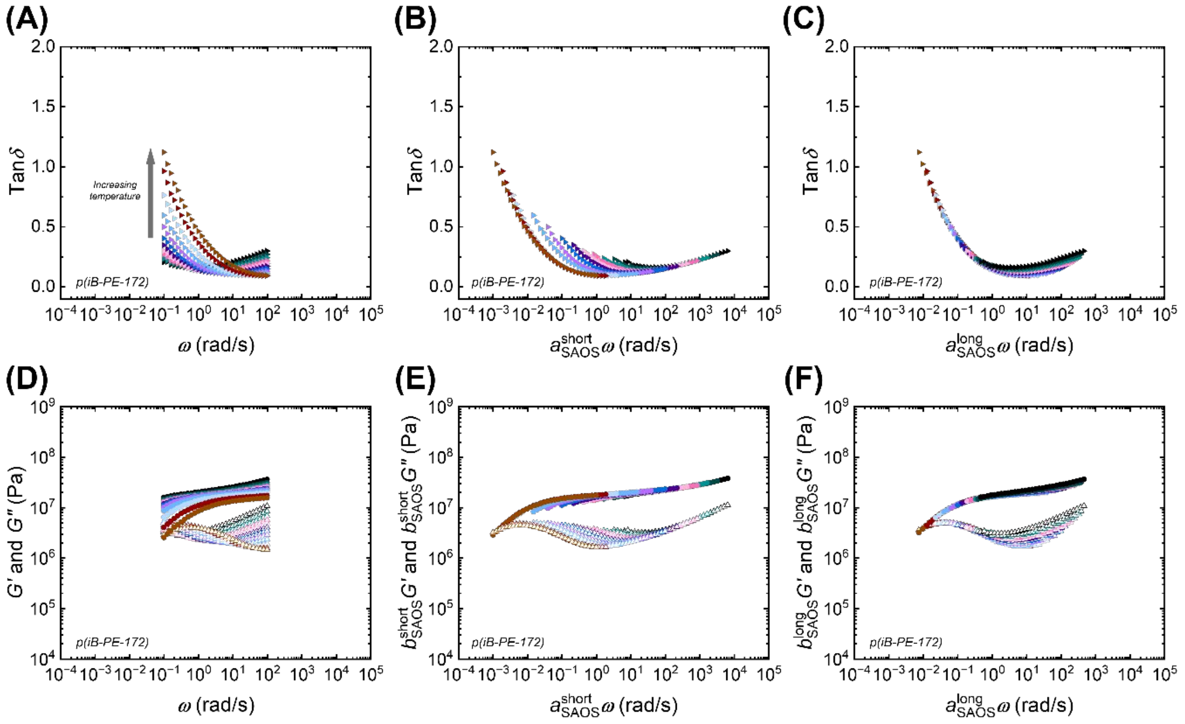

Figure S71. Small-amplitude oscillatory shear of p(iB-PE-172) with tanδ plotted against ω (A) as measured, (B) shifted horizontally with $a_{\mathrm{SAOS}}^{\mathrm{short}}$ and $b_{\mathrm{SAOS}}^{\mathrm{short}}$, and (C) with $a_{\mathrm{SAOS}}^{\mathrm{long}}$ and $b_{\mathrm{SAOS}}^{\mathrm{long}}$. Shear storage ($G'$) and loss ($G''$) moduli plotted against ω (D) as obtained, (E) with $a_{\mathrm{SAOS}}^{\mathrm{short}}$ and $b_{\mathrm{SAOS}}^{\mathrm{short}}$ applied, and (F) with $a_{\mathrm{SAOS}}^{\mathrm{long}}$ and $b_{\mathrm{SAOS}}^{\mathrm{long}}$ shifts applied. T_{ref} = 175 °C.

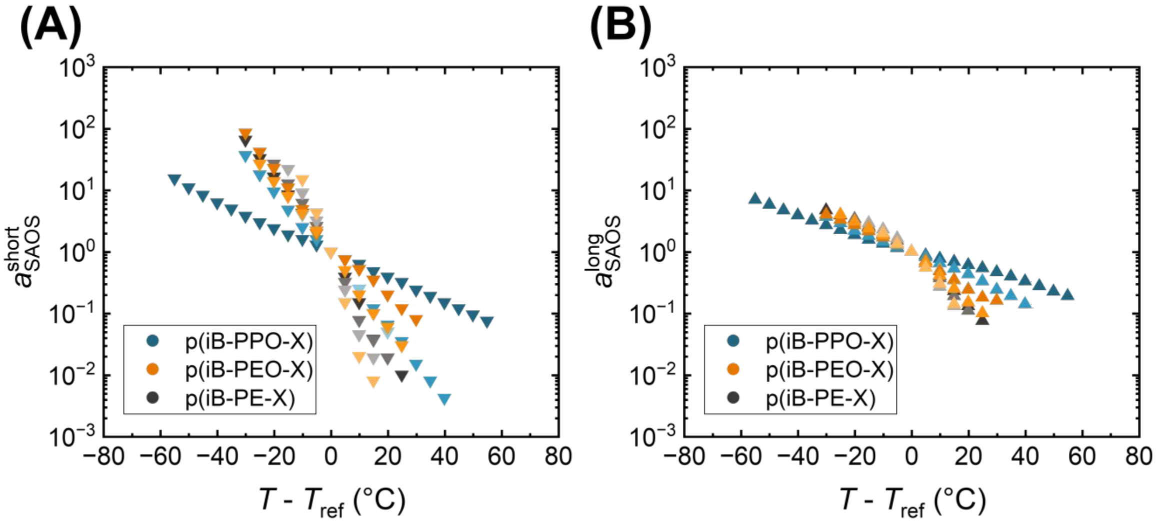

Figure S72. Horizontal shift factors for superposing SAOS data at (A) short timescales and (B) long timescales.

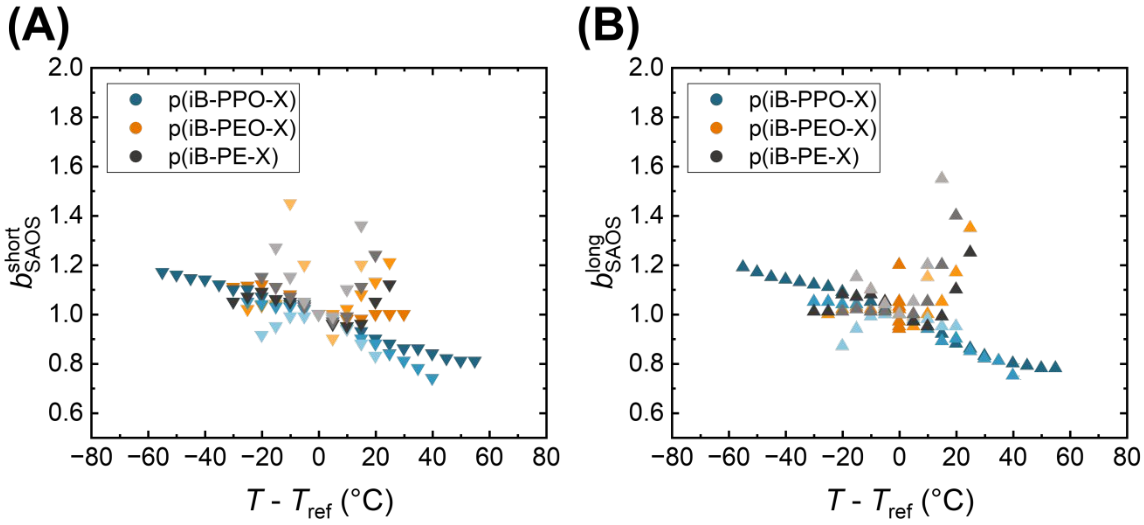

Figure S73. Vertical shift factors for superposing SAOS data at (A) short timescales and (B) long timescales.

Estimation of Apparent Activation Energy

Table S3. Estimates of Apparent Activation Energy (E_a) for Each p(iBOX) Network.

p(iBOX) Network	$E_{a,SAOS}^{short}$ (kJ/mol)	$E_{a,stress}^{short}$ (kJ/mol)	$E_{a,SAOS}^{long}$ (kJ/mol)	$E_{a,stress}^{long}$ (kJ/mol)	E_a^{MM} [b] (kJ/mol)
p(iB-PPO-230)	223.9 ± 11.3	116.3 ± 8.0	96.6 ± 8.8	246.7 ± 13.1	102.4 ± 2.3
p(iB-PPO-400)	184.7 ± 8.8	70.3 ± 4.0	64.7 ± 3.9	105.7 ± 7.1	70.3 ± 0.6
p(iB-PPO-2000)	61.2 ± 1.8	67.9 ± 2.2	40.6 ± 1.1	N/A[a]	70.1 ± 1.8
p(iB-PEO-104)	560.9 ± 75.6	200.6 ± 32.3	185.0 ± 44.5	N/A[a]	156.5 ± 12.5
p(iB-PEO-148)	231.5 ± 11.5	149.1 ± 6.9	123.7 ± 15.3	244.7 ± 12.0	112.9 ± 3.0
p(iB-PEO-220)	190.1 ± 13.3	129.8 ± 4.1	92.9 ± 8.0	217.5 ± 8.2	104.9 ± 1.5
p(iB-PE-116)	427.2 ± 52.2	213.0 ± 20.8	182.4 ± 42.2	N/A[a]	150.6 ± 9.5
p(iB-PE-144)	325.9 ± 30.1	186.7 ± 16.7	145.8 ± 25.7	254.4 ± 14.8	143.0 ± 4.5
p(iB-PE-172)	269.2 ± 23.6	168.3 ± 12.8	123.1 ± 19.2	259.4 ± 11.5	140.6 ± 4.7

[a]Not obtained due to the superposition of stress relaxation data with one set of horizontal shift factors.
[b]Obtained from the linear fit of the characteristic relaxation times, τ^{MM}, using the method outlined below.
Note: Values are reported as the estimate ± the 95% confidence interval.

E_a^{MM} was estimated using $\frac{G(t)}{G_{0.2\,s}} = e^{-t/\tau^{MM}}$, where τ^{MM} was taken as the point where $\frac{G(t)}{G_{0.2\,s}} = \frac{1}{e}$. The value of τ^{MM} was determined at all temperatures. For all p(iBOX) networks, E_a^{MM} was estimated over the same temperature range listed in Table S2 by applying a linear fit to ln(τ^{MM}) vs. 1000/T, where $E_a^{MM} = (slope) \times R$.

References

(1) Zhao, S.; Abu-Omar, M. M. Recyclable and Malleable Epoxy Thermoset Bearing Aromatic Imine Bonds. *Macromolecules* **2018**, *51* (23), 9816–9824. https://doi.org/10.1021/acs.macromol.8b01976.
(2) Iijima, T.; Yoshioka, N.; Tomoi, M. Effect of Cross-Link Density on Modification of Epoxy Resins with Reactive Acrylic Elastomers. *European Polymer Journal* **1992**, *28* (6), 573–581. https://doi.org/10.1016/0014-3057(92)90025-W.
(3) Barzycki, D. C.; Ezzeddine, D.; Shanbhag, S.; Ricarte, R. G. Linear Viscoelasticity of Polystyrene Vitrimers: Segmental Motions and the Slow Arrhenius Process. *Macromolecules* **2025**, *58* (8), 3949–3963. https://doi.org/10.1021/acs.macromol.4c03161.
(4) Ricarte, R. G.; Shanbhag, S.; Ezzeddine, D.; Barzycki, D.; Fay, K. Time–Temperature Superposition of Polybutadiene Vitrimers. *Macromolecules* **2023**, *56* (17), 6806–6817. https://doi.org/10.1021/acs.macromol.3c00883.
